# Benzyl-Modified Quinolone-Aminopyrimidine Hybrids with Potent Anti-MRSA Activity and a High Barrier to Resistance

**DOI:** 10.3390/molecules31101633

**Published:** 2026-05-12

**Authors:** Xinghua Xu, Renhua Fan, Qiuqin He

**Affiliations:** Department of Chemistry, Fudan University, 2005 Songhu Road, Yangpu District, Shanghai 200438, China; 23210220045@m.fudan.edu.cn (X.X.);

**Keywords:** quinolone-aminopyrimidine hybrids, MRSA, resistance

## Abstract

A series of novel quinolone–aminopyrimidine hybrids were designed and synthesized through benzyl-position modification. Among them, **A3** and **A5** exhibited promising anti-MRSA activity, low cytotoxicity, and a low propensity for resistance development. Notably, **A5** showed favorable metabolic stability in both HLMs and RLMs, whereas **A3** displayed excessively prolonged half-lives, suggesting a potential risk of in vivo accumulation. Overall, **A5** was identified as a promising lead compound for further antibacterial development.

## 1. Introduction

The rapid global expansion of antimicrobial resistance (AMR) continues to erode the clinical utility of existing antibacterial agents, thereby substantially narrowing the therapeutic options available for the treatment of infectious diseases [1]. As resistant bacteria emerge and spread at an alarming rate, AMR has become one of the most pressing challenges to global public health, contributing to increased morbidity, mortality, and healthcare expenditure [2,3]. Among multidrug-resistant pathogens, methicillin-resistant *Staphylococcus aureus* (MRSA) remains a high-priority target in antibacterial drug discovery because of its widespread prevalence in both healthcare- and community-associated infections and the limited efficacy of currently available treatment options [4,5]. MRSA can cause a wide range of infections, from mild skin and soft tissue infections to severe invasive diseases such as pneumonia, bacteremia, endocarditis, and sepsis [6,7]. Owing to its major clinical and public health impact, MRSA remains classified as a high-priority pathogen on the World Health Organization (WHO) Bacterial Priority Pathogens List (2024) [8].

MRSA has evolved resistance to multiple classes of antibacterial agents, including β-lactams, macrolides, fluoroquinolones, glycopeptides, and oxazolidinones, which greatly complicates clinical treatment and limits the effectiveness of available therapeutic regimens [9,10,11]. Among current anti-MRSA therapies, vancomycin has long served as a cornerstone for the treatment of severe MRSA infections. However, the continued emergence of vancomycin-intermediate *S. aureus* (VISA) and vancomycin-resistant *S. aureus* (VRSA) has progressively diminished its therapeutic value and raised serious concerns regarding the sustainability of existing treatment strategies [12,13,14]. Although several agents targeting MRSA are currently under clinical investigation, the development of novel antibiotics has not kept pace with the rapid rise of resistance [15,16,17]. Consequently, there is an urgent need for novel anti-MRSA agents with a reduced propensity to induce resistance.

### Design and Chemistry

**Figure 1:**
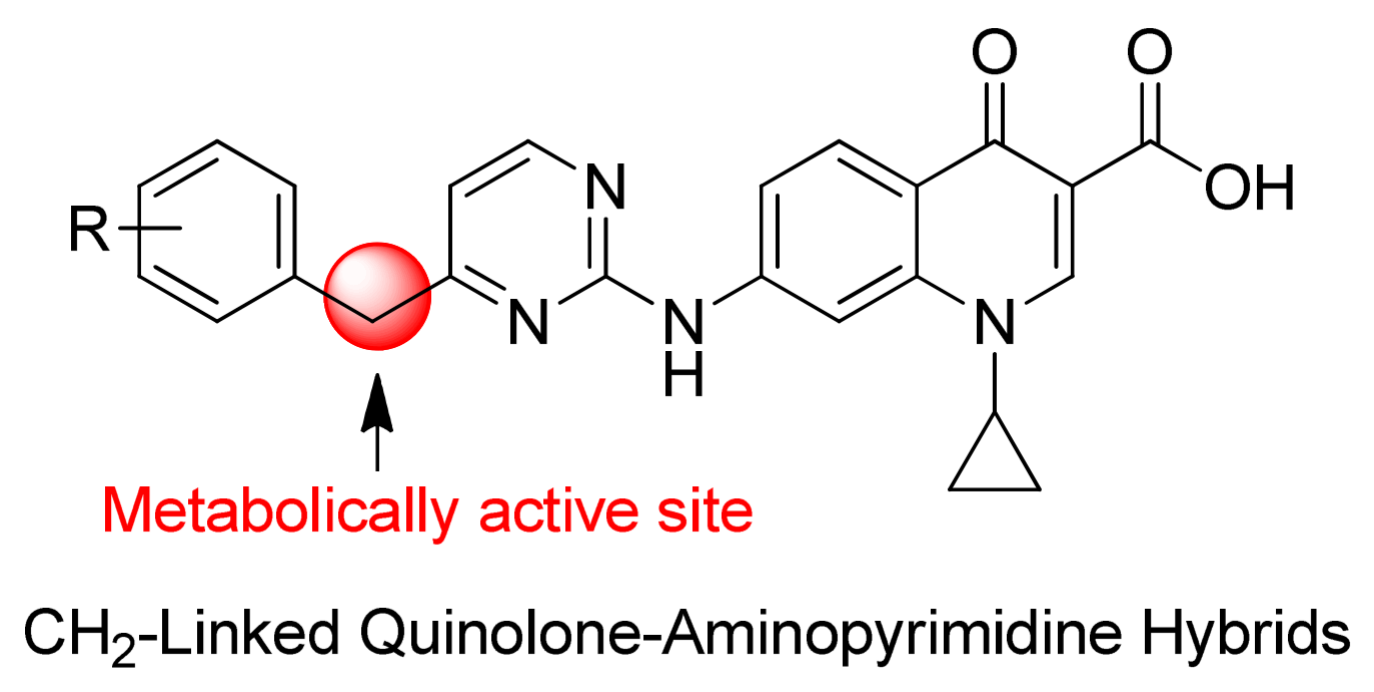
The structure of CH_2_-linked desfluoroquinolone-aminopyrimidine hybrids.

**Figure 2:**
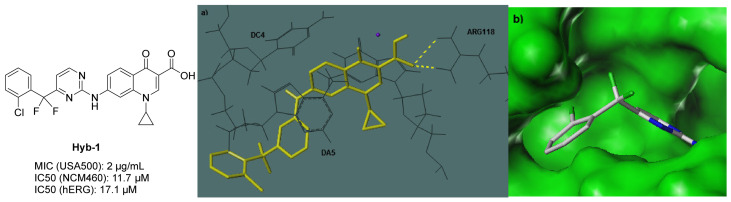
The structure of **Hyb-1** and its predicted binding mode. (**a**) Predicted binding mode with bacterial topoisomerase IV/DNA complex, (**b**) the binding pocket where the difluorinated benzyl group is located.

Building on this urgent need, in our previous work we employed a molecular hybridization strategy [18,19,20,21] to combine the structural motifs of quinolones and aminopyrimidines, aiming to develop novel anti-MRSA agents [22,23,24]. This effort led to the design and synthesis of a series of CH_2_-linked desfluoroquinolone–aminopyrimidine hybrids (Figure 1) [23]. Further optimization at the benzyl position afforded the CF_2_-linked derivative **Hyb-1** (Figure 2), which demonstrated a high barrier to resistance development, with an unchanged MIC of 2 μg/mL against MRSA USA500 after 14 serial passages [24]. In addition, **Hyb-1** retained potent activity against fluoroquinolone-resistant MRSA strains despite its structural similarity to conventional fluoroquinolones. However, **Hyb-1** exhibited measurable cytotoxicity toward NCM460 cells (IC_50_ = 11.7 μM) and moderate hERG inhibitory activity (IC_50_ = 17.1 μM), indicating that further structural optimization is required to improve its safety profile [24].

**Figure 3:**
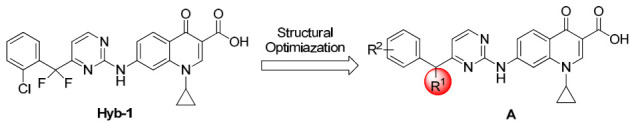
Design of target compound **A**.

Molecular docking analysis indicated that the difluorobenzyl group of **Hyb-1** is positioned within a binding pocket of the target enzyme that contains considerable unoccupied space (Figure 2) [24]. On the basis of this finding, we reasoned that introducing carbon-chain substituents bearing diverse functional groups at the benzyl position of the CH_2_-linked quinolone–aminopyrimidine hybrids could more effectively occupy the available space in the active pocket and enhance interactions between the ligand and the enzyme. Such an optimization strategy was anticipated to improve target selectivity, enhance antibacterial activity, reduce cytotoxicity, and confer greater metabolic stability. Accordingly, target compounds **A** were designed as shown in Figure 3. Furthermore, our previous comprehensive structure–activity relationship (SAR) studies revealed that quinolone–aminopyrimidine hybrids bearing 2,4-difluoro, 3,4-difluoro, or 3,4-dimethyl substituents on the benzene ring exhibited excellent anti-MRSA activity. These substituents were therefore retained in the design of target compounds **A**.

**Scheme 1:**
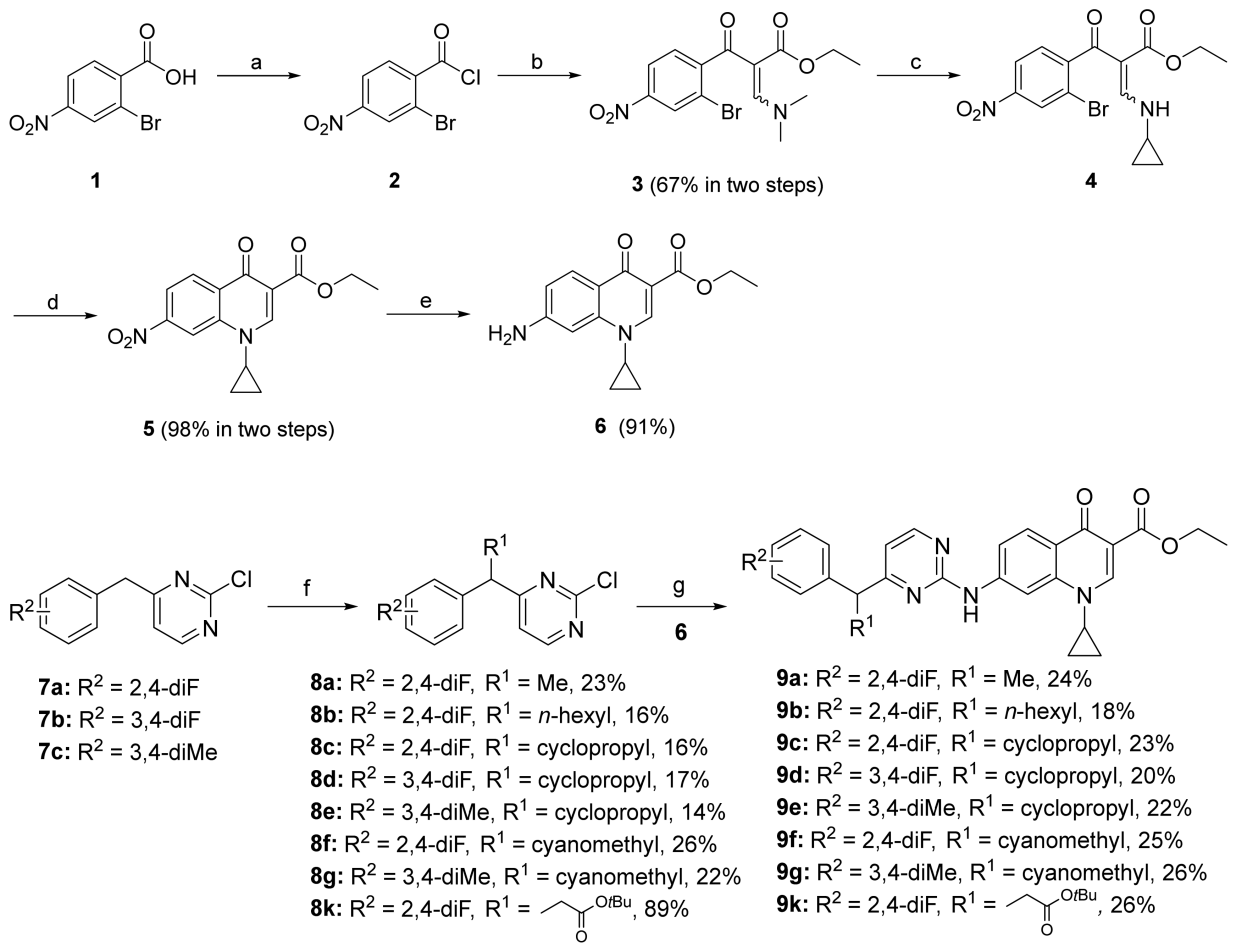

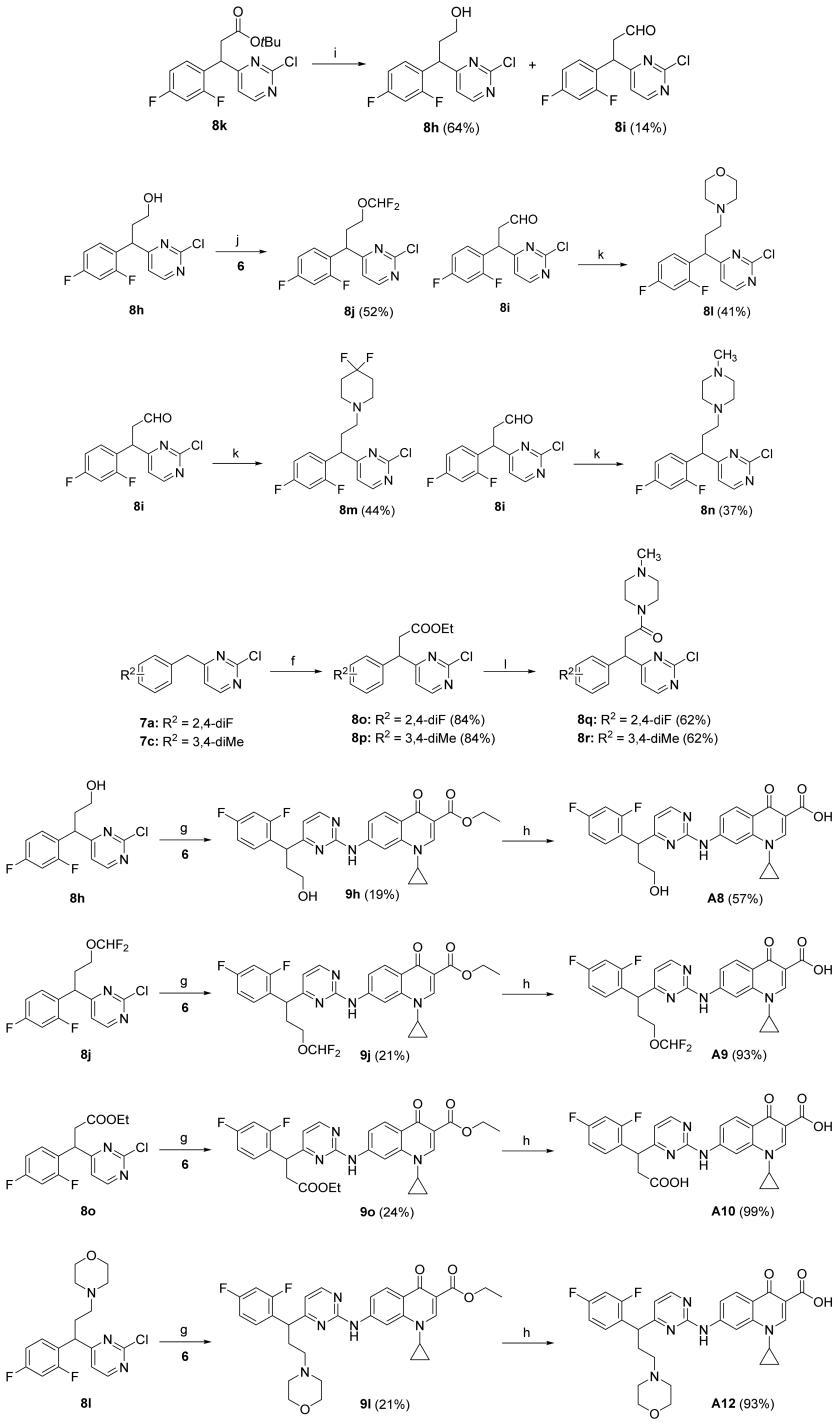

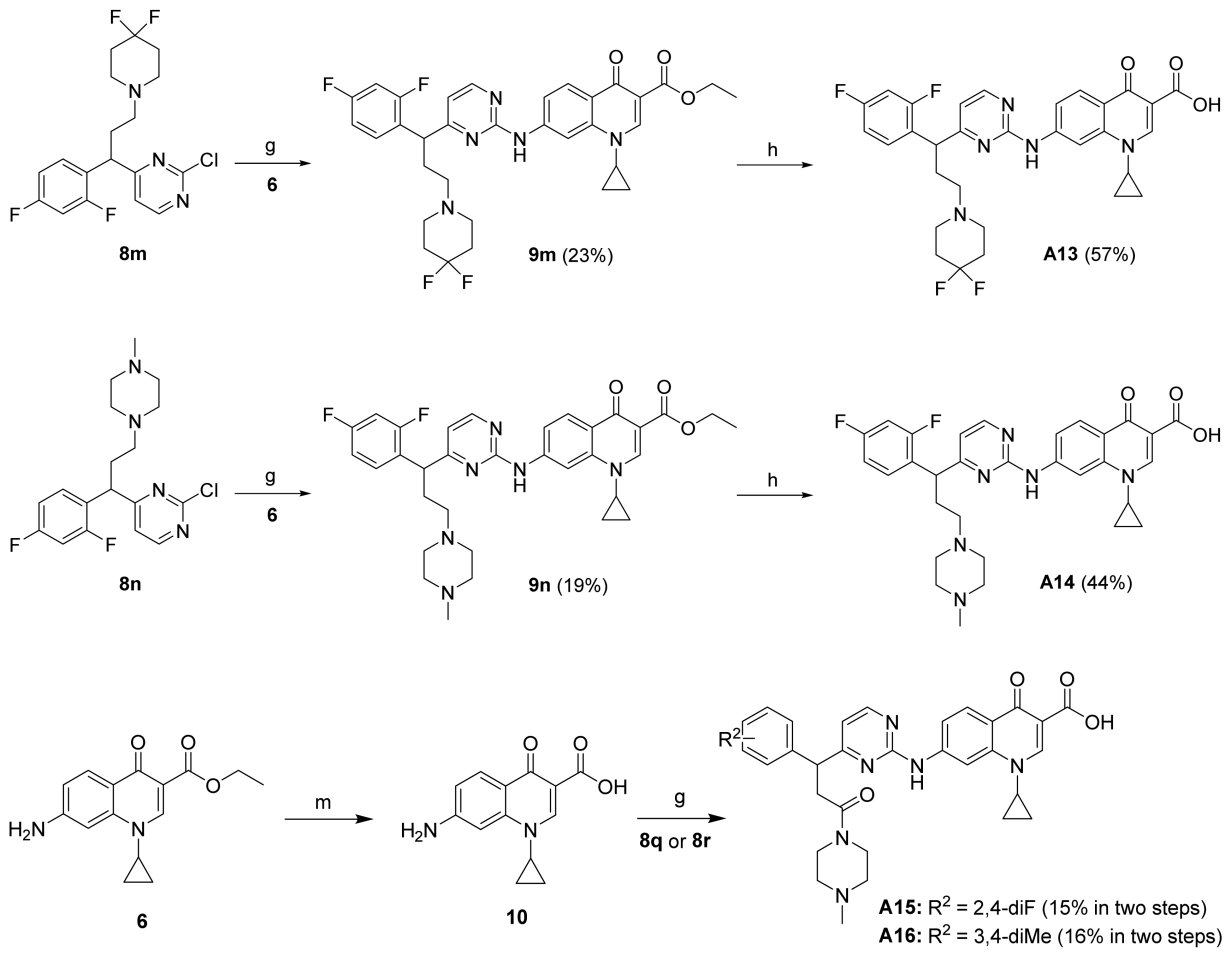
Synthesis of desfluoroquinolone-aminopyrimidine hybrids **A**.^a a^Reagents and conditions: (a) SOCl_2_, DMF, toluene, 65 °C, 3.5 h; (b) ethyl 3-(dimethylamino)acrylate, Et_3_N, toluene, reflux, overnight; (c) cyclopropylamine, THF, r.t., 1 h; (d) K_2_CO_3_, DMF, 85 °C, 8 h; (e) 4,4′-bipyridine, B_2_(OH)_4_, DMF, 0 °C to rt, 15 min; (f) (i) NaHMDS, THF, −78 °C, 10 min, (ii) R^1^X, r.t., 1–8 h; (g) BINAP, Pd(OAc)_2_, K_2_CO_3_, toluene, reflux, 8–12 h; (h) 5 M LiOH, THF, 50 °C, 3–6 h; (i) DIBAL-H, THF, −78 °C, 30 min; then r.t., 8 h; (j) CuI, 2,2-difluoro-2-(fluorosulfonyl)acetic acid, acetonitrile, 50 °C, 1 h; (k) acetic acid, sodium cyanoborohydride, 1,2-dichloroethane, r.t., 3 h; (l) (i) 5 N NaOH, THF, r.t., 3 h, (ii) *N*-methylpiperazine, EDCI·HCl, HOBt, Et_3_N, DCM, r.t., 8 h; (m) 5 N NaOH, EtOH/H_2_O = 1/1, 50 °C, 3.0 h.

The synthetic route to the newly designed quinolone–aminopyrimidine hybrids **A** is outlined in Scheme 1. 2-Bromo-4-nitrobenzoic acid (**1**) was treated with thionyl chloride in the presence of a catalytic amount of DMF to afford the corresponding acyl chloride **2**. Subsequent condensation of **2** with *N,N*-dimethylformamide dimethyl acetal (DMFDMA), followed by nucleophilic substitution with cyclopropylamine, gave acrylate **4**. Cyclization of **4** in the presence of K_2_CO_3_ furnished 7-nitroquinolone **5**, which was then reduced with tetrahydroxydiboron to provide 7-aminoquinolone **6**.

Intermediate **7a–c** was prepared via a Negishi cross-coupling reaction of various benzyl bromides with 2,4-dichloropyrimidine according to a method reported in our previous study [23]. Deprotonation of **7a–c** with NaHMDS, followed by alkylation with the corresponding alkyl halides afforded intermediate **8a–g** and **8k**. Reduction of **8k** with DIBAL-H afforded **8h** and **8i**. Treatment of **8h** with 2-fluorosulfonyl difluoroacetic acid in the presence of CuI gave **8j**. Intermediate **8i** underwent reductive amination with morpholine, 4,4-difluoropiperidine, or *N*-methylpiperazine in the presence of sodium cyanoborohydride to afford intermediates **8l–8n**. In parallel, deprotonation of **7a** or **7c,** followed by alkylation with ethyl bromoacetate, generated **8o** or **8p**, respectively; subsequent condensation with *N*-methylpiperazine furnished **8q** and **8r**.

Finally, the target hybrids **A1–16** were synthesized via Buchwald–Hartwig amination of 7-aminoquinolone **6** with the appropriate intermediate **8**, followed by hydrolysis.

## 2. Results and Discussion

### 2.1. In Vitro Antibacterial Activities

#### 2.1.1. Antibacterial Activities Against MRSA ATCC33591 and *E. coli* ATCC25922

The newly synthesized desfluoroquinolone–aminopyrimidine hybrids **A** were evaluated for antibacterial activity against MRSA ATCC 33591 and *E. coli* ATCC 25922, using ciprofloxacin, vancomycin, and **Hyb-1** as reference compounds.

As shown in Table 1, several compounds displayed moderate to potent anti-MRSA activity, with MIC values of 2–8 μg/mL. Among them, **A3** and **A5** were the most active, both showing an MIC value of 2 μg/mL, comparable to those of vancomycin and **Hyb-1**.

SAR analysis indicated that alkyl substitution at the benzyl position was tolerated, and alkyl chain length had only a minor effect on activity. **A1** (methyl) and **A2** (*n*-hexyl) exhibited MIC values of 4 and 8 μg/mL, respectively, whereas **A3** bearing a 1,2-ethylene substituent was more potent (MIC = 2 μg/mL). Further optimization of the terminal phenyl ring showed that the 3,4-difluoro analogue **A4** was less active (MIC = 8 μg/mL), while the 2,4-difluoro analogue **A3** and the 3,4-dimethyl analogue **A5** maintained potent activity (MIC = 2 μg/mL).

The terminal substituent on the benzyl side chain had a marked influence on anti-MRSA activity. Weakly polar groups such as cyano (**A6** and **A7**) and difluoromethoxy (**A9**) afforded moderate activity (MIC = 4–8 μg/mL), whereas strongly polar groups such as hydroxyl (**A8**) and carboxyl (**A10**) significantly impaired activity. Esterification of the carboxyl group in **A10** restored potency, giving the corresponding *tert*-butyl ester with an MIC value of 4 μg/mL. In contrast, most nitrogen-containing six-membered heterocyclic analogues were inactive, with the exception of the difluorinated piperidine derivative **A13** (MIC = 4 μg/mL). All compounds in this series were inactive against *E. coli* ATCC 25922, likely due to poor penetration through the Gram-negative cell envelope.

#### 2.1.2. Antibacterial Activities Against MRSA Strains USA500 and Mu50

Based on the activity results against the MRSA ATCC33591 strain, we selected several of these compounds for further testing of their inhibitory activity against MRSA USA500 and Mu50. MRSA USA500 is a community-acquired MRSA strain, and Mu50 is a fluoroquinolone-resistant/vancomycin-intermediate MRSA isolate. Ciprofloxacin and vancomycin were used as references following the same procedure.

On the basis of the antibacterial results against MRSA ATCC 33591, several representative compounds were selected for further evaluation against MRSA USA500 and Mu50. USA500 is a community-acquired MRSA strain, while Mu50 is a fluoroquinolone-resistant, vancomycin-intermediate MRSA isolate. Ciprofloxacin and vancomycin were used as reference compounds under identical assay conditions.

As summarized in Table 2, all tested hybrids displayed moderate to good activity against both USA500 and Mu50, with MIC values in the range of 1.25–5 μg/mL. Importantly, their antibacterial activities against Mu50 were largely maintained and were comparable to those observed against USA500, indicating no significant cross-resistance. By contrast, ciprofloxacin exhibited more than a 16-fold loss of activity against Mu50 relative to USA500, while vancomycin showed a 4-fold reduction. These findings further demonstrate that installation of an aminopyrimidine side chain at the C-7 position of the quinolone core effectively mitigates cross-resistance to currently used fluoroquinolones, in agreement with our previous studies. Among the tested compounds, **A3** and **A5** were the most active, with MIC values of 1.25–2.5 μg/mL against both strains.

### 2.2. Mammalian Cytotoxicity

On the basis of their overall antibacterial activities against MRSA ATCC 33591, USA500, and Mu50, the two most potent hybrids, **A3** and **A5**, were selected for cytotoxicity evaluation. Cytotoxicity was determined in the human non-small cell lung cancer cell line A549 and the human normal colonic epithelial cell line NCM460, with doxorubicin as the reference compound.

**Figure 4:**
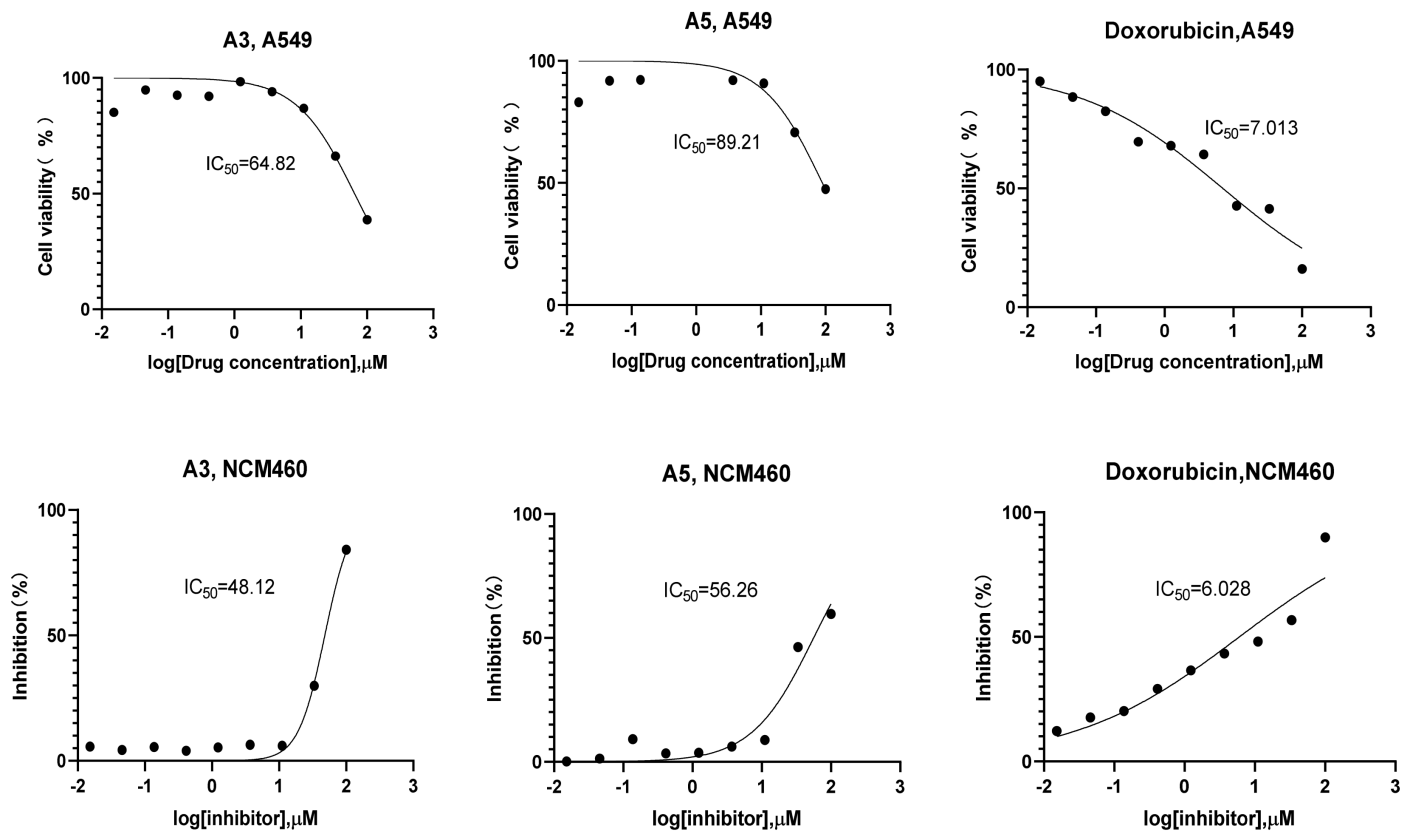
Cytotoxicity test results of the target compounds on A549 and NCM460 cells.

As illustrated in Figure 4, both **A3** and **A5** showed minimal cytotoxicity toward A549 and NCM460 cells. **A3** exhibited IC_50_ values of 64.8 μM and 48.1 μM against A549 and NCM460 cells, respectively, whereas **A5** showed IC_50_ values of 89.2 μM and 56.3 μM, respectively. In comparison, the previously reported hybrid **Hyb-1**, bearing a difluorinated substituent at the benzyl position, showed pronounced cytotoxicity toward NCM460 cells (IC_50_ = 11.7 μM). These findings indicate that replacement of the benzyl difluoro substituent with a 1,2-ethylene group is beneficial for reducing cytotoxicity in this series.

### 2.3. Propensity to Induce Bacterial Resistance

Due to the rapid global spread of microbial resistance, bacteria often develop resistance to antibiotics within a short period of time, posing great challenges to the development of new antibiotics. Therefore, whether a hybrid is prone to inducing drug resistance has become an important indicator for evaluating its druggability. Using USA500 as the test strain, we evaluated the bacterial induced drug resistance of the selected hybrids **A3** and **A5**, with ciprofloxacin as the positive control.

**Figure 5:**
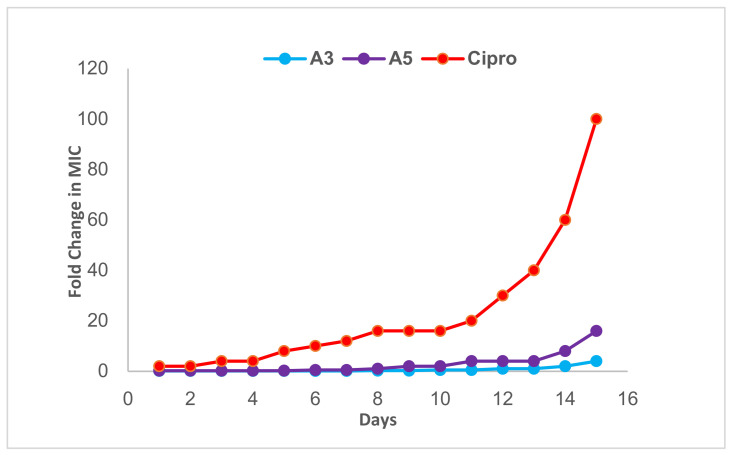
Resistance development in MRSA USA500: comparison of fold increases in MIC values for **A3**, **A5**, and ciprofloxacin during serial passage.

As shown in Figure 5, the MIC values of hybrids **A3** and **A5** against USA500 strain after 15 days of induced culture increased by 4-fold and 16-fold, respectively. In contrast, the MIC value of ciprofloxacin against USA500 strain after 15 days of induced culture increased by 100-fold. It can be concluded that, compared with ciprofloxacin, hybrids **A3** and **A5** have a lower possibility of inducing drug resistance and possess potential research value.

### 2.4. Liver Microsome Lability

The metabolic stability of hybrids **A3** and **A5** was evaluated in human liver microsomes (HLMs) and rat liver microsomes (RLMs), using midazolam as the reference compound.

As shown in Table 3, both compounds bearing a 1,2-ethylene group at the benzyl position exhibited good microsomal stability. **A5** showed favorable metabolic stability, with half-lives of 68.8 min in HLMs and 25.5 min in RLMs. In contrast, **A3** was considerably more stable, displaying markedly prolonged half-lives of 9863 min in HLMs and 983 min in RLMs. While this high stability may be advantageous for maintaining systemic exposure, the extremely slow metabolic clearance of **A3** may also indicate a potential liability for in vivo accumulation.

### 2.5. Molecular Simulation

To gain insight into the binding mode of the synthesized hybrids toward the target enzyme, molecular docking studies of representative compounds **A3**, **A5** and the reference drug ciprofloxacin were carried out using the bacterial topoisomerase IV–DNA complex (PDB ID: 4KPF) in Sybyl-X 2.0. The total scores of **A3**, **A5** and ciprofloxacin were 9.83, 9.88 and 10.05, respectively.

**Figure 6:**
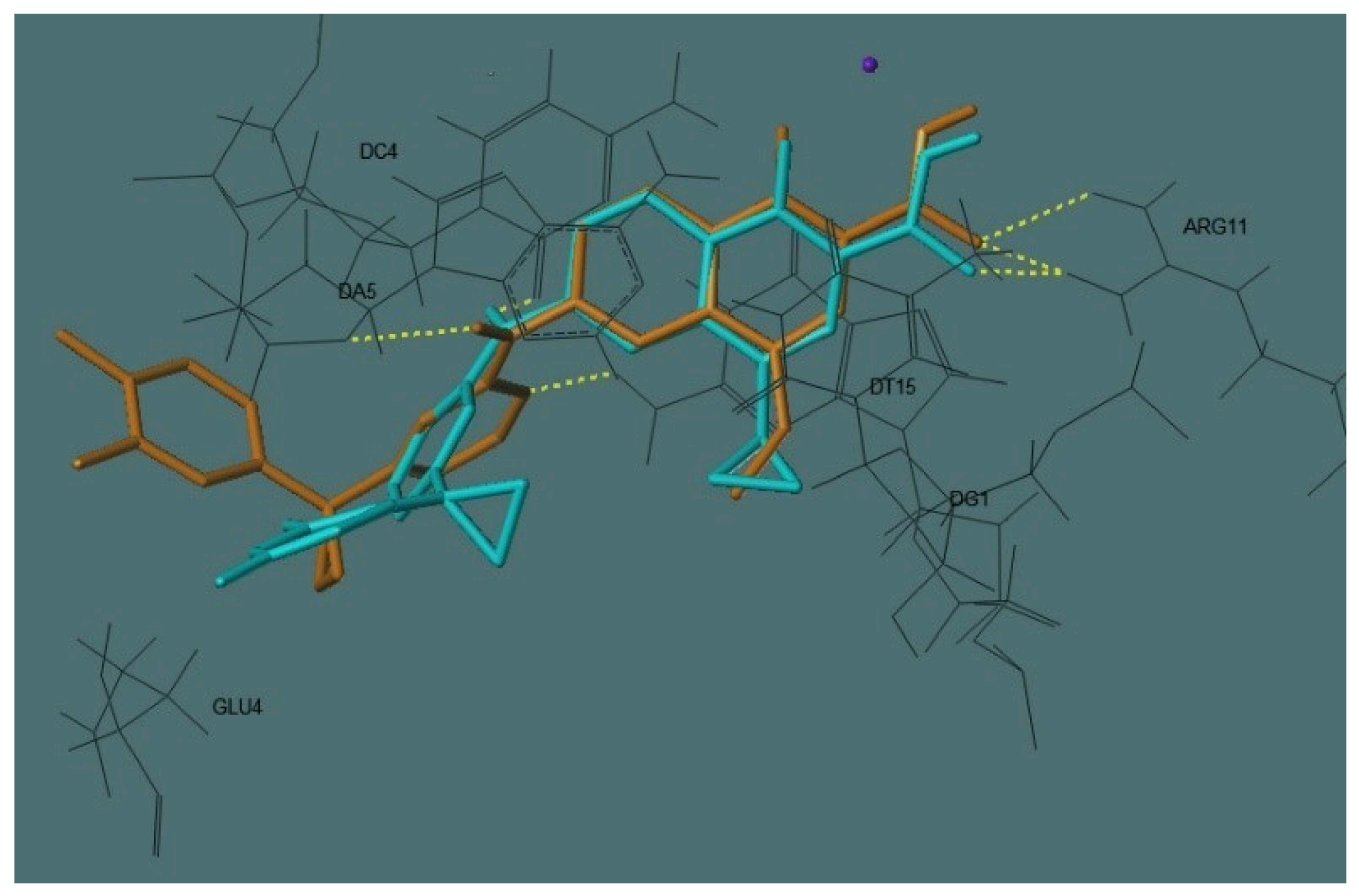
Predicted binding mode of **A3** (cyan) and **A5** (orange) with topoisomerase IV/DNA complex (derived from PDB code 4KPF) for comparison. Hydrogen bonds are indicated with dashed lines in yellow.

As shown in Figure 6, **A3** (cyan) and **A5** (orange) adopted similar binding conformations within the active site. In both cases, the C-3 carboxyl group and C-4 carbonyl group of the quinolone scaffold coordinated with Mg^2+^, while the C-3 carboxyl group also formed a hydrogen bond with Arg11. However, the two hybrids differed in the interaction modes of their aminopyrimidine substituents. For **A3**, the N-H group of the C-7 side chain formed a hydrogen bond with base DC4. In contrast, for **A5**, the N-H group at the C-7 position formed a hydrogen bond with base DA5, and the pyrimidine nitrogen additionally interacted with base DG1 through hydrogen bonding. These results indicate that the C-7 aminopyrimidine side chain is capable of establishing direct hydrogen-bonding interactions with DNA, which may lessen dependence on the Mg^2+^-water bridge that is important for classical fluoroquinolone binding. This binding feature may help explain the retained activity of these hybrids against ciprofloxacin-resistant strains. In addition, the involvement of highly conserved DNA components in ligand recognition may be associated with a relatively high barrier to resistance development.

## 3. Experimental Section

### 3.1. Chemistry

^1^H NMR and ^13^C NMR spectra were recorded on a Bruker Advance Neo 400 MHz spectrometer. Chemical shifts are reported in *δ* (ppm) units relative to the internal standard tetramethylsilane (TMS). HRMS data were obtained using electrospray ionization (ESI) techniques on a Water G2-xs tof instrument (Waters Corporation, Milford, MA, USA). The NMR and HRMS spectra of target compounds **A1–16** are provided in the Supplementary Materials (SI). Melting points were measured with a WRS-2 digital melting-point apparatus (Shanghai Shenguang Instrument Co., Ltd., Shanghai, China) and are uncorrected. All chemicals and solvents used were of reagent grade and were purified and dried by standard methods before use. All the reactions were monitored by thin layer chromatography (TLC) on precoated silica gel G plates at 254 nm under a UV lamp using dichloromethane/methanol or ethyl acetate/hexane as eluent. Column chromatography separations were obtained on silica gel (300–400 mesh) using dichloromethane and methanol as eluents. Analysis of sample purity was performed on an Agilent 1260 series HPLC system with Agilent Eclipse Plus C18 (4.6 mm × 250 mm, 5 μm) (Agilent Technologies, Santa Clara, CA, USA). HPLC conditions were as follows: solvent A = water (0.1% TFA), solvent B = MeCN; Method: 80% B within 6 min; fow rate = 1.2 mL/min. Purity was determined by the absorbance at 254 nm, and all tested compounds exhibited a purity of > 95%. The HPLC spectra of compounds **A3** and **A5** are included in the SI.

#### 3.1.1. Procedure for the Synthesis of 2-Bromo-4-Nitrobenzoyl Chloride (**2**)

To a solution of 2-bromo-4-nitrobenzoic acid (7.38 g, 30.0 mmol) in toluene (30.0 mL) was added thionyl chloride (12.49 g, 105.0 mmol) dropwise, followed by a few drops of DMF. The reaction mixture was heated at 65 °C for 3.5 h. After cooling to room temperature, the mixture was concentrated under reduced pressure to afford intermediate **2**, which was used directly in the next step without further purification.

#### 3.1.2. Procedure for the Synthesis of Ethyl 2-(2-Bromo-4-Nitrobenzoyl)-3-(Dimethylamino)acrylate (**3**)

Intermediate **2** was added dropwise to a solution of triethylamine (10.62 g, 105.0 mmol) and ethyl 3-(dimethylamino)acrylate (4.29 g, 30.0 mmol) in toluene (30.0 mL) at 0 °C. The reaction mixture was then heated at reflux overnight. After cooling to room temperature, the mixture was filtered, and the filter cake was washed with ethyl acetate. The filtrate was concentrated under reduced pressure, and the crude product was purified by column chromatography (petroleum ether/ethyl acetate = 4:1 to 2:1) to afford intermediate **3** as a yellow oil (6.17 g, 67% yield). ^1^H NMR (400 MHz, Chloroform-*d*) *δ* 8.43 (d, *J* = 2.3 Hz, 1H), 8.19 (dd, *J* = 8.4, 2.2 Hz, 1H), 7.98 (s, 1H), 7.46 (d, *J* = 8.2 Hz, 1H), 3.94 (q, *J* = 7.1 Hz, 2H), 3.42 (s, 3H), 3.09 (s, 3H), 0.91 (t, *J* = 7.1 Hz, 3H).

#### 3.1.3. Procedure for the Synthesis of Ethyl 1-Cyclopropyl-7-Nitro-4-Oxo-1,4-Dihydroquinoline-3-Carboxylate (**5**)

To a solution of intermediate **3** (7.46 g, 20.1 mmol) in THF (20.0 mL) was added cyclopropylamine (1.49 g, 26.1 mmol) dropwise, and the reaction mixture was stirred at room temperature for 1 h. The solvent was then removed under reduced pressure to afford crude intermediate **4**, which was used directly in the next step.

Intermediate **4** was dissolved in DMF (15.0 mL), and anhydrous K_2_CO_3_ (6.94 g, 50.2 mmol) was added. The reaction mixture was stirred at 85 °C for 8 h and then cooled to room temperature. The mixture was poured into ice-water, stirred, and filtered. The filter cake was washed with water and dried to afford 7-nitroquinolone **5** as a yellow solid (5.95 g, 98% yield). ^1^H NMR (400 MHz, Chloroform-*d*) *δ* 8.48 (s, 1H), 8.24 (d, *J* = 2.3 Hz, 1H), 8.13 (d, *J* = 8.4 Hz, 1H), 7.68 (dd, *J* = 8.4, 2.3 Hz, 1H), 4.23 (q, *J* = 7.1 Hz, 2H), 3.70–3.65 (m, 1H), 1.31–1.24 (m, 5H), 1.13–1.09 (m, 2H).

#### 3.1.4. Procedure for the Synthesis of Ethyl 7-Amino-1-Cyclopropyl-4-Oxo-1,4-Dihydroquinoline-3-Carboxylate (**6**)

To a solution of intermediate **5** (1.51 g, 5.0 mmol) and 4,4′-bipyridine (39.0 mg, 5 mol%) in DMF (15 mL) was added tetrahydroxydiboron (1.79 g, 20.0 mmol) portionwise at 0 °C. The reaction mixture was allowed to warm to room temperature and stirred for 15 min. The mixture was then poured into ice-water, stirred, and filtered. The filter cake was washed with water and dried to afford 7-aminoquinolone **6** as a yellow solid (1.24 g, 91% yield). ^1^H NMR (400 MHz, DMSO-*d*_6_) *δ* 8.30 (s, 1H), 7.75 (d, *J* = 8.4 Hz, 1H), 7.01 (s, 1H), 6.70 (d, *J* = 8.4 Hz, 1H), 6.15 (s, 2H), 4.19 (q, *J* = 7.1 Hz, 2H), 3.48–3.44 (m, 1H), 1.27 (t, *J* = 7.1 Hz, 3H), 1.21–1.16 (m, 2H), 1.08–1.01 (m, 2H).

#### 3.1.5. Procedure for the Synthesis of Benzyl-Substituted Arylpyrimidines (**8a–g**, **8k**, **8o**, **8p**)

Under a nitrogen atmosphere, a 2.0 M solution of sodium bis(trimethylsilyl)amide in THF (5.0 mL, 10.0 mmol) was added dropwise to a solution of **7** (10.0 mmol) in anhydrous THF (10 mL) at −78 °C. The reaction mixture was stirred at −78 °C for 10 min, and then a solution of the appropriate halide (10.0 mmol) in anhydrous THF (10.0 mL) was added dropwise. After completion of the addition, the mixture was allowed to warm to room temperature and stirred for 1–8 h. The reaction was quenched with saturated aqueous NaHCO_3_ (15 mL), and the mixture was extracted with ethyl acetate (15 mL × 3). The combined organic layers were washed with water, dried over anhydrous Na_2_SO_4_, and concentrated under reduced pressure. The residue was purified by column chromatography (petroleum ether/ethyl acetate = 10:1 to 5:1) to afford intermediate **8a–g**, **8k**, **8o**, **8p**.

**2-Chloro-4-(1-(2,4-difluorophenyl)ethyl)pyrimidine (8a):** Yield 23%; Yellow oil; ^1^H NMR (400 MHz, Chloroform-*d*) *δ* 8.49 (d, *J* = 5.0 Hz, 1H), 7.16–7.09 (m, 2H), 7.05–7.00 (m, 2H), 4.18 (q, *J* = 7.2 Hz, 1H), 1.68 (d, *J* = 7.2 Hz, 3H).
**2-Chloro-4-(1-(2,4-difluorophenyl)heptyl)pyrimidine (8b):** Yield 16%; Colorless oil; ^1^H NMR (400 MHz, Chloroform-*d*) *δ* 8.50 (d, *J* = 5.0 Hz, 1H), 7.44–7.38 (m, 1H), 7.13 (d, *J* = 5.0 Hz, 1H), 6.91–6.85 (m, 1H), 6.84–6.78 (m, 1H), 4.31 (t, *J* = 7.2 Hz, 1H), 2.28–2.19 (m, 1H), 2.06–1.99 (m, 1H), 1.37–1.23 (m, 12H), 0.88 (t, *J* = 6.8 Hz, 3H).
**2-Chloro-4-(1-(2,4-difluorophenyl)cyclopropyl)pyrimidine (8c)**: Yield 16%;White solid; ^1^H NMR (400 MHz, Chloroform-d) *δ* 8.29 (d, *J* = 5.2 Hz, 1H), 7.38–7.32 (m, 1H), 6.96–6.80 (m, 2H), 6.69 (d, *J* = 5.2 Hz, 2H), 1.93–1.90 (m, 2H), 1.44–1.42 (m, 2H).
**2-Chloro-4-(1-(3,4-difluorophenyl)cyclopropyl)pyrimidine (8d):** Yield 17%; White solid; ^1^H NMR (400 MHz, Chloroform-*d*) *δ* 8.31 (d, *J* = 5.2 Hz, 1H), 7.25–7.18 (m, 2H), 7.15–7.11 (m, 1H), 6.69 (d, *J* = 5.2 Hz, 1H), 1.89–1.87 (m, 2H), 1.47–1.44 (m, 2H).
**2-Chloro-4-(1-(3,4-dimethylphenyl)cyclopropyl)pyrimidine (8e):** Yield 14%; White solid; ^1^H NMR (400 MHz, Chloroform-*d*) *δ* 8.24 (d, *J* = 5.0 Hz, 1H), 7.19–7.10 (m, 3H), 6.74 (d, *J* = 5.0 Hz, 1H), 2.31 (s, 3H), 2.30 (s, 3H), 1.86–1.81 (m, 2H), 1.48–1.44 (m, 2H).
**3-(2-chloropyrimidin-4-yl)-3-(2,4-difluorophenyl)propanenitrile (8f):** Yield 26%; Orange oil; ^1^H NMR (400 MHz, Chloroform-*d*) *δ* 8.53 (d, *J* = 5.0 Hz, 1H), 7.39–7.33 (m, 1H), 7.15 (d, *J* = 5.0 Hz, 1H), 6.95–6.84 (m, 2H), 4.50 (t, *J* = 7.2 Hz, 1H), 2.77–2.67 (m, 1H), 2.45–2.34 (m, 3H).
**4-(2-Chloropyrimidin-4-yl)-4-(3,4-dimethylphenyl)butanenitrile (8g):** Yield 22%; Orange oil; ^1^H NMR (400 MHz, Chloroform-*d*) *δ* 8.40 (d, *J* = 5.0 Hz, 1H), 7.16 (d, *J* = 5.0 Hz, 1H), 7.09–7.01 (m, 3H), 4.10 (t, *J* = 7.2 Hz, 1H), 2.73–2.62 (m, 1H), 2.43–2.34 (m, 3H), 2.31 (s, 3H), 2.30 (s, 3H).
***tert*-Butyl 3-(2-chloropyrimidin-4-yl)-3-(2,4-difluorophenyl) propanoate (8k):** Yield 89%; Yellow oil; ^1^H NMR (400 MHz, Chloroform-*d*) *δ* 8.47 (d, *J* = 5.0 Hz, 1H), 7.31–7.22 (m, 1H), 7.11 (d, *J* = 5.2 Hz, 1H), 6.88–6.78 (m, 2H), 4.81 (t, *J* = 7.4 Hz, 1H), 3.37–3.31 (m, 1H), 2.87–2.81 (m, 1H), 1.34 (s, 9H).
**Ethyl 3-(2-chloropyrimidin-4-yl)-3-(2,4-difluorophenyl)propanoate (8o):** Yield 84%; Yellow oil; ^1^H NMR (400 MHz, Chloroform-*d*) *δ* 8.49 (d, *J* = 5.0 Hz, 1H), 7.35–7.29 (m, 1H), 7.14 (d, *J* = 5.0 Hz, 1H), 6.90–6.78 (m, 2H), 4.90–4.86 (m, 3H), 3.51–3.44 (m, 1H), 2.96–2.90 (m, 1H), 1.20 (t, *J* = 7.1 Hz, 3H).
**Ethyl 3-(2-chloropyrimidin-4-yl)-3-(3,4-dimethylphenyl)propanoate (8p):** Yield 84%; Yellow oil; ^1^H NMR (400 MHz, Chloroform-*d*) *δ* 8.44 (d, *J* = 5.0 Hz, 1H), 7.11–7.08 (m, 2H), 7.04–7.01 (m, 2H), 4.52 (m, 1H), 4.15 (q, *J* = 7.1 Hz, 2H), 3.50–3.44 (m, 1H), 2.93–2.87 (m, 1H), 2.25 (s, 3H), 2.24 (s, 3H), 1.28 (t, *J* = 7.1 Hz, 3H).

#### 3.1.6. Procedure for the Synthesis of **8h** and **8i**

To a solution of intermediate **8k** (5.32 g, 15.0 mmol) in anhydrous THF (15.0 mL) under a nitrogen atmosphere at −78 °C was added 1.0 M DIBAL-H in *n*-hexane (33.0 mL, 33.0 mmol) dropwise. The reaction mixture was stirred at −78 °C for 30 min and then allowed to warm to room temperature and stirred for an additional 8 h. 1.5 M Aqueous potassium sodium tartrate tetrahydrate was added slowly, and the mixture was stirred for 1 h. The reaction mixture was extracted with ethyl acetate, and the combined organic layers were washed with water, dried over anhydrous Na_2_SO_4_, and concentrated under reduced pressure. The residue was purified by column chromatography (petroleum ether/ethyl acetate = 3:1 to 1:1) to afford intermediates **8h** and **8i**.

**3-(2-Chloropyrimidin-4-yl)-3-(2,4-difluorophenyl)propan-1-ol (8h):** Yield 64%; Yellow oil; ^1^H NMR (400 MHz, Chloroform-*d*) *δ* 8.50 (d, *J* = 5.0 Hz, 1H), 7.44–7.38 (m, 1H), 7.15 (d, *J* = 5.0 Hz, 1H), 6.93–6.89 (m, 1H), 6.85–6.80 (m, 1H), 4.62 (t, *J* = 7.6 Hz, 1H), 3.65 (t, *J* = 6.4 Hz, 2H), 2.60–2.51 (m, 1H), 2.32–2.23 (m, 1H).
**3-(2-Chloropyrimidin-4-yl)-3-(2,4-difluorophenyl)propanal (8i):** Yield 14%; colorless oil; 9.84 (s, 1H), 8.49 (d, *J* = 5.0 Hz, 1H), 7.31–7.25 (m, 1H), 7.14 (d, *J* = 5.0 Hz, 1H), 6.93–6.78 (m, 2H), 4.96 (dd, *J* = 9.7, 4.6 Hz, 1H), 3.81–3.74 (m, 1H), 3.06–3.00 (m, 1H).

#### 3.1.7. Procedure for the Synthesis of **8j**

To a solution of intermediate **8h** (569 mg, 2.0 mmol) in acetonitrile (5.0 mL) were added CuI (0.08 g, 10 mol%) and 2,2-difluoro-2-(fluorosulfonyl)acetic acid (0.36 g, 2.0 mmol). The reaction mixture was heated at 50 °C for 1 h. Saturated aqueous NH_4_Cl was added, and the mixture was extracted with ethyl acetate. The combined organic layers were washed with water, dried over anhydrous Na_2_SO_4_, and concentrated under reduced pressure. The residue was purified by column chromatography (petroleum ether/ethyl acetate = 5:1) to afford intermediate **8j**.

**2-Chloro-4-(3-(difluoromethoxy)-1-(2,4-difluorophenyl)propyl) pyrimidine (8j):** Yield 52%; Yellow oil; ^1^H NMR (400 MHz, Chloroform-*d*) *δ* 8.52 (d, *J* = 5.2 Hz, 1H), 7.44–7.39 (m, 1H), 7.16 (d, *J* = 5.2 Hz, 1H), 6.92 (dt, *J* = 7.7, 2.7 Hz, 1H), 6.86–6.81 (m, 1H), 6.18 (t, *J* = 74.5 Hz, 1H), 4.57 (t, *J* = 7.6 Hz, 1H), 3.88–3.78 (m, 2H), 2.74–2.65 (m, 1H), 2.41–2.32 (m, 4H).

#### 3.1.8. Procedure for the Synthesis of **8l–n**

To a solution of intermediate **8i** (0.57 g, 2.0 mmol) in 1,2-dichloroethane (5.0 mL) were added two drops of acetic acid. After stirring for 5 min, the corresponding amine (3.0 mmol) was added dropwise, and the reaction mixture was stirred at room temperature for 1 h. Sodium cyanoborohydride (0.19 g, 3.0 mmol) was then added, and the mixture was stirred at room temperature for an additional 3 h. After completion of the reaction as monitored by TLC, saturated aqueous NaHCO_3_ was added, and the mixture was extracted with ethyl acetate. The combined organic layers were washed with water, dried over anhydrous Na_2_SO_4_, and concentrated under reduced pressure. The residue was purified by column chromatography (dichloromethane/methanol = 20:1) to afford intermediates **8l–n**.

**4-(3-(2-Chloropyrimidin-4-yl)-3-(2,4-difluorophenyl)propyl) morpholine (8l):** Yield 41%; White solid; ^1^H NMR (400 MHz, Chloroform-*d*) *δ* 8.48 (d, *J* = 5.0 Hz, 1H), 7.45–7.39 (m, 1H), 7.13 (d, *J* = 5.0 Hz, 1H), 6.90–6.77 (m, 2H), 4.48 (t, *J* = 7.6 Hz, 1H), 3.72–3.69 (m, 4H), 2.61–2.52 (m, 1H), 2.48–2.40 (m, 4H), 2.38 (t, *J* = 7.1 Hz), 2.26–2.18 (m, 1H).
**2-Chloro-4-(1-(2,4-difluorophenyl)-3-(4,4-difluoropiperidin-1-yl)propyl)pyrimidine (8m):** Yield 44%; White solid; ^1^H NMR (400 MHz, Chloroform-*d*) *δ* 8.49 (d, *J* = 5.0 Hz, 1H), 7.45–7.39 (m, 1H), 7.13 (d, *J* = 5.0 Hz, 1H), 6.93–6.88 (m, 1H), 6.82 (ddd, *J* = 10.3, 8.8, 2.6 Hz, 1H), 4.48 (t, *J* = 7.1 Hz, 1H), 2.57–2.45 (m, 5H), 2.36 (m, 1H), 1.34 (t, *J* = 6.4 Hz, 2H), 2.20–2.12 (m, 1H), 1.99–1.90 (m, 4H).
**2-Chloro-4-(1-(2,4-difluorophenyl)-3-(4-methylpiperazin-1-yl)propyl)pyrimidine (8n):** Yield 37%; Colorless oil; ^1^H NMR (400 MHz, Chloroform-*d*) *δ* 8.47 (d, *J* = 5.0 Hz, 1H), 7.46–7.40 (m, 1H), 7.13 (d, *J* = 5.0 Hz, 1H), 6.93–6.88 (m, 1H), 6.79 (ddd, *J* = 10.3, 8.7, 5.2 Hz, 1H), 4.44 (t, *J* = 7.2 Hz, 1H), 2.54–2.32 (m, 9H), 2.31 (t, *J* = 6.7 Hz, 2H) 2.27 (s, 3H), 2.19–2.12 (m, 1H).

#### 3.1.9. Procedure for the Synthesis of **8q** and **8r**

To a solution of intermediate **8o** (0.65 g, 2.0 mmol) in THF (8.0 mL) was added 10 N aqueous NaOH (10 mL). The reaction mixture was stirred at room temperature for 3 h, and the pH was then adjusted to 3 with 1.0 M aqueous HCl. The aqueous phase was extracted with dichloromethane (10 mL × 3), and the combined organic layers were dried over anhydrous Na_2_SO_4_ and concentrated under reduced pressure to afford the crude hydrolyzed product.

To a solution of the crude product in DCM (8.0 mL) were added triethylamine (0.26 g, 2.6 mmol) and *N*-methylpiperazine (0.26 g, 2.6 mmol). EDCI·HCl (0.50 g, 2.6 mmol) and HOBt (0.27 g, 2.6 mmol) were then added at 0 °C. The reaction mixture was allowed to warm to room temperature and stirred for 8 h. After completion of the reaction, saturated aqueous NH_4_Cl was added, and the mixture was extracted with ethyl acetate (8 mL × 3). The combined organic layers were washed with water, dried over anhydrous Na_2_SO_4_, and concentrated under reduced pressure. The residue was purified by column chromatography (dichloromethane/methanol = 20:1) to afford intermediate **8q**. Intermediate **8r** was prepared from intermediate **8p** following the same procedure.

**3-(2-chloropyrimidin-4-yl)-3-(2,4-difluorophenyl)-1-(4-methylpiperazin-1-yl)propan-1-one (8q):** Yield 62%; Colorless oil; ^1^H NMR (400 MHz, Chloroform-*d*) *δ* 8.47 (d, *J* = 5.0 Hz, 1H), 7.35–7.31 (m, 1H), 7.21 (d, *J* = 5.0 Hz, 1H), 6.88–6.79 (m, 2H), 5.05 (dd, *J* = 9.0, 4.7 Hz, 1H), 3.68–3.61 (m, 3H), 3.55–3.49 (m, 2H), 2.80–2.75 (m, 1H), 2.49–2.40 (m, 2H), 2.38–2.30 (m, 5H).
**3-(2-chloropyrimidin-4-yl)-3-(3,4-dimethylphenyl)-1-(4-methylpiperazin-1-yl)propan-1-one (8r):** Yield 62%; Yellow oil; ^1^H NMR (400 MHz, Chloroform-*d*) *δ* 8.40 (d, *J* = 5.0 Hz, 1H), 7.17 (d, *J* = 5.0 Hz, 1H), 7.09–7.02 (m, 3H), 4.66 (dd, *J* = 9.8, 4.7 Hz, 1H), 3.67–3.56 (m, 4H), 2.78–2.72 (m, 1H), 2.47–2.41 (m, 1H), 2.35–2.30 (m, 4H), 2.29 (s, 3H), 2.23 (s, 3H), 2.21 (s, 3H).

#### 3.1.10. Procedure for the Synthesis of the Intermediate **9**

To a Schlenk tube were added intermediate **6** (212 mg, 0.75 mmol), the appropriate intermediate **8** (0.50 mmol), anhydrous K_2_CO_3_ (104 mg, 0.75 mmol), BINAP (44 mg, 0.07 mmol), Pd(OAc)_2_ (12 mg, 0.05 mmol), and anhydrous toluene (8.0 mL). The mixture was refluxed under nitrogen for 8–12 h. After cooling to room temperature, the reaction mixture was filtered through Celite, and the filtrate was concentrated under reduced pressure. The residue was purified by silica gel column chromatography (CH_2_Cl_2_/MeOH = 30:1–10:1, *v*/*v*) to give intermediate **9**.

**Ethyl 1-cyclopropyl-7-((4-(1-(2,4-difluorophenyl)ethyl)pyrimidin-2-yl)amino)-4-oxo-1,4-dihydroq uinoline-3-carboxylate (9a):** Yield 24%; White solid; ^1^H NMR (400 MHz, Chloroform-*d*) *δ* 9.17 (s, 1H), 8.69 (d, *J* = 2.0 Hz, 1H), 8.58 (s, 1H), 8.43 (d, *J* = 9.0 Hz, 1H), 8.41 (d, *J* = 5.0 Hz, 1H), 7.64 (s, 1H), 7.45 (dd, *J* = 8.6, 2.0 Hz, 1H), 7.16–7.09 (m, 2H), 7.05–7.01 (m, 1H), 6.68 (d, *J* = 5.0 Hz, 1H), 4.41 (q, *J* = 7.1 Hz, 2H), 4.17–4.08 (m, 1H), 3.48–3.42 (m, 1H), 1.68 (d, *J* = 8.7 Hz, 3H), 1.42 (t, *J* = 7.1 Hz), 1.31–1.29 (m, 2H), 1.18–1.14 (m, 2H).
**Ethyl 1-cyclopropyl-7-((4-(1-(2,4-difluorophenyl)heptyl)pyrimidin-2-yl)amino)-4-oxo-1,4-dihydro quinoline-3-carboxylate (9b):** Yield 18%; White solid; ^1^H NMR (400 MHz, Chloroform-*d*) 8.67 (s, 1H), 8.58 (s, 1H), 8.42 (d, *J* = 8.9 Hz, 1H), 8.38 (d, *J* = 5.2 Hz, 1H), 7.73 (s, 1H), 7.46 (dd, *J* = 8.8, 2.2 Hz, 1H), 7.41–7.36 (m, 1H), 6.90–6.86 (m, 1H), 6.84–6.78 (m, 1H), 6.74 (d, *J* = 5.0 Hz, 1H), 4.40 (q, *J* = 7.1 Hz, 2H), 4.26 (t, *J* = 7.8 Hz, 1H), 3.48–3.46 (m, 1H), 2.27–2.20 (m, 1H), 2.03–1.98 (m, 1H), 1.42 (t, *J* = 7.1 Hz, 3H), 1.35–1.27 (m, 10H), 1.17–1.13 (m, 2H), 0.87 (t, *J* = 6.9 Hz, 3H).
**Ethyl 1-cyclopropyl-7-((4-(1-(2,4-difluorophenyl)cyclopropyl)pyrimidin-2-yl)amin o)-4-oxo-1,4-di hydroquinoline-3-carboxylate (9c):** Yield 23%; White solid; ^1^H NMR (400 MHz, Chloroform-*d*) δ 9.15 (s, 1H), 9.05 (s, 1H), 8.79 (d, *J* = 5.0 Hz, 1H), 8.51 (s, 1H), 8.30 (s, 1H), 7.94–7.88 (m, 1H), 7.27 (d, *J* = 5.0 Hz, 1H), 7.10–7.05 (m, 1H), 6.95–6.90 (m, 1H), 4.40 (t, *J* = 7.7 Hz, 2H), 3.08–3.02 (m, 1H), 1.43 (t, *J* = 7.7 Hz, 4H), 1.22–1.15 (m, 4H), 1.13–1.07 (m, 2H), 1.05–1.00 (m, 2H).
**Ethyl 1-cyclopropyl-7-((4-(1-(3,4-difluorophenyl)cyclopropyl)pyrimidin-2-yl)amin o)-4-oxo-1,4-di hydroquinoline-3-carboxylate (9d):** Yield 20%; White solid; ^1^H NMR (400 MHz, Chloroform-*d*) *δ* 10.13 (s, 1H), 8.56 (s, 1H), 8.44 (s, 1H), 8.37 (d, *J* = 5.2 Hz, 1H), 8.07 (d, *J* = 8.9 Hz, 1H), 8.33 (s, 1H), 7.82 (dd, *J* = 9.0, 2.1 Hz, 1H), 7.53–7.43 (m, 2H), 7.29–7.26 (m, 2H), 6.38 (d, *J* = 5.2 Hz, 1H), 4.22 (q, *J* = 7.1 Hz, 2H), 3.59–3.53 (m, 1H), 1.73–1.71 (m, 2H), 1.44–1.41 (m, 2H), 1.30–1.22 (m, 5H), 1.13–1.08 (m, 2H).
**Ethyl 1-cyclopropyl-7-((4-(1-(3,4-dimethylphenyl)cyclopropyl)pyrimidin-2-yl)ami no)-4-oxo-1,4-dihydroquinoline-3-carboxylate (9e):** Yield 22%; Yellow solid; ^1^H NMR (400 MHz, Chloroform-*d*) *δ* 8.58 (s, 1H), 8.42 (d, *J* = 8.8 Hz, 1H), 8.19 (d, *J* = 5.0 Hz, 1H), 7.61 (s, 1H), 7.42 (dd, *J* = 9.0, 2.2 Hz, 1H), 7.18−7.12 (m, 3H) 6.39 (d, *J* = 5.0 Hz, 1H), 7.11 (s, 2H), 6.98 (d, *J* = 4.9 Hz, 1H), 4.41 (q, *J* = 7.0 Hz, 2H), 3.54−3.49 (m, 1H), 2.32 (s, 3H), 2.30 (s, 3H), 2.34−2.30 (m, 1H), 2.31 (s, 3H), 2.30 (s, 3H), 1.79−1.76 (m, 2H), 1.43 (t, *J* = 7.0 Hz, 3H), 1.40−1.34 (m, 4H), 1.18−1.13 (m, 2H).
**Ethyl 7-((4-(3-cyano-1-(2,4-difluorophenyl)propyl)pyrimidin-2-yl)amino)-1-cyclop ropyl-4-oxo-1,4-dihydroquinoline-3-carboxylate (9f):** Yield 25%; White solid; ^1^H NMR (400 MHz, Chloroform-*d*) *δ* 8.67 (d, *J* = 2.0 Hz, 1H), 8.57 (s, 1H), 8.43 (d, *J* = 5.0 Hz, 1H), 8.41 (s, 1H), 7.91 (s, 1H), 7.50 (dd, *J* = 9.0, 2.0 Hz, 1H), 7.36−7.30 (m, 1H), 6.92−6.83 (m, 2H), 6.71 (d, *J* = 5.0 Hz, 1H), 4.43−4.36 (m, 3H), 3.53−3.48 (m, 1H), 2.70−2.63 (m, 1H), 2.41−2.31 (m, 3H), 1.40 (t, *J* = 7.0 Hz, 3H), 1.37−1.32 (m, 2H), 1.18−1.12 (m, 2H).
**Ethyl 7-((4-(3-cyano-1-(3,4-dimethylphenyl)propyl)pyrimidin-2-yl)amino)-1-cyclo propyl-4-oxo-1,4-dihydroquinoline-3-carboxylate (9g):** Yield 26%; White solid; ^1^H NMR (400 MHz, Chloroform-*d*) *δ* 8.56 (s, 1H), 8.42 (d, *J* = 8.8 Hz, 1H), 8.19 (d, *J* = 5.0 Hz, 1H), 7.59 (s, 1H), 7.41 (d, *J* = 9.0Hz, 1H), 7.18−7.12 (m, 3H), 6.39 (d, *J* = 5.0 Hz, 1H), 6.96 (d, *J* = 4.9 Hz, 1H), 4.41 (q, *J* = 7.4 Hz, 2H), 4.15 (t, *J* = 6.8 Hz, 1H), 3.78−3.72 (m, 1H), 2.60−2.56 (m, 1H), 2.45−2.40 (m, 2H), 2.34−2.30 (m, 1H), 2.25 (s, 3H), 2.24 (s, 3H), 1.39−1.31 (m, 5H), 1.21−1.17 (m, 2H).
**Ethyl 1-cyclopropyl-7-((4-(1-(2,4-difluorophenyl)-3-hydroxypropyl)pyrimidin-2-yl) amino)-4-oxo-1,4-dihydroquinoline-3-carboxylate (9h):** Yield 19%; White solid; ^1^H NMR (400 MHz, Chloroform-*d*) *δ* 8.71 (s, 1H), 8.56 (s, 1H), 8.40−8.38 (m, 2H), 7.66 (s, 1H), 7.41−7.35 (m, 2H), 6.92−6.81 (m, 2H), 6.72 (d, *J* = 4.9 Hz, 1H), 4.56 (t, *J* = 7.5 Hz, 2H), 4.40 (q, *J* = 7.2 Hz, 2H), 3.68 (t, *J* = 6.2 Hz, 2H), 3.55−3.50 (m, 1H), 2.61−2.54 (m, 1H), 2.32−2.25 (m, 1H), 1.43 (t, *J* = 6.2 Hz, 3H), 1.37−1.33 (m, 2H), 1.16−1.12 (m, 2H).
**Ethyl 1-cyclopropyl-7-((4-(3-(difluoromethoxy)-1-(2,4-difluorophenyl)propyl)pyrimidin-2-yl)amino)-4-oxo-1,4-dihydroquinoline-3-carboxylate (9j):** Yield 21%; White solid; ^1^H NMR (400 MHz, Chloroform-*d*) *δ* 8.91 (s, 1H), 8.65 (s, 1H), 8.56 (d, *J* = 5.6 Hz, 2H), 8.48 (d, *J* = 8.9 Hz, 1H), 8.16 (d, *J* = 9.0 Hz, 1H), 7.57−7.51 (m, 1H), 7.05 (d, *J* = 5.6 Hz, 1H), 7.01−6.98 (m, 1H), 6.90–6.84 (m, 1H), 6.22 (t, *J* = 76.4 Hz, 1H), 4.68 (t, *J* = 7.5 Hz, 1H), 4.52 (q, *J* = 7.1 Hz, 2H), 3.98−3.92 (m, 2H), 3.87−3.81 (m, 1H), 2.82−2.73 (m, 1H), 2.53−2.43 (m, 2H), 1.62−1.55 (m, 2H) 1.48 (t, *J* = 7.1 Hz, 3H), 1.27−1.23 (m, 2H).
**Ethyl 7-((4-(3-(*tert*-butoxy)-1-(2,4-difluorophenyl)-3-oxopropyl)pyrimidin-2-yl)amino)-1-cyclopr opyl-4-oxo-1,4-dihydroquinoline-3-carboxylate (9k):** Yield 26%; Yellow oil; ^1^H NMR (400 MHz, Chloroform-*d*) *δ* 8.69 (s, 1H), 8.57 (s, 1H), 8.42 (d, *J* = 8.8 Hz, 1H), 8.37 (d, *J* = 5.0 Hz, 1H), 7.91 (s, 1H), 7.45 (d, *J* = 8.9 Hz, 1H), 7.31−7.26 (m, 1H), 6.86−6.80 (m, 2H), 6.70 (d, J = 5.0 Hz, 1H), 4.77 (t, *J* = 8.0 Hz, 1H), 4.38 (q, *J* = 7.7 Hz, 2H), 3.56−3.51 (m, 1H), 3.34−3.28 (m, 1H), 2.91−2.85 (m, 1H), 1.40 (t, *J* = 7.7 Hz, 1H), 1.34−1.29 (m, 11H), 1.17−1.12 (m, 2H).
**Ethyl 1-cyclopropyl-7-((4-(1-(2,4-difluorophenyl)-3-morpholinopropyl)pyramidin-2-yl)amino)-4-oxo-1,4-dihydroquinoline-3-carboxylate (9l):** Yield 21%; White solid; ^1^H NMR (400 MHz, Chloroform-*d*) *δ* 8.63 (s, 1H), 8.57 (s, 1H), 8.42−8.39 (m, 2H), 7.75 (s, 1H), 7.48 (dd, *J* = 8.9, 2.1 Hz, 1H), 7.42−7.36 (m, 1H), 6.89−6.78 (m, 2H), 6.75 (d, *J* = 5.0 Hz, 1H), 4.42−4.37 (m, 3H), 3.68 (t, *J* = 4.7 Hz, 4H), 2.53−2.46 (m, 1H), 2.42−2.39 (m, 2H), 2.31 (t, *J* = 4.6 Hz, 4H), 2.21−2.12 (m, 1H) 1.41 (t, *J* = 7.1 Hz, 3H), 1.35−1.30 (m, 2H), 1.17−1.13 (m, 2H).
**Ethyl 1-cyclopropyl-7-((4-(1-(2,4-difluorophenyl)-3-(4,4-difluoropiperidin-1-yl)pro pyl)pyrimidin-2-yl)amino)-4-oxo-1,4-dihydroquinoline-3-carboxylate (9m):** Yield 23%; White solid; ^1^H NMR (400 MHz, Chloroform-*d*) *δ* 8.63 (d, *J* = 2.2 Hz, 1H), 8.60 (s, 1H), 8.45 (d, *J* = 9.0 Hz, 1H), 8.41 (d, *J* =5.0 Hz, 1H), 7.57−7.46 (m, 2H), 7.42−7.35 (m, 1H), 6.91−6.80 (m, 2H), 6.76 (d, *J* = 5.0 Hz, 1H), 4.45−4.39 (m, 3H), 3.51−3.45 (m, 1H), 2.58−2.45 (m, 5H), 2.41−2.35 (m, 2H), 2.22−2.13 (m, 1H), 2.04−1.91 (m, 4H), 1.44 (t, *J* = 7.1 Hz, 3H), 1.37−1.33 (m, 2H), 1.19−1.15 (m, 2H).
**Ethyl 1-cyclopropyl-7-((4-(1-(2,4-difluorophenyl)-3-(4-methylpiperazin-1-yl)propy l)pyrimidin-2-yl)amino)-4-oxo-1,4-dihydroquinoline-3-carboxylate (9n):** Yield 19%; White solid; ^1^H NMR (400 MHz, Chloroform-*d*) *δ* 8.64 (s, 1H), 8.59 (s, 1H), 8.44 (d, *J* = 8.9 Hz 1H), 8.39 (d, *J* = 5.0 Hz, 1H), 7.51 (d, *J* = 9.0 Hz, 1H), 7.40−7.34 (m, 1H), 6.90−6.74 (m, 2H), 6.75 (d, *J* = 5.0 Hz, 1H), 4.44−4.35 (m, 3H), 3.49−3.47 (m, 1H), 2.61−2.45 (m, 9H), 2.45−2.37 (m, 2H), 2.34 (s, 3H), 2.22−2.13 (m, 1H), 1.43 (t, *J* = 7.1 Hz, 3H), 1.37−1.32 (m, 2H), 1.18−1.14 (m, 2H).
**Ethyl 1-cyclopropyl-7-((4-(1-(2,4-difluorophenyl)-3-ethoxy-3-oxopropyl)pyramidin-2-yl) amino)-4-oxo-1,4-dihydroquinoline-3-carboxylate (9o):** Yield 24%; White solid; ^1^H NMR (400 MHz, Chloroform-*d*) *δ* 8.68–8.66 (m, 1H), 8.59 (s, 1H), 8.43 (d, *J* = 8.8 Hz, 1H), 8.38 (d, *J* = 5.0 Hz, 1H), 7.70 (s, 1H), 7.45 (dd, *J* = 9.0, 2.2 Hz, 2H), 7.32−7.26 (m, 1H), 6.89−6.82 (m, 2H), 6.73 (d, *J* = 5.0 Hz, 1H), 4.36 (q, *J* = 6.8 Hz, 2H), 4.09 (q, *J* = 7.1 Hz, 2H), 3.55−3.49 (m, 1H), 3.47−3.41 (m, 1H), 2.98−2.92 (m, 1H), 1.42 (t, *J* = 7.1 Hz, 3H), 1.38−1.33 (m, 2H), 1.18−1.15 (m, 5H).

#### 3.1.11. Procedure for the Synthesis of the Target Compounds **A1−14**

To a solution of intermediate **9** (0.5 mmol) in THF (5.0 mL) was added 5.0 M aqueous LiOH (3.0 mL), and the reaction mixture was stirred at 50 °C for 6.0 h. After completion, the mixture was cooled to room temperature, and THF was removed under reduced pressure. The residue was adjusted to pH ~2 by dropwise addition of 2.0 M HCl under ice-bath cooling with stirring. The resulting precipitate was collected by filtration, washed with water, and dried to afford the target compounds **A1–14**.

**1-Cyclopropyl-7-((4-(1-(2,4-difluorophenyl)ethyl)pyrimidin-2-yl)amino)-4-oxo-1,4-dihydroquinoline-3-carboxylic acid (A1):** Yield 97%; White solid; M.p. 256.2−256.7 °C; ^1^H NMR (400 MHz, DMSO-*d*_6_) *δ* 10.47 (s, 1H), 8.94 (s, 1H), 8.69 (s, 1H), 8.57 (d, *J* =5.0 Hz, 1H), 8.24 (d, *J* = 9.0 Hz, 1H), 7.99 (d, *J* = 8.8 Hz, 1H), 7.49−7.44 (m, 1H), 7.41−7.37 (m, 1H), 7.23−7.18 (m, 1H), 6.98 (d, *J* = 5.0 Hz, 1H), 4.28 (q, *J* = 7.1 Hz, 1H), 3.75−3.69 (m, 1H), 1.65 (d, *J* = 7.0 Hz, 3H), 1.33−1.27 (m, 2H), 1.22−1.17 (m, 2H); ^13^C NMR (101 MHz, DMSO-*d*_6_) *δ* 177.47, 173.65, 166.69, 159.75, 159.12, 149.81 (dd, *J* = 257.3, 11.1 Hz), 148.84, 148.72 (dd, *J* = 245.4, 13.2 Hz), 146.44, 142.96, 141.81 (dd, *J* = 5.1, 4.0 Hz), 126.78, 124.96 (dd, *J* = 6.1, 3.0 Hz), 119.05, 118.38, 117.91 (d, *J* = 17.2 Hz), 117.15 (d, *J* = 17.3 Hz), 112.61, 107.29, 105.01, 45.65, 36.13, 20.26, 8.10; HRMS *m*/*z* calcd for C_25_H_24_N_4_O_3_F_2_ [M + H]^+^: 463.1582, found 463.1573.
**1-Cyclopropyl-7-((4-(1-(2,4-difluorophenyl)heptyl)pyrimidin-2-yl)amino)-4-oxo-1,4-dihydroquinoline-3-carboxylic acid (A2):** Yield 96%; White solid; M.p. 215.2−216.0 °C; ^1^H NMR (400 MHz, DMSO-*d*_6_) *δ* 10.46 (s, 1H), 8.91 (s, 1H), 8.69 (s, 1H), 8.56 (d, *J* = 5.0 Hz, 1H), 8.23 (d, *J* = 9.0 Hz, 1H), 8.00 (d, *J* = 8.9 Hz, 1H), 7.60−7.54 (m, 1H), 7.23 (ddd, *J* = 10.7, 9.2, 2.6 Hz 1H), 7.14−7.09 (m, 1H), 6.97 (d, *J* = 5.0 Hz, 1H), 4.28 (t, *J* = 7.7 Hz, 1H), 3.76−3.69 (m, 1H), 2.26−2.18 (m, 1H), 2.06−1.97 (m, 1H), 1.35−1.28 (m, 4H), 1.26−1.17 (m, 8H), 0.83 (t, *J* = 6.8 Hz, 1H); ^13^C NMR (101 MHz, DMSO-*d*_6_) *δ* 177.47, 172.39, 166.70, 161.56 (dd, *J* = 246.2, 12.1 Hz), 160.70 (dd, *J* = 247.4, 12.1 Hz), 159.76, 159.04, 148.87, 146.43, 142.93, 130.94 (dd, *J* = 10.2, 6.1 Hz), 125.68, 119.06, 118.32, 112.91, 112.21 (dd, *J* = 20.2, 3.0 Hz), 107.28, 105.03, 104.30 (t, *J* = 25.6 Hz), 44.86, 36.11, 33.27, 31.52, 28.88, 27.52, 22.47, 14.34, 8.10; HRMS *m*/*z* calcd for C_30_H_30_N_4_O_3_F_2_ [M + H]^+^: 533.2365, found 533.2363.
**1-Cyclopropyl-7-((4-(1-(2,4-difluorophenyl)cyclopropyl)pyrimidin-2-yl)amino)-4-oxo-1,4-dihydroquinoline-3-carboxylic acid (A3):** Yield 97%; White solid; M.p. 156.8−157.6 °C; ^1^H NMR (400 MHz, DMSO-*d*_6_) *δ* 10.35 (s, 1H), 8.73 (s, 1H), 8.68 (s, 1H), 8.40 (d, *J* = 5.0 Hz, 1H), 8.19 (d, *J* = 8.6 Hz, 1H), 7.98 (dd, *J* = 8.2, 2.8 Hz, 1H), 7.59−7.53 (m, 1H), 7.33 (dt, *J* = 9.6, 2.7 Hz, 1H), 7.20−7.15 (m, 1H), 6.41 (d, *J* = 5.0 Hz, 1H), 3.77−3.72 (m, 1H), 1.80−1.78 (m, 2H), 1.45−1.42 (m, 2H), 1.36−1.31 (m, 2H), 1.23−1.19 (m, 2H); ^13^C NMR (101 MHz, DMSO-*d*_6_) *δ* 177.47, 172.98, 166.70, 162.62 (dd, *J* = 248.4, 13.6 Hz), 162.37 (dd, *J* = 245.3, 11.6 Hz), 159.69, 158.32, 148.84, 146.52, 142.88, 134.14 (dd, *J* = 9.2, 5.1 Hz), 126.74, 125.10 (dd, J = 15.2, 4.1 Hz), 119.03, 118.21, 112.14 (d, *J* = 22.2 Hz), 110.55, 107.24, 105.08, 104.68 (t, *J* = 26.3 Hz), 36.15, 25.96, 18.93, 8.15; HRMS *m*/*z* calcd for C_26_H_20_N_4_O_3_F_2_ [M + H]^+^: 457.1582, found 457.1583.
**1-Cyclopropyl-7-((4-(1-(3,4-difluorophenyl)cyclopropyl)pyrimidin-2-yl)amino)-4-oxo-1,4-dihydroquinoline-3-carboxylic acid (A4):** Yield 95%; White solid; M.p. 153.5−154.2 °C; ^1^H NMR (400 MHz, DMSO-*d*_6_) *δ* 10.35 (s, 1H), 8.70 (s, 1H), 8.68 (s, H), 8.40 (d, *J* = 4.8 Hz, 1H), 8.20 (d, *J* = 9.0 Hz, 1H), 7.98 (d, *J* = 8.8 Hz, 1H), 7.54−7.44 (m, 2H), 6.42 (d, *J* = 5.0 Hz, 1H), 3.79−3.69 (m, 1H), 1.77−1.69 (m, 2H), 1.48−1.41 (m, 2H), 1.36−1.30 (m, 2H), 1.22−1.16 (m, 2H); ^13^C NMR (101 MHz, DMSO-*d*_6_) *δ* 177.45, 173.54, 166.68, 159.67, 158.21, 149.76 (dd, *J* = 247.3, 12.2 Hz), 149.20 (dd, *J* = 246.5, 14.1 Hz), 148.81, 146.91, 142.89, 139.54, 128.02 (dd, *J* = 6.1, 3.0 Hz), 126.75, 120.19 (d, *J* = 16.2 Hz), 119.02, 118.20 (d, *J* = 11.1 Hz), 117.98, 111.76, 107.26, 104.98, 36.14, 31.01, 19.14, 8.15; HRMS *m*/*z* calcd for C_26_H_20_N_4_O_3_F_2_ [M + H]^+^: 475.1582, found 475.1583.
**1-Cyclopropyl-7-((4-(1-(3,4-dimethylphenyl)cyclopropyl)pyrimidin-2-yl)amino)-4-oxo-1,4-dihydroquinoline-3-carboxylic acid (A5):** Yield 87%; Yellow solid; M.p. 150.6−151.3 °C; ^1^H NMR (400 MHz, DMSO-*d*_6_) *δ* 10.09 (s, 1H), 8.72 (s, 1H), 8.63 (s, H), 8.32 (d, *J* = 5.1 Hz, 1H), 8.17 (d, *J* = 9.0 Hz, 1H), 7.81 (d, *J* = 8.8 Hz, 1H), 7.18−7.16 (m, 2H), 7.12 (d, *J* = 7.6 Hz, 1H), 6.32 (d, *J* = 5.2 Hz, 1H), 3.62−3.56 (m, 1H), 2.24 (s, 6H), 1.72–1.69 (m, 2H), 1.35−1.27 (m, 4H), 1.10−1.04 (m, 2H); ^13^C NMR (101 MHz, DMSO-*d*_6_) *δ* 176.81, 174.46, 166.83, 159.94, 157.87, 149.01, 144.82, 142.25, 139.18, 136.90, 135.65, 132.15, 130.21, 128.39, 127.12, 121.73, 117.13, 116.70, 111.73, 104.23, 34.72, 31.27, 19.90, 19.54, 18.69, 8.15; HRMS *m*/*z* calcd for C_28_H_26_N_4_O_3_ [M + H]^+^: 467.2083, found 467.2087.
**7-((4-(3-Cyano-1-(2,4-difluorophenyl)propyl)pyrimidin-2-yl)amino)-1-cyclopropyl-4-oxo-1,4-dihydroquinoline-3-carboxylic acid (A6):** Yield 77%; White solid; M.p. 231.6−232.3 °C; ^1^H NMR (400 MHz, DMSO-*d*_6_) *δ* 10.53 (s, 1H), 8.93 (d, *J* = 2.1 Hz, 1H), 8.70 (s, 1H), 8.59 (d, *J* = 5.0 Hz, 1H), 8.25 (d, *J* = 9.0 Hz, 1H), 8.00 (dd, *J* = 8.9, 2.0 Hz, 1H), 7.60–7.54 (m, 1H), 7.28 (ddd, *J* = 10.8, 9.2, 2.7 Hz, 1H), 7.17–7.12 (m, 1H), 6.96 (d, *J* = 5.0 Hz, 1H), 4.41 (t, *J* = 7.6 Hz, 1H), 3.78−3.72 (m, 1H), 2.64−2.54 (m, 3H), 2.39−2.30 (m, 1H), 1.36−1.31 (m, 2H), 1.23−1.18 (m, 2H); ^13^C NMR (101 MHz, DMSO-*d*_6_) *δ* 177.47, 170.67, 166.70, 161.94 (dd, *J* = 247.2, 13.1 Hz), 160.66 (dd, *J* = 247.3, 13.2 Hz), 159.77, 159.35, 148.88, 146.30, 142.93, 131.09 (dd, *J* = 10.1, 6.0 Hz), 126.84, 124.21 (dd, *J* = 13.1, 3.0 Hz), 120.41, 119.15, 118.37, 112.96, 112.48 (dd, *J* = 21.1, 3.0 Hz), 105.10, 104.62 (t, J = 26.3 Hz), 43.92, 36.11, 28.53, 15.30, 8.12; HRMS *m*/*z* calcd for C_27_H_21_N_5_O_3_F_2_ [M + H]^+^: 502.1691, found 502.1689.
**1-Cyclopropyl-7-((4-(1-(3,4-difluorophenyl)cyclopropyl)pyrimidin-2-yl)amino)-6-(dimethylamino)-4-oxo-1,4-dihydroquinoline-3-carboxylic acid (A7)**: Yield 74%; Yellow solid; M.p. 227.5−228.1 °C; ^1^H NMR (400 MHz, DMSO-*d*_6_) *δ* 10.49 (s, 1H), 8.96 (d, *J* = 2.0 Hz, 1H), 8.70 (s, 1H), 8.54 (d, *J* = 5.0 Hz, 1H), 8.26 (d, *J* = 8.8 Hz, 1H), 8.01 (dd, J = 8.9, 2.2 Hz, 1H), 7.16 (s, 1H), 7.11 (s, 2H), 6.98 (d, *J* = 4.9 Hz, 1H), 4.05 (t, *J* = 7.8 Hz, 1H), 3.78−3.72 (m, 1H), 2.59−2.56 (m, 1H), 2.47−2.42 (m, 2H), 2.38−2.33 (m, 1H), 2.20 (s, 3H), 2.18 (s, 3H), 1.34−1.32 (m, 2H), 1.22−1.19 (m, 2H); ^13^C NMR (101 MHz, DMSO-*d*_6_) *δ* 177.47, 172.23, 166.69, 159.72 158.92, 148.86, 146.44, 142.99, 138.49, 137.06, 135.59, 130.34, 129.49, 126.82, 125.68, 120.62, 119.06, 118.44, 113.19, 107.31, 104.98, 51.25, 36.15, 29.27, 19.96, 19.42, 15.38, 8.16; HRMS *m*/*z* calcd for C_29_H_27_N_5_O_3_ [M + H]^+^: 494.2192, found 494.2197.
**1-Cyclopropyl-7-((4-(1-(2,4-difluorophenyl)-3-hydroxypropyl) pyrimidin-2-yl)ami no)-4-oxo-1,4-dihydroquinoline-3-carboxylic acid (A8):** Yield 57%; Yellow solid; M.p. 229.1−229.6 °C; ^1^H NMR (400 MHz, DMSO-*d*_6_) *δ* 10.48 (s, 1H), 8.97 (s, 1H), 8.70 (s, 1H), 8.56 (d, *J* = 5.0 Hz, 1H), 8.23 (d, *J* = 9.0 Hz, 1H), 7.96 (dd, 9.0, 1.8 Hz, 1H), 7.60−7.54 (m, 1H), 7.26−7.20 (m, 1H),7.15−7.10 (m, 1H), 6.94 (d, *J* = 5.0 Hz, 1H), 4.61 (t, *J* = 5.0 Hz, 1H), 4.51 (t, *J* = 7.7 Hz, 1H), 3.77−3.72 (m, 1H), 3.43−3.35 (m, 2H), 2.44−2.37 (m, 1H), 2.21−2.13 (m, 1H), 1.37−1.31 (m, 2H), 1.22−1.17 (m, 2H); ^13^C NMR (101 MHz, DMSO-*d*_6_) *δ* 177.47, 172.33, 166.70, 161.12 (dd, *J* = 246.4, 13.6 Hz), 160.68 (dd, *J* = 246.2, 13.2 Hz), 159.77, 159.07, 148.83, 146.43, 142.98, 131.16 (dd, *J* = 9.1, 5.1 Hz), 126.79, 125.48 (dd, J = 14.1, 4.0 Hz), 119.07, 118.39, 112.94, 112.16 (d, *J* = 19.3 Hz), 107.29, 105.01, 104.38 (t, *J* = 26.3 Hz), 58.74, 41.39, 36.19, 36.18, 8.89; HRMS *m*/*z* calcd for C_26_H_22_N_4_O_4_F_2_ [M + H]^+^: 493.1687, found 493.1687.
**1-Cyclopropyl-7-((4-(3-(difluoromethoxy)-1-(2,4-difluorophenyl)propyl)pyrimidin-2-yl)amino)-4-oxo-1,4-dihydroquinoline-3-carboxylic acid (A9):** Yield 93%; Yellow solid; M.p. 219.3−219.8 °C; ^1^H NMR (400 MHz, DMSO-*d*_6_) *δ* 10.52 (s, 1H), 8.93(s, 1H), 8.69 (s, 1H), 8.57 (d, *J* = 5.2 Hz, 1H), 8.05–8.00 (d, *J* = 9.0 Hz, 1H), 7.99 (dd, *J* = 9.0, 1.9 Hz, 1H), 7.61−7.55 (m, 1H), 7.28−7.22 (m, 1H), 7.16−7.11 (m, 1H), 6.95 (d, *J* = 5.2 Hz, 1H), 6.64 (t, *J* = 80.3 Hz, 1H), 4.48 (t, *J* = 8.0 Hz, 1H), 2.87−3.71 (m, 3H), 2.65−2.57 (m, 1H), 2.40−2.32 (m, 1H), 1.36−1.29 (m, 2H), 1.22−1.17 (m, 2H); ^13^C NMR (101 MHz, DMSO-*d*_6_) *δ* 177.47, 171.32, 166.74, 161.80 (dd, *J* = 245.6, 14.3 Hz), 160.76 (dd, *J* = 246.2, 12.6 Hz), 159.75, 159.28, 148.87, 146.38, 142.93, 131.16 (dd, *J* = 10.3, 7.2 Hz), 126.79, 124.65 (dd, *J* = 15.6, 4.4 Hz), 131.83, 128.93, 119.09, 118.40, 117.40 (t, *J* = 255.3 Hz), 112.37 (dd, *J* = 22.3, 4.5 Hz), 107.27, 105.09, 104.52 (t, *J* = 25.1 Hz) 62.81, 41.20, 36.14, 32.38, 8.09; HRMS *m*/*z* calcd for C_27_H_22_N_4_O_4_F_4_ [M + H]^+^: 543.1655, found 543.1656.
**1-Cyclopropyl-7-((4-(3,4-difluorophenoxy)pyrimidin-2-yl)amino)-6-fluoro-4-oxo-1,4-dihydroquinoline-3-carboxylic acid (A10):** Yield 99%; White solid; M.p. 186.2−186.7 °C; ^1^H NMR (400 MHz, DMSO-*d*_6_) *δ* 10.50 (s, 1H), 8.91 (d, *J* = 2.0 Hz, 1H), 8.70 (s, 1H), 8.56 (d, *J* = 5.0 Hz, 1H), 8.24 (d, *J* = 9.0 Hz, 1H), 7.99 (dd, *J* = 8.4, 2.0 Hz, 1H), 7.54−7.48 (m, 1H), 7.25 (ddd, *J* = 10.8, 9.2, 2.1 Hz, 1H), 7.13−7.08 (m, 1H), 4.74 (t, *J* = 7.7 Hz, 1H), 3.79−3.73 (m, 1H), 3.35−3.33 (m, 1H), 3.04−2.98 (m, 1H), 1.36−1.30 (m, 2H), 1.24−1.19 (m, 2H); ^13^C NMR (101 MHz, DMSO-*d*_6_) *δ* 177.48, 172.79, 171.10, 166.70, 161.81 (dd, *J* = 245.4, 12.6 Hz), 160.45 (dd, *J* = 254.5, 14.8 Hz), 159.65, 159.16, 148.85, 146.33, 142.93, 131.08 (dd, *J* = 9.1, 5.4 Hz), 126.80 (d, *J* = 7.2 Hz), 125.95 (dd, *J* = 14.3, 3.6 Hz), 107.29, 105.12, 104.52 (t, *J* = 26.2 Hz), 112.23 (d, *J* = 20.5 Hz), 41.30, 37.51, 36.14, 8.11; HRMS *m*/*z* calcd for C_26_H_20_F_2_N_4_O_5_ [M + H]^+^: 507.1481, found 507.1480.
**7-((4-(3-(*tert*-Butoxy)-1-(2,4-difluorophenyl)-3-oxopropyl)pyrimidin-2-yl)amino)-1-cyclopropyl-4-oxo-1,4-dihydroquinoline-3-carboxylic acid (A11):** Yield 93%; Yellow solid; M.p. 169.4−170.3 °C; ^1^H NMR (400 MHz, DMSO-*d*_6_) *δ* 10.51 (s, 1H), 8.92 (s, 1H), 8.71 (s, 1H), 8.56 (d, *J* = 5.0 Hz, 1H), 8.25 (d, *J* = 8.9 Hz, 1H), 7.99 (dd, *J* = 8.9, 2 Hz, 1H), 7.56−7.50 (m, 1H), 7.30−7.24 (m, 1H), 7.15−7.09 (m, 1H), 6.95 (d, *J* = 5.0 Hz, 1H), 4.72 (t, 7.6 Hz, 1H), 3.80–3.74 (m, 1H), 3.30–3.27 (m, 1H), 3.04−2.98 (m, 1H), 1.36−1.13 (m, 13H); ^13^C NMR (101 MHz, DMSO-*d*_6_) *δ* 177.49, 172.79, 170.81, 170.38, 166.68, 164.16 (dd, *J* = 213.3, 13.2 Hz), 160.17 (dd, *J* = 253.6, 16.1 Hz), 159.66, 159.22, 148.92, 146.32, 142.96, 131.17 (dd, *J* = 16.4, 7.3 Hz), 126.83, 119.15, 118.46, 112.85, 112.17 (d, *J* = 8.5 Hz), 107.32, 105.11, 104.50 (t, *J* = 26.2 Hz), 80.61, 41.53, 38.73, 27.92, 8.10; HRMS *m*/*z* calcd for C_30_H_28_N_4_O_5_F_2_ [M + H]^+^: 563.2106, found 563.2104.
**1-Cyclopropyl-7-((4-(1-(2,4-difluorophenyl)-3-morpholinopropyl)pyrimidin-2-yl)amino)-4-oxo-1,4-dihydroquinoline-3-carboxylic acid (A12):** Yield 67%; White solid; M.p. 240.9–241.4 °C; 1H NMR (400 MHz, DMSO-*d6*) *δ* 10.47 (s, 1H), 8.92 (s, 1H), 8.70 (s, 1H), 8.56 (d, *J* = 5.0 Hz, 1H), 8.23 (d, *J* = 9.2 Hz, 1H), 7.99 (d, *J* = 8.0 Hz, 1H), 7.60−7.54 (m, 1H), 7.22 (ddd, *J* = 10.7, 9.2, 2.8 Hz, 1H), 7.14−7.09 (m, 1H), 6.99 (d, *J* = 4.9 Hz, 1H), 4.42 (t, *J* = 7.7 Hz, 1H), 3.76−3.72 (m, 1H), 3.52 (t, *J* = 4.6 Hz), 2.47−2.40 (m, 1H), 2.34−2.29 (m, 6H), 2.20−2.13 (m, 1H), 1.35−1.30 (m, 2H), 1.23−1.19 (m, 2H); 13C NMR (101 MHz, DMSO-*d6*) *δ* 177.47, 172.23, 166.69, 166.01 (dd, *J* = 198.2, 19.4 Hz), 160.64 (dd, *J* = 247.3, 13.6 Hz), 159.75, 158.99, 148.88, 146.42, 142.95, 131.07 (dd, *J* = 10.4, 6.2 Hz), 126.79, 125.75 (dd, 15.3, 4.6 Hz), 119.08, 118.34, 113.04, 112.17 (dd, *J* = 20.5, 3.2 Hz), 105.01, 104.59, 104.33, 66.66, 56.55, 53.79, 42.91, 36.12, 30.19, 8.10; HRMS *m*/*z* calcd for C30H29N5O4F2 [M + H]^+^: 562.2266, found 562.2267.
**1-Cyclopropyl-7-((4-(1-(2,4-difluorophenyl)-3-(4,4-difluoropiperidin-1-yl)propyl) pyrimidin-2-yl)amino)-4-oxo-1,4-dihydroquinoline-3-carboxylic acid (A13):** Yield 57%; White solid; M.p. 243.7–244.3 °C; ^1^H NMR (400 MHz, DMSO-*d*_6_) *δ* 11.39 (s, 1H), 10.51 (s, 1H), 8.91 (s, 1H), 8.68 (s, 1H), 8.57 (d, *J* = 5.0 Hz, 1H), 8.22 (d, J = 9.0 Hz, 1H), 7.62−7.56 (m, 1H), 7.27−7.20 (m, 1H), 7.16−7.09 (m, 1H), 7.00 (d, *J* = 5.0 Hz, 1H), 4.39 (t, *J* = 7.7 Hz, 1H), 3.77−3.70 (m, 1H), 3.66−3.57 (m, 2H), 3.20−3.05 (m, 4H), 2.80−2.69 (m, 1H), 2.48−2.27 (m, 5H), 1.36–1.30 (m, 2H), 1.23–1.17 (m, 2H); ^13^C NMR (101 MHz, DMSO-*d*_6_) *δ* 177.47, 166.72, 165.53 (dd, *J* = 256.4, 11.3 Hz), 160.56 (dd, *J* = 243.3, 11.2 Hz), 159.74, 159.27, 148.85, 146.44, 142.91, 140.73, 134.07, 131.13 (dd, *J* = 8.6, 4.4 Hz), 126.80, 125.70, 119.05, 118.35, 113.05, 112.25 (d, *J* = 25.3 Hz), 107.26, 105.10, 104.37, 54.99, 49.91, 42.83, 36.14, 30.81, 29.29, 8.13; HRMS *m*/*z* calcd for C_31_H_29_N_5_O_3_F_4_ [M + H]^+^: 596.2285, found 596.2283.
**1-Cyclopropyl-7-((4-(1-(2,4-difluorophenyl)-3-(4-methylpiperazin-1-yl)propyl) pyrimidin-2-yl)amino)-4-oxo-1,4-dihydroquinoline-3-carboxylic acid (A14):** Yield 44%; Yellow solid; M.p. 271.6−271.9 °C; ^1^H NMR (400 MHz, DMSO-*d*_6_) *δ* 11.40 (br, 1H), 10.55 (s, 1H), 8.86 (s, 1H), 8.70 (s, 1H), 8.61 (d, *J* = 5.0 Hz, 1H), 8.26 (d, *J* = 8.9 Hz, 1H), 8.07 (dd, *J* = 9.0, 2.0 Hz, 1H), 7.64−7.58 (m, 1H), 7.31−7.25 (m, 1H), 7.19−7.14 (m, 1H), 7.01 (d, *J* = 5.0 Hz, 1H), 4.39 (t, *J* = 4.1 Hz, 1H), 3.76−3.71 (m, 1H), 3.68−3.57 (m, 2H), 3.21−3.07 (m, 4H), 2.78−2.73 (m, 1H), 2.50 (s, 3H), 2.46−2.40 (m, 1H), 2.36−2.27 (m, 2H), 1.34−1.32 (m, 2H), 1.22–1.18 (m, 2H); HRMS *m*/*z* calcd for C_31_H_32_N_6_O_3_F_2_ [M + H]^+^: 575.2582, found 575.2582.

#### 3.1.12. Procedure for the Synthesis of the Target Compounds **A15** and **A16**

To a solution of intermediate **6** (2.0 mmol) in a mixture solution of ethanol (3.0 mL) and water (3.0 mL) was added 5.0 N aqueous NaOH (4.0 mL), and the reaction mixture was stirred at 50 °C for 3.0 h. After completion, the mixture was cooled to room temperature, and the solvent was removed under reduced pressure. The residue was adjusted to pH ~7 by dropwise addition of 2.0 M HCl under ice-bath cooling with stirring. The resulting precipitate was collected by filtration, washed with water, and dried to afford the intermediate **10**, which was used directly for the next step without further purification.

To a Schlenk tube were added intermediate **10** (0.75 mmol), the appropriate intermediate **8q** or **8r** (0.50 mmol), anhydrous K_2_CO_3_ (104 mg, 0.75 mmol), BINAP (44 mg, 0.07 mmol), Pd(OAc)_2_ (12 mg, 0.05 mmol), and anhydrous toluene (8.0 mL). The mixture was refluxed under nitrogen for 10–12 h. After cooling to room temperature, the reaction mixture was filtered through Celite, and the filtrate was concentrated under reduced pressure. The residue was purified by silica gel column chromatography (CH_2_Cl_2_/MeOH = 30:1–10:1, *v*/*v*) to give intermediate **A15** or **A16**.

**1-Cyclopropyl-7-((4-(1-(2,4-difluorophenyl)-3-(4-methylpiperazin-1-yl)-3-oxopropyl) pyrimidin-2-yl)amino)-4-oxo-1,4-dihydroquinoline-3-carboxylic acid (A15):** Yield 15%; Yellow solid; M.p. 257.6–258.3 °C; ^1^H NMR (400 MHz, DMSO-*d*_6_) *δ* 10.47 (s, 1H), 8.90 (s, 1H), 8.70 (s, 1H), 8.55 (d, *J* = 5.1 Hz, 1H), 8.25 (d, *J* = 9.0 Hz, 1H), 8.01 (dd, *J* = 8.2, 2.0 Hz, 1H), 7.52−7.46 (m, 1H), 7.27−7.22 (m, 1H), 7.12−7.08 (m, 1H), 6.95 (d, *J* = 5.2 Hz, 1H), 4.82 (t, *J* = 7.4 Hz, 1H), 3.75−3.70 (m, 1H), 3.54−3.42 (m, 5H), 3.09−3.04 (m, 1H), 2.27−2.13 (m, 7H), 1.34–1.30 (m, 2H), 1.23–1.18 (m, 2H); HRMS *m*/*z* calcd for C_31_H_30_N_6_O_4_F_2_ ([M + H]^+^): 589.2375, found 589.2375.
**1-Cyclopropyl-7-((4-(1-(3,4-dimethylphenyl)-3-(4-methylpiperazin-1-yl)-3-oxo propyl)pyrimidin-2-yl)amino)-4-oxo-1,4-dihydroquinoline-3-carboxylic acid (A16):** Yield 16%; Yellow solid; M.p. 251.8–252.6 °C; ^1^H NMR (400 MHz, DMSO-*d*_6_) *δ* 10.43 (s, 1H), 8.93 (s, 1H), 8.69 (s, 1H), 8.50 (d, *J* = 5.0 Hz, 1H), 8.56 (d, *J* = 8.9 Hz, 1H), 8.03 (dd, *J* = 9.0, 0.9 Hz, 1H) 7.81 (d, *J* = 2.1 Hz,1H), 7.74 (dd, *J* =7.9, 1 Hz, 1H), 7.14 (s, 1H), 7.09−7.05 (m, 2H), 7.00 (d, *J* = 5.0 Hz, 1H), 4.48 (t, *J* = 6.2 Hz, 1H), 3.76−3.70 (m, 1H), 3.53−3.40 (m, 5H), 2.99−2.94 (m, 1H), 2.26−2.12 (m, 13H), 1.35−1.29 (m, 2H), 1.22−1.18 (m, 2H); ^13^C NMR (101 MHz, DMSO-*d*_6_) *δ* 177.48, 173.47, 169.13, 166.71, 159.54, 158.43, 148.86, 146.55, 142.98, 139.78, 136.66, 135.06, 130.03, 129.63, 126.83, 125.78, 118.99, 118.34, 113.26, 107.27, 104.99, 55.20, 54.81, 48.74, 46.08, 45.29, 41.53, 36.97, 36.15, 19.97, 19.38, 8.11; HRMS *m*/*z* calcd for C_33_H_36_N_6_O_4_ ([M + H]^+^): 581.2877, found 581.2880.

### 3.2. In Vitro Antibacterial Assay [24,25]

MRSA ATCC 33591 and *E. coli* ATCC 25922 were obtained from the American Type Culture Collection (ATCC, Manassas, VA, USA; https://www.atcc.org/). USA500 and Mu50 strains were kindly provided by Dr. Ying Zhang (Johns Hopkins University). These strains were obtained directly from his laboratory and are not associated with accession numbers in a public genetic database.

Prior to the experiment, a single colony was picked from TSA plate. All strains were grown at 37 °C overnight in TSB without the antibiotic. 1000-fold dilution of overnight cultures were grown at 37 °C for 2–3 h until A_600_ = 0.6. Then bacteria were diluted 1:400 into fresh TSB medium, compounds were dissolved in DMSO to a concentration of 10 mg/mL, and the solutions were diluted with medium before antibacterial activity test. Then, the stock solution was stocked at −20 °C. Ciprofloxacin, vancomycin and **Hyb-1** were used as the reference drugs. Equal volume of bacteria and compounds were added to 96 well plates and mixed well by shaking. Plates were incubated at 37 °C for 16–18 h followed by observations of MIC values by the absence or presence of visible growth. All experiments were done in duplicates and repeated for at least three times. MIC was determined as the lowest concentration needed to inhibit bacterial growth.

### 3.3. Propensity to Induce Bacterial Resistance [24]

Resistance development by sequential passaging. The MIC value of compound **A3**, **A5** and ciprofloxacin was determined against *S. aureus* USA500 in TSB medium by two-fold dilution method. For resistance development assay, the overnight cultural was diluted 1:1000 into fresh TSB medium with sub inhibitory concentration of antibiotics, and incubated at 37 °C, 200 rpm. The bacteria were passaged at 24-h intervals for 15 days, with increasing drug concentration according to the MIC values of a day before.

### 3.4. Cytotoxicity Assay [24]

NCM460 cell line was purchased from Shanghai Zeye Biotechnology Co., Ltd., Shanghai, China. A549 cell line was obtained from the Cell Bank of the Chinese Academy of Sciences (https://www.cellbank.org.cn/) and was maintained in our laboratory through routine passage and cryopreservation.

The cytotoxicity of compounds was evaluated using the cell counting kit-8 (CCK-8) assay (TargetMol, Shanghai, China). NCM460 or A549 cells (5 × 10^4^ cells/well) were cultured for 24 h, then treated with various concentrations of the compound for 24 h. Each concentration was tested in duplicate wells. Subsequently, the cells were incubated for 1 h with a CCK-8/DMEM mixture (1:10, 100 μL/well). The absorbance was measured at 450 nm using a microplate reader, and the average absorbance values of duplicate wells were used. Cell viability was expressed as a percentage of the control culture value, and the IC_50_ was calculated using GraphPad Prism software 8.0.2.

### 3.5. Syncropatch 384 Automated Patch-Clamp Herg Assay

CHO cells stably expressing the transcript of hERG were investigated by the automated whole-cell patch clamp technique, using the SyncroPatch 384 (Nanion, Munich, Germany). Cells were grown in 37 °C, 5% CO_2_ incubator, cultured in F12 medium with addition of 10% fetal bovine serum and 100 ug/mL G418 and 100 ug/mL Hygromycin B. Before assay, aliquots of dissociated cell suspension were plated into T-175 flasks and grown as above in a 5% CO_2_ incubator at 37 °C for 1–2 days. After reaching 60–80% confluence, cells were rinsed with 7 mL PBS (Phosphate-Buffered Saline) and incubated in 3 mL Detachin for 2 min at 37 °C. Following this, cells were rinsed and triturated gently in 7 mL culture medium, centrifuged and supernatant removed, then cells were resuspended in the external solution at a density of 5 × 10^5^ cells/mL. Cells were allowed to rest at 15 °C for 30 min in the cell hotel of the SyncroPatch 384 and shaken at 200 rmp to prevent aggregation prior to addition to the single hole NPC-384 chip for each experiment. NPC-384 chips were filled with internal and external solutions followed by the cell suspension. Nanion standard solutions were used for all recordings based on the following recipes: external solution (in mM) 140 NaCl, 4 KCl, 2 CaCl_2_, 1 MgCl_2_, 10 HEPES and 5 Glucose, pH 7.4 with NaOH; internal solution (in mM) 110 KF, 10 KCl, 10 NaCl, 10 EGTA and 10 HEPES, pH 7.2 with KOH. Suction was used to capture and seal the cells on the NPC-384 chip using optimized protocols. The cells were held at −80 mV and activated by a +40 mV pre-pulse of 2 s duration followed by a step to −50 mV for 1 s. The voltage protocol is to be repeated every 10 s, the peak of the tail current evoked by the −50 mV step was recorded and measured for calculating the percentage inhibition. The solutions containing the compounds were applied to the cells for 3 min for each concentration following a 3 min baseline recording in external solution. Each cell was received from six escalating concentrations. Each concentration was tested on at least 3 cells (*n* ≥ 3). The reference compound cisapride 300 nM was applied at the end of the test compound addition. The SyncroPatch 384 platform has a software package consisting of PatchControl 384 for data acquisition and DataControl 384 for data analysis (both from Nanion Technologies GmbH, Munich, Germany). Data were retrieved and the concentrations of compounds to yield 50% block of the hERG currents (IC_50_) analysis using Hill equation was done with DataControl 384.

## 4. Conclusions

In summary, based on our previous studies, we designed and synthesized a series of novel quinolone–aminopyrimidine hybrids by introducing substituted carbon chains at the benzyl position, a potential metabolic liability of this scaffold. This structural modification was intended to generate antibacterial agents with potent anti-MRSA activity, favorable in vitro metabolic stability, and low cytotoxicity. Among the synthesized hybrids, **A3** and **A5** displayed promising antibacterial activity against MRSA ATCC 33591, the fluoroquinolone-sensitive clinical isolate USA500, and the fluoroquinolone-resistant/vancomycin-intermediate clinical isolate Mu50. In addition, both compounds exhibited minimal cytotoxicity toward A549 and NCM460 cells and showed a low propensity for resistance development. Notably, **A5** demonstrated favorable metabolic stability in both HLMs and RLMs, whereas **A3** showed extremely prolonged half-lives, suggesting a potential risk of in vivo accumulation. Overall, these findings identify **A5** as the more promising lead for further optimization and antibacterial development.

## Figures and Tables

**Table 1:** Antibacterial activities of desquinolone-aminopyrimidine hybrids **A** against MRSA and *E. Coli* strains ^a^.

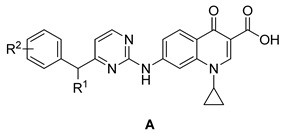				
Compd	R^1^	R^2^	MIC (μg/mL)	
			MRSA (ATCC33591)	*E. coli* (ATCC25922)
**A1**	Methyl	2,4-diFluoro	4	>64
**A2**	*n*-Hexyl	2,4-diFluoro	8	>64
**A3**	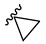	2,4-diFluoro	2	>64
**A4**	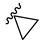	3,4-diFluoro	32	>64
**A5**	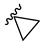	3,4-diMethyl	2	>64
**A6**	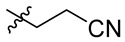	2,4-diFluoro	8	>64
**A7**	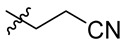	3,4-diMethyl	4	>64
**A8**	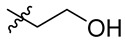	2,4-diFluoro	16	>64
**A9**	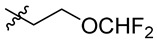	2,4-diFluoro	8	>64
**A10**	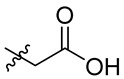	2,4-diFluoro	32	>64
**A11**	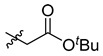	2,4-diFluoro	4	>64
**A12**	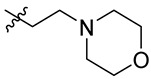	2,4-diFluoro	>64	>64
**A13**	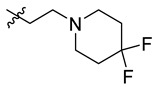	2,4-diFluoro	4	>64
**A14**	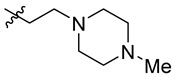	2,4-diFluoro	>32	>64
**A15**	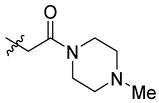	2,4-diFluoro	>64	>64
**A16**	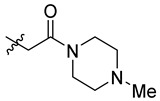	3,4-diMethyl	64	>64
**Hyb-1**			2	>64
Cip.^b^			0.5	0.015
Van.^b^			2	>64

^a^ All experiments were performed in at least triplicates. ^b^ Ciprofloxacin. Van.: Vancomycin.

**Table 2:** Antibacterial activities of desquinolone-aminopyrimidine hybrids **A** against MRSA strains USA500 and Mu50 ^a^.

Compd	MIC (μg/mL)	
	USA500	Mu50
**A1**	5	5
**A3**	1.25	1.25
**A5**	1.25	2.5
**A7**	5	5
**A11**	10	20
**A13**	5	5
**Hyb-1**	0.63	1.25
Cip.	1.25	>20
Van.	1.25	5

^a^ All experiments were performed in at least triplicates.

**Table 3:** Metabolic stability in liver microsomes.

Compd.	Species	k ^a^	T_1/2_^b^ (min)	Cl_int(mic)_^c^ (μL/min/mg)	Cl_int(liver)_^d^ (mL/min/kg)	% Remaining (T = 60 min)
**A3**	human	0.0000703	9863	0.141	0.133	97.1%
	rat	0.000724	983	1.45	2.61	86.1%
**A5**	human	0.0101	68.8	20.2	19.0	56.6%
	rat	0.0271	25.5	54.3	97.7	18.9%
**Hyb-1**	human	0.00795	87.9	15.9	15.0	61.6%
	rat	0.0129	53.6	25.9	46.6	44.7%
Midazolam		0.0948	7.31	474	448	15.3%

^a^ Drug elimination rate constant k (1/min). ^b^ Elimination half-life T_1/2_ (min). T_1/2_ = 0.693/k. ^c^ CL_int(mic)_ is the intrinsic clearance. CL_int(mic)_ = (0.693/half-life)/mg micro some protein per mL. ^d^ CL_int(liver)_ = CL_int(mic)_ × mg microsomal protein/g liver weight × g liver weight/kg body weight.

## Data Availability

Data will be made available on request.

## Supplementary Materials

The following supporting information can be downloaded at: https://www.mdpi.com/article/10.3390/molecules31101633/s1, NMR spectra of final compounds (PDF), HPLC spectra of compounds A3, and A5 (PDF).
