# Supplementary material for: Benzyl-Modified Quinolone-Aminopyrimidine Hybrids with Potent Anti-MRSA Activity and a High Barrier to Resistance
Source: Molecules. 2026 May 12;31(10):1633. doi: 10.3390/molecules31101633 (PMC13209803; doi:10.3390/molecules31101633)
Supplement: Supplementary file 1 [file molecules-31-01633-s001.zip › molecules-4285407-supplementary.pdf]

Supporting Information

Benzyl-Modified Quinolone-Aminopyrimidine Hybrids with Potent Anti-MRSA Activity and a High Barrier to Resistance

Xinghua Xu,[a] *Tao Xu*,[b] *Peng Cui*,[b] *Renhua Fan*,[a] *Qiuqin He*[a,*]

[a]Department of Chemistry, Fudan University, 2005 Songhu Road, Yangpu District, Shanghai, China

[b]Department of Infectious Diseases, National Medical Center for Infectious Diseases, Huashan Hospital, Shanghai Medical College, Fudan University, 525 Wulumuqizhong Road, Jing'an District, Shanghai, China

*Correspondence: Qiuqin He, Email: qqhe@fudan.edu.cn

Table of Contents

NMR and HRMS Spectra of Final Compounds..S3

HPLC of Compound **A3**...S26

HPLC of Compound **A5**...S26

NMR and HRMS Spectra for Final Compounds

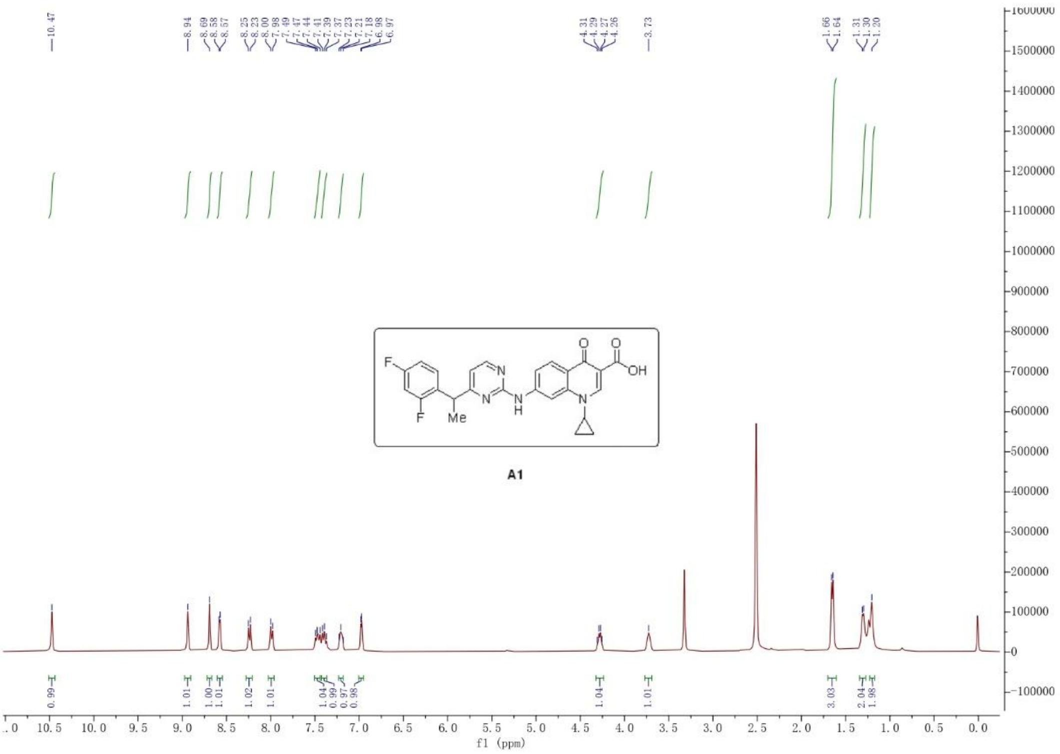

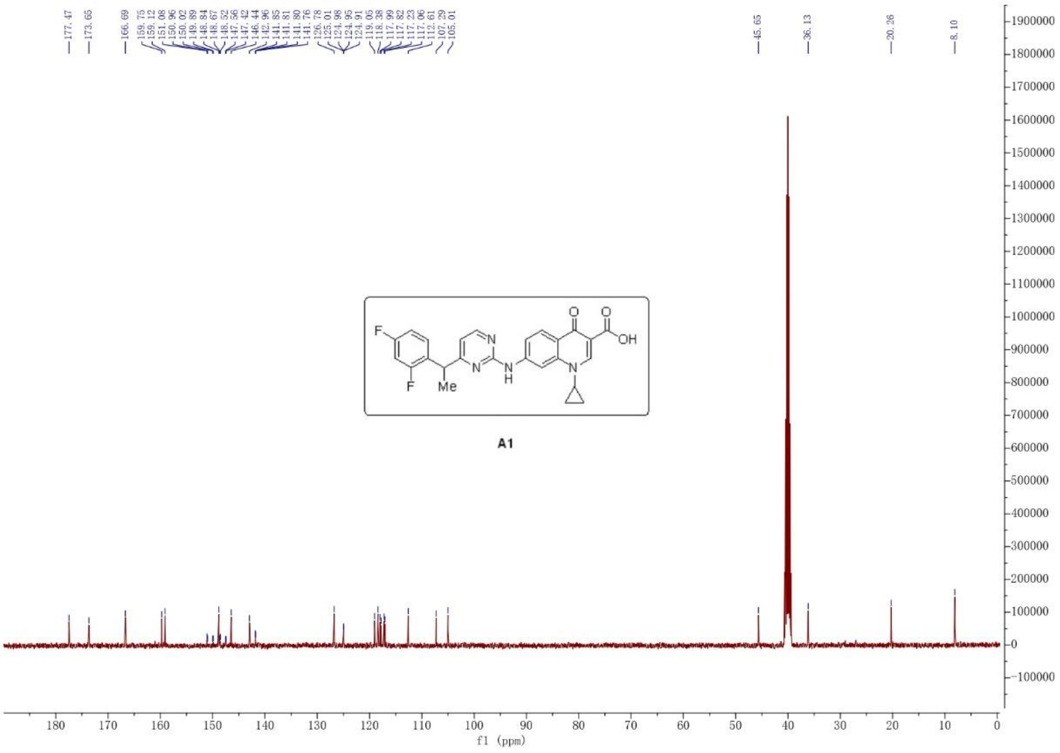

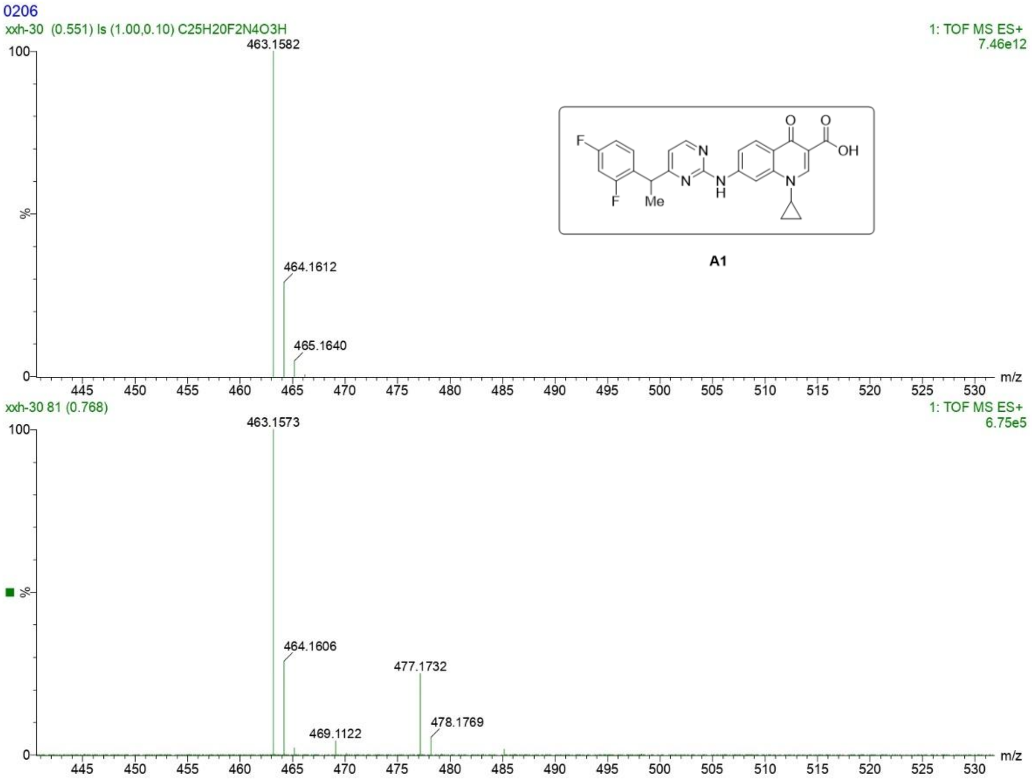
0206
xxh-30 (0.551) Is (1.00,0.10) C25H20F2N4O3H
1: TOF MS ES+
7.46e12
463.1582
464.1612
465.1640
A1
xxh-30 81 (0.768)
1: TOF MS ES+
6.75e5
463.1573
464.1606
477.1732
478.1769
469.1122
m/z

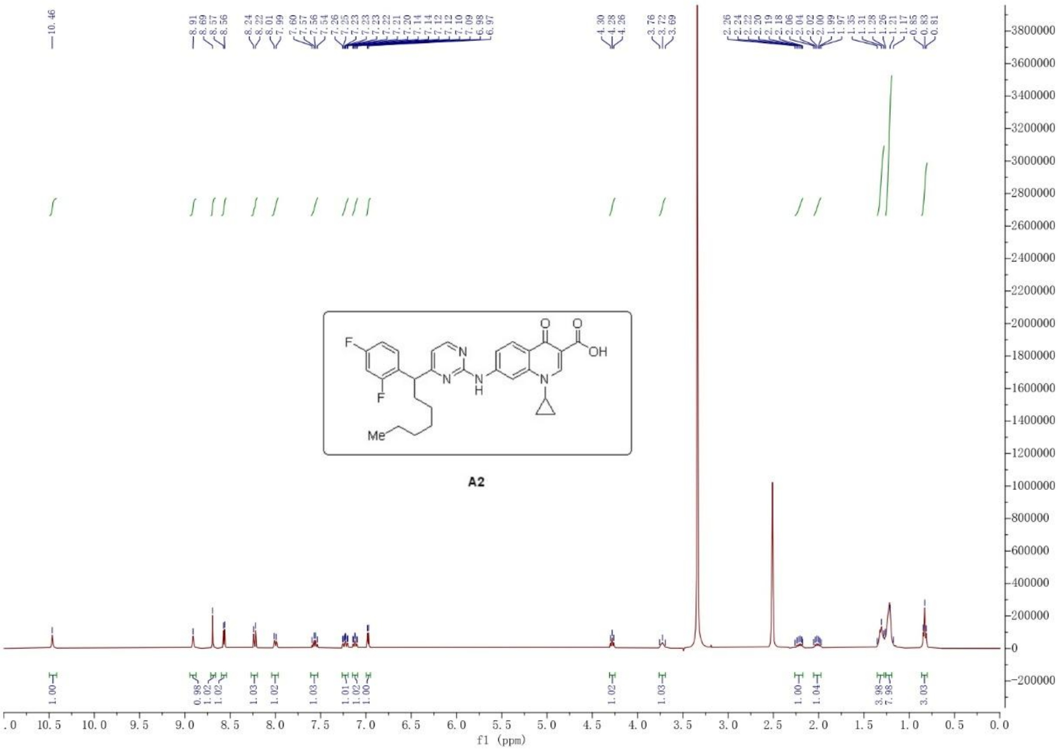
A2
f1 (ppm)

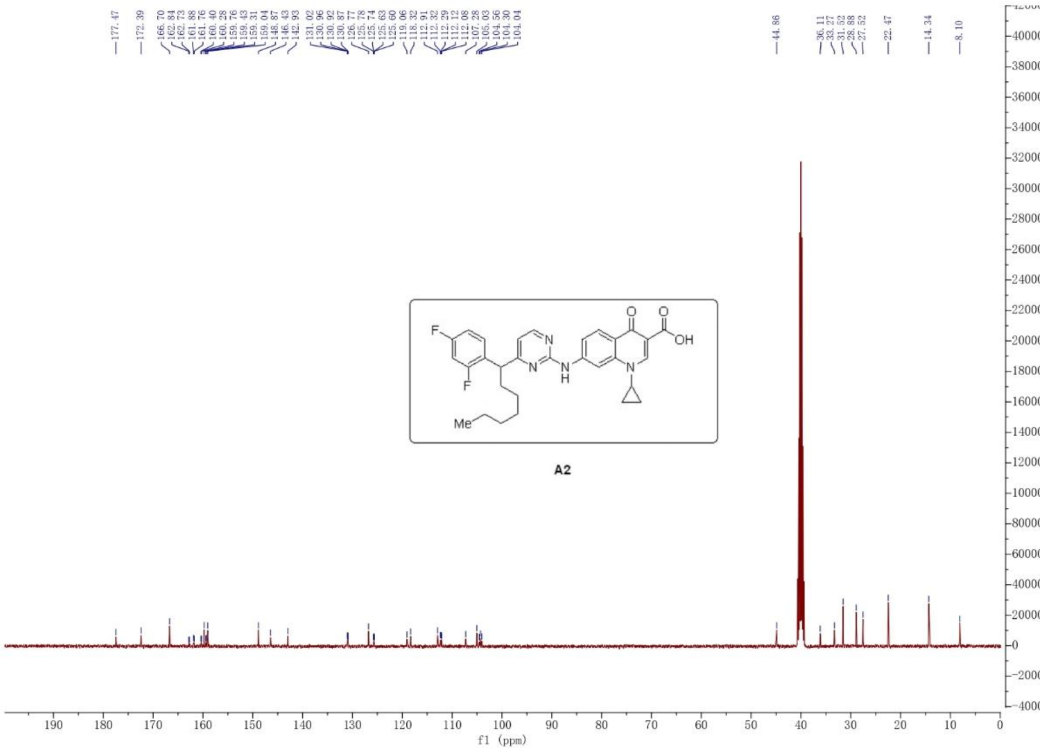
177.47
172.39
166.70
162.84
162.73
161.88
161.76
160.40
160.28
159.76
159.43
159.31
159.04
148.87
146.43
142.93
131.02
130.96
130.92
130.87
126.77
125.78
125.74
125.63
125.60
119.06
118.32
112.91
112.32
112.29
112.12
112.08
107.28
105.03
104.56
104.30
104.04
44.86
36.11
33.27
31.52
28.88
27.52
22.47
14.34
8.10
A2
f1 (ppm)

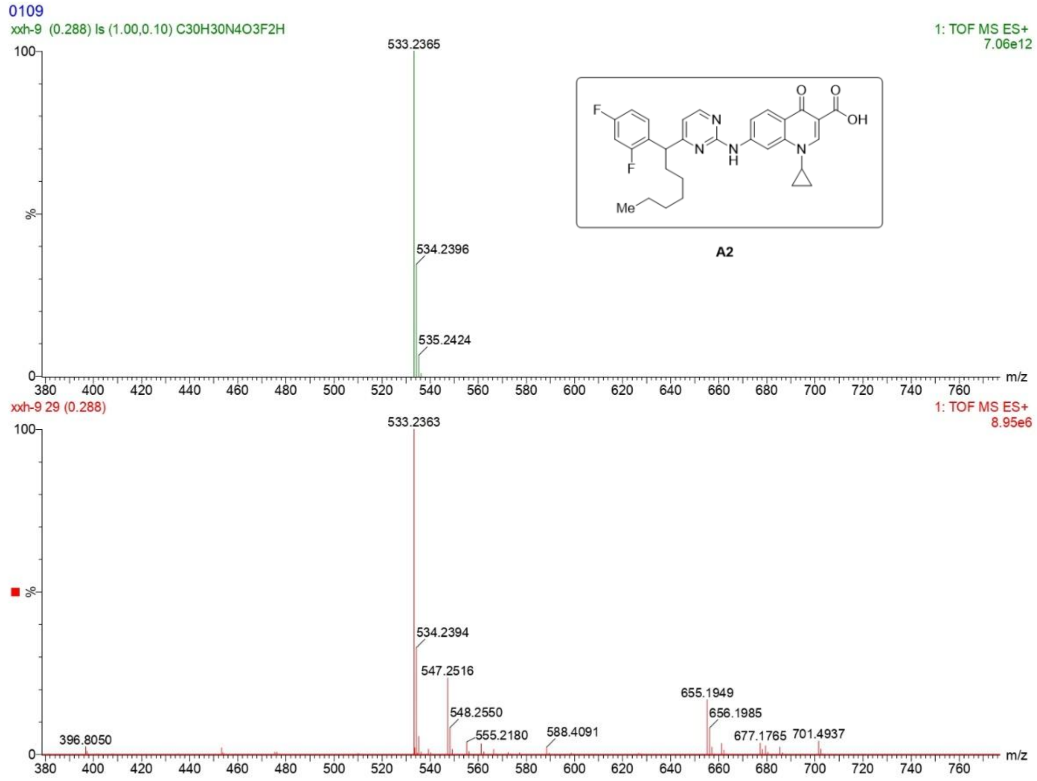
0109
xxh-9 (0.288) Is (1.00,0.10) C30H30N4O3F2H
1: TOF MS ES+
7.06e12
533.2365
534.2396
535.2424
A2
xxh-9 29 (0.288)
1: TOF MS ES+
8.95e6
533.2363
534.2394
547.2516
548.2550
555.2180
588.4091
655.1949
656.1985
677.1765
701.4937
396.8050
m/z

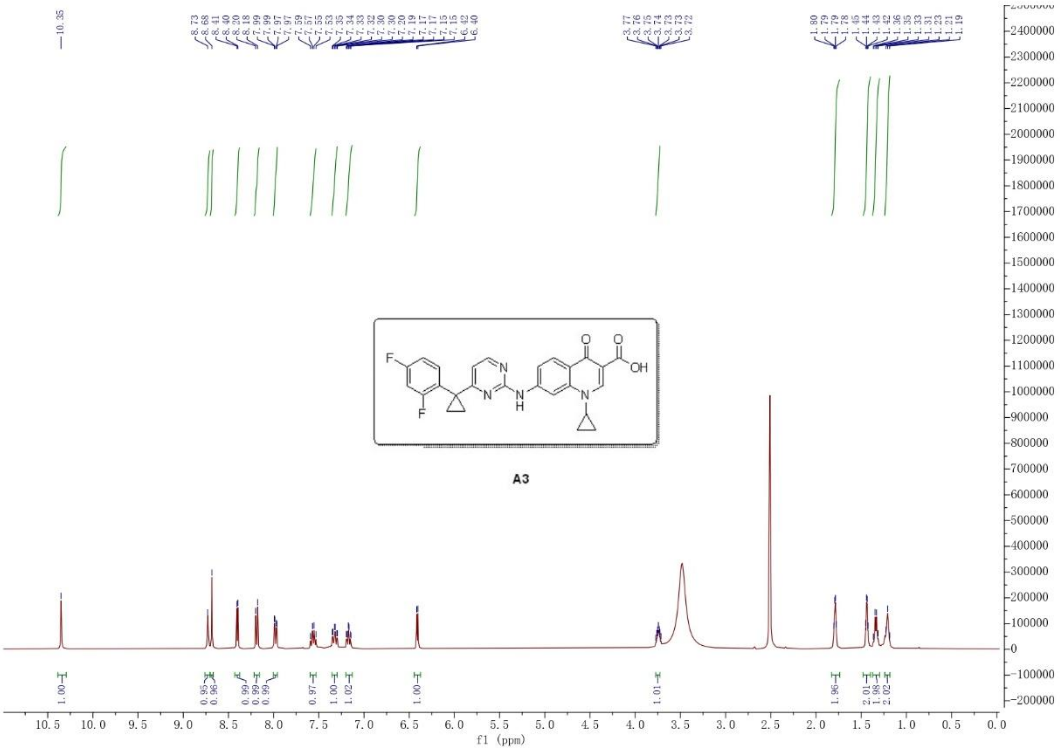
A3
f1 (ppm)

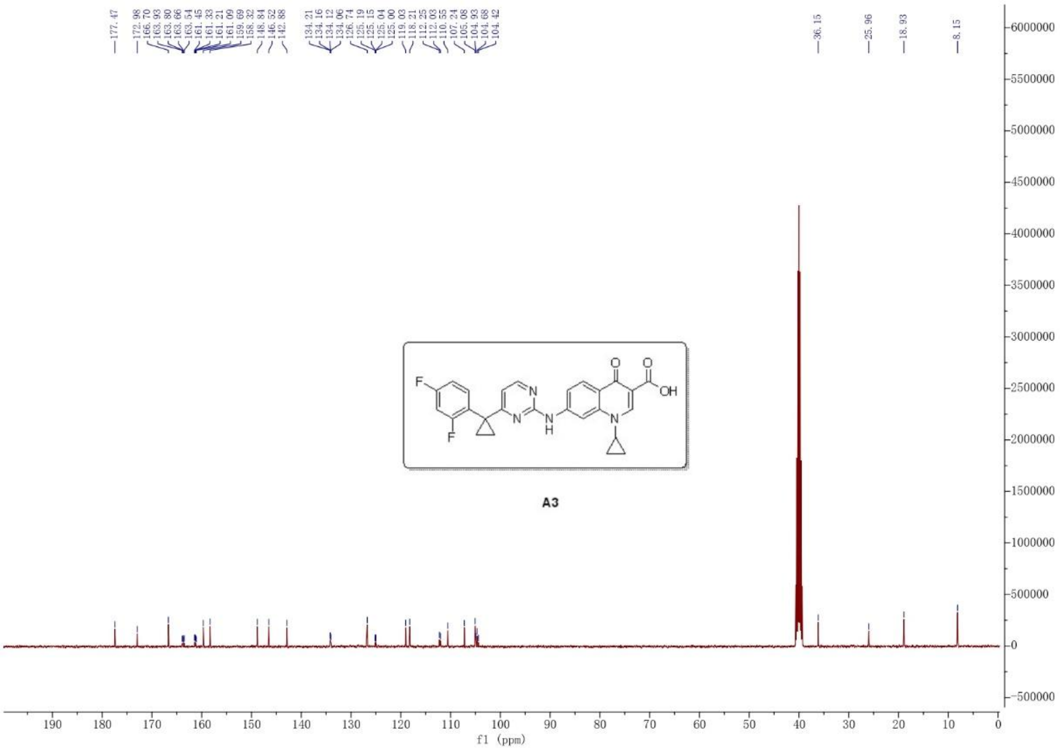
A3
f1 (ppm)

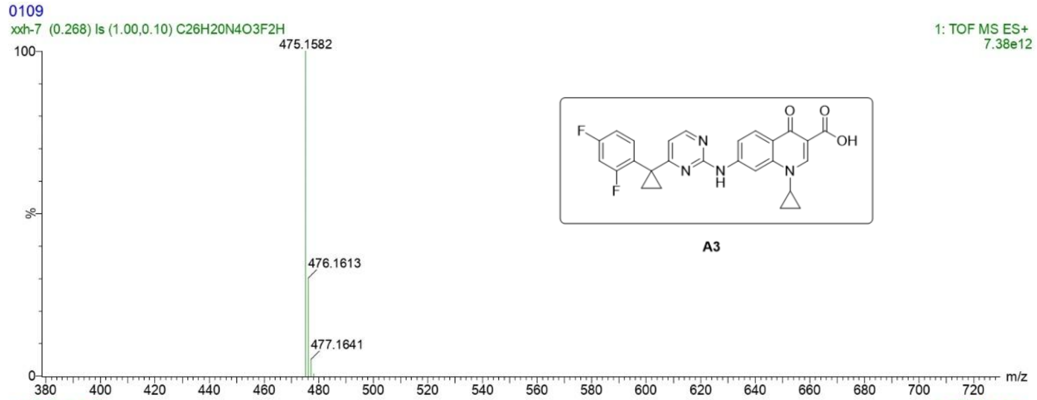
0109
xxh-7 (0.268) Is (1.00,0.10) C26H20N4O3F2H
1: TOF MS ES+
7.38e12
475.1582
476.1613
477.1641
%
m/z
A3

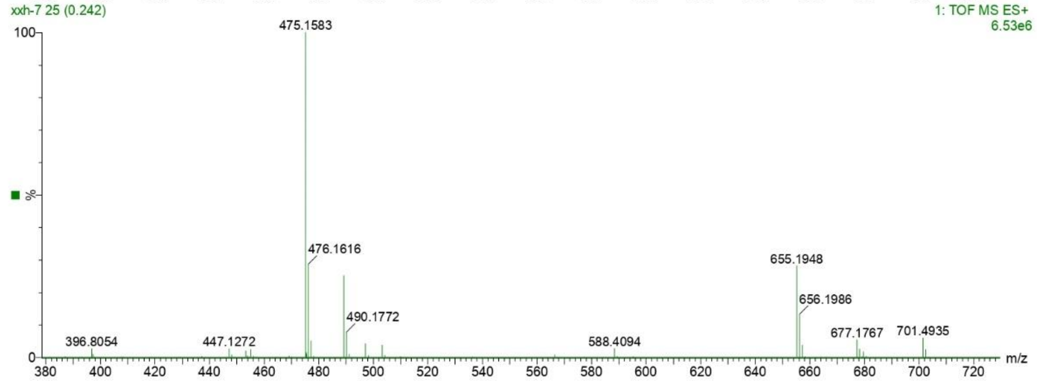
xxh-7 25 (0.242)
1: TOF MS ES+
6.53e6
475.1583
476.1616
490.1772
396.8054
447.1272
588.4094
655.1948
656.1986
677.1767
701.4935
%
m/z

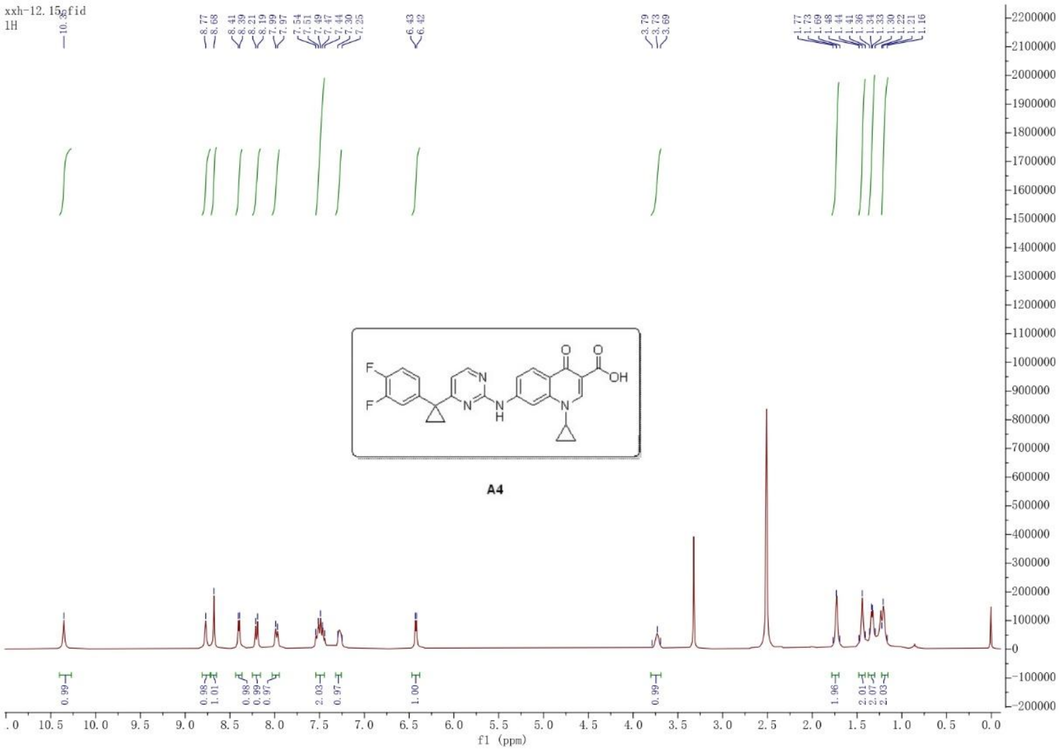
xxh-12.15gfid
1H
A4
f1 (ppm)

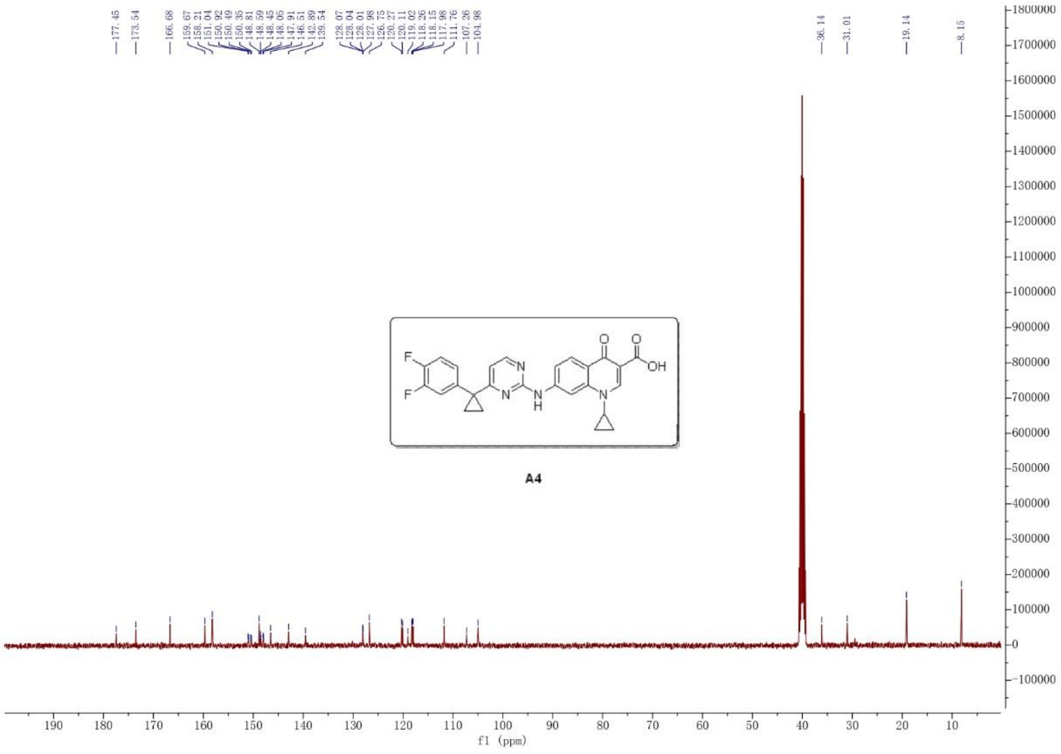
177.45
173.54
166.68
159.67
158.21
151.04
150.92
150.49
150.35
148.81
148.59
148.45
148.05
147.91
146.51
142.89
139.54
128.07
128.04
128.01
127.98
126.75
120.27
120.11
119.02
118.26
118.15
117.98
111.76
107.26
104.98
36.14
31.01
19.14
8.15
A4
f1 (ppm)

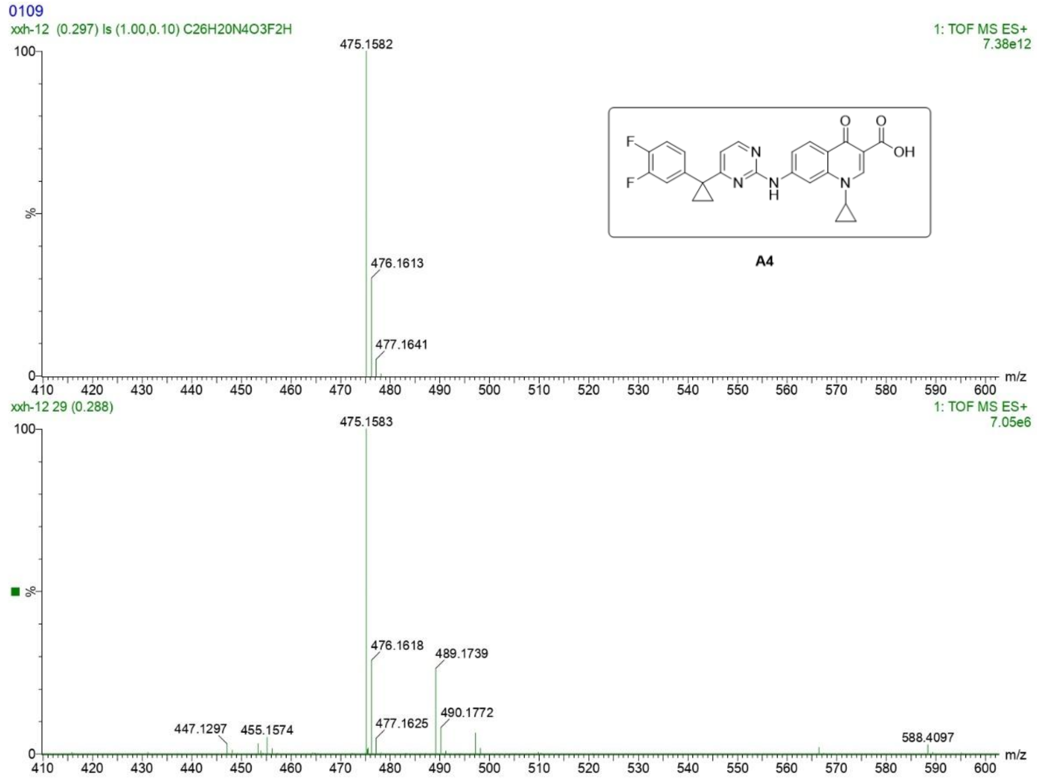
0109
xxh-12 (0.297) Is (1.00,0.10) C26H20N4O3F2H
1: TOF MS ES+
7.38e12
475.1582
476.1613
477.1641
A4
xxh-12 29 (0.288)
1: TOF MS ES+
7.05e6
475.1583
476.1618
489.1739
490.1772
477.1625
447.1297
455.1574
588.4097
m/z

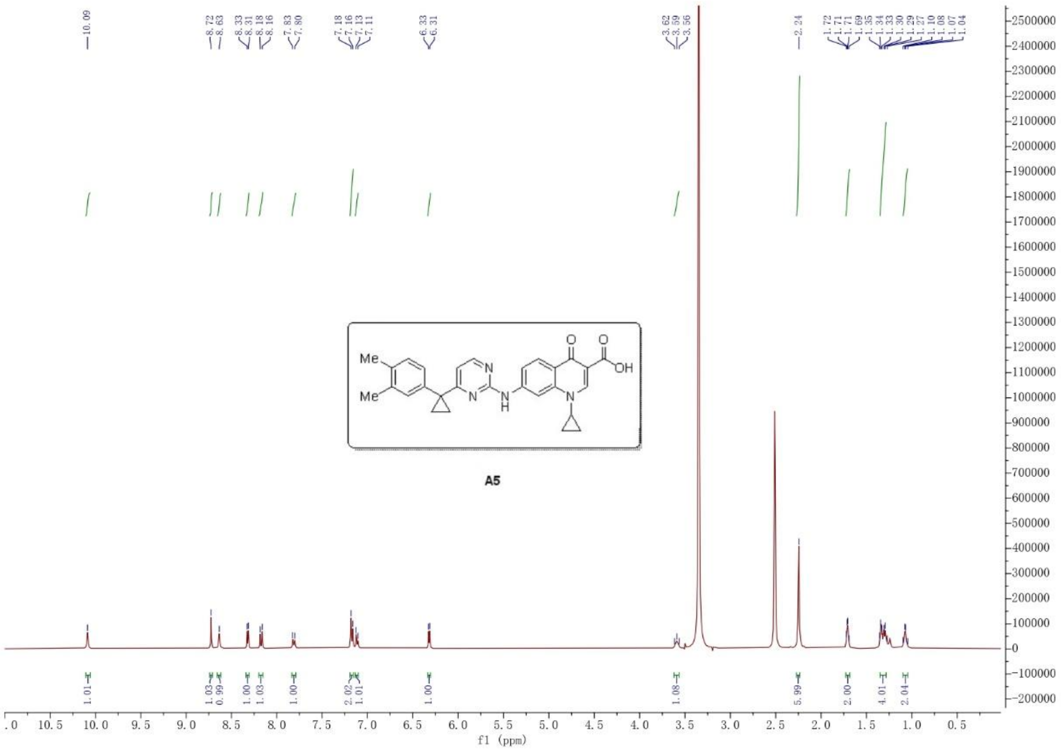

A5

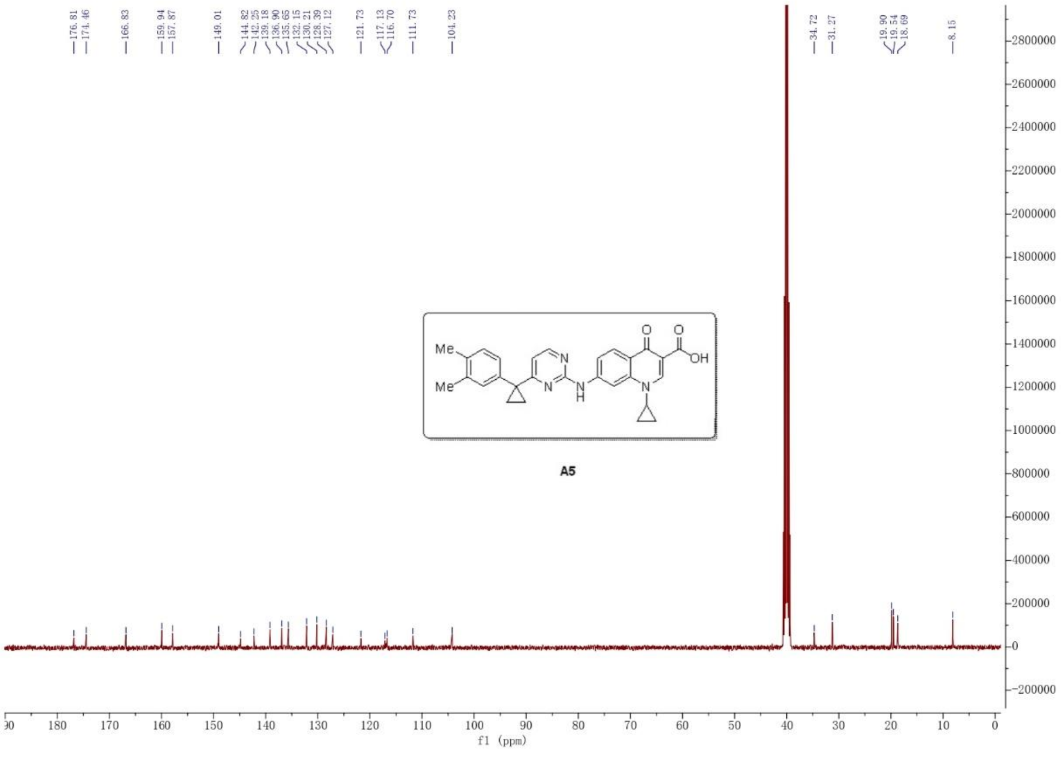

A5

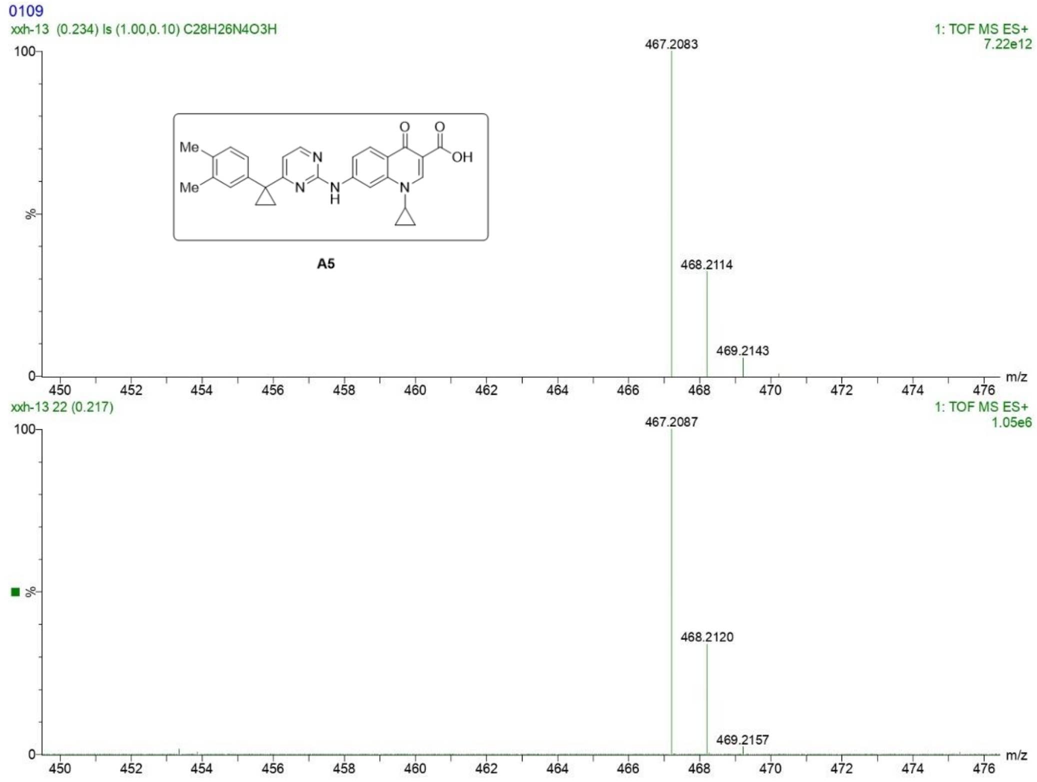
0109
xxh-13 (0.234) Is (1.00,0.10) C28H26N4O3H
1: TOF MS ES+
7.22e12
467.2083
468.2114
469.2143
A5
xxh-13 22 (0.217)
1: TOF MS ES+
1.05e6
467.2087
468.2120
469.2157
m/z
%

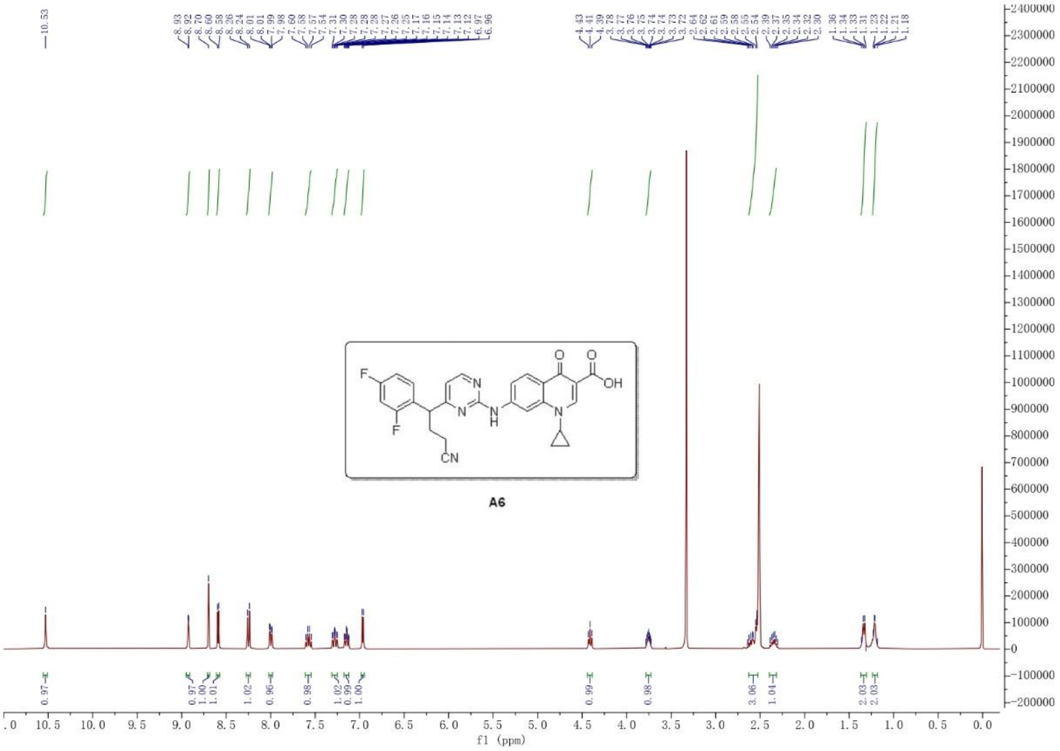
A6
f1 (ppm)

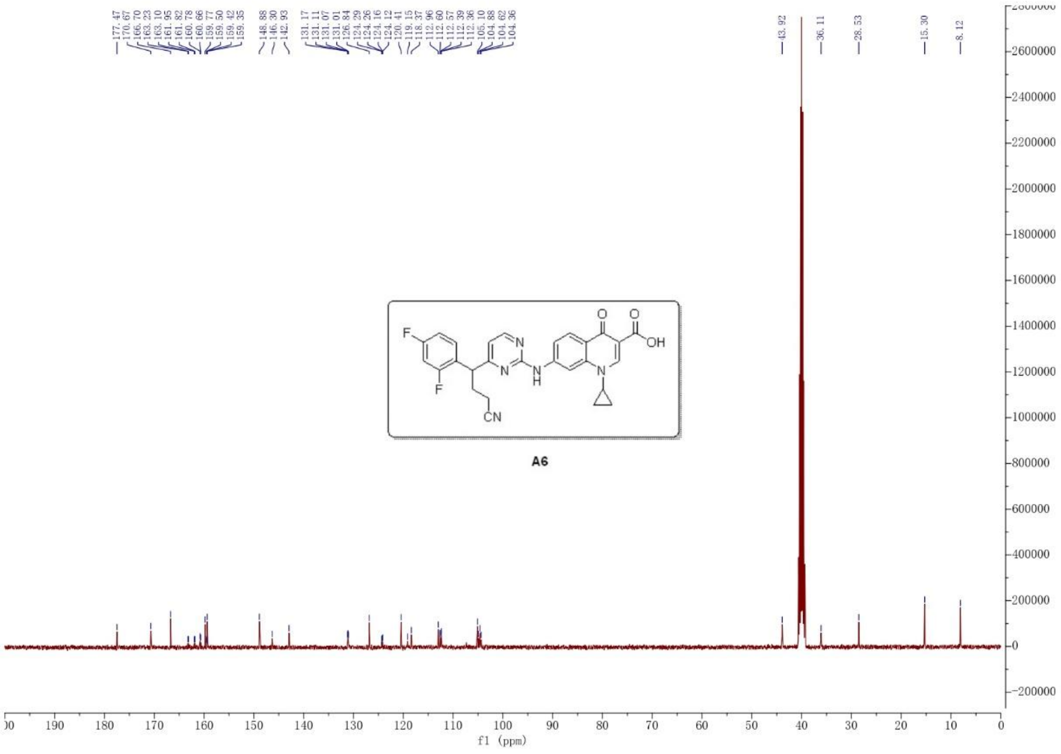
177.47
170.67
166.70
163.23
163.10
161.95
161.82
160.78
160.66
159.77
159.50
159.42
159.35
148.88
146.30
142.93
131.17
131.11
131.07
131.01
126.84
124.29
124.26
124.16
124.12
120.41
119.15
118.37
112.96
112.60
112.57
112.39
112.36
105.10
104.88
104.62
104.36
43.92
36.11
28.33
15.30
8.12
A6
f1 (ppm)

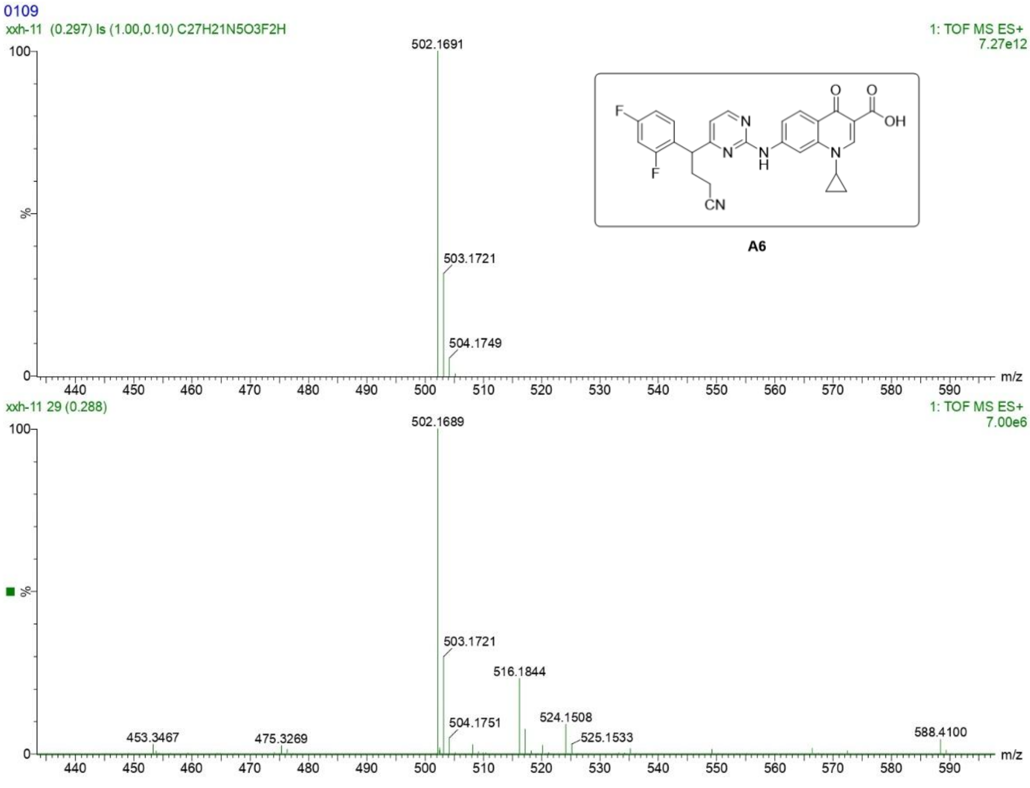
0109
xxh-11 (0.297) Is (1.00,0.10) C27H21N5O3F2H
1: TOF MS ES+
7.27e12
502.1691
503.1721
504.1749
A6
m/z
xxh-11 29 (0.288)
1: TOF MS ES+
7.00e6
502.1689
503.1721
516.1844
524.1508
504.1751
525.1533
453.3467
475.3269
588.4100
m/z

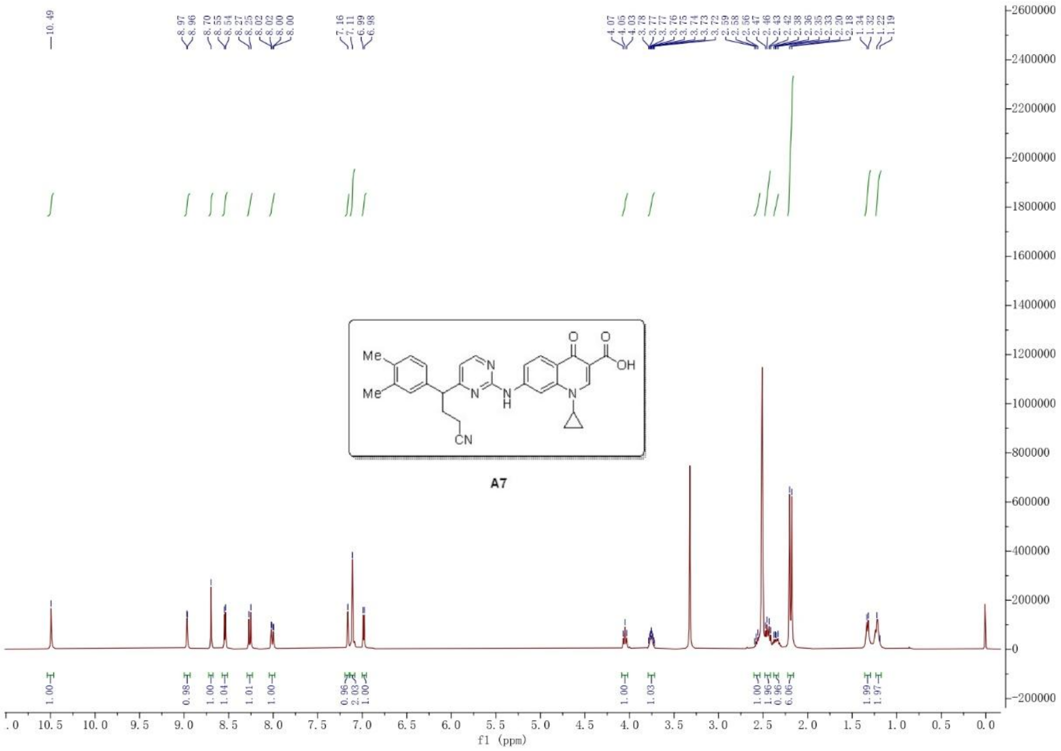

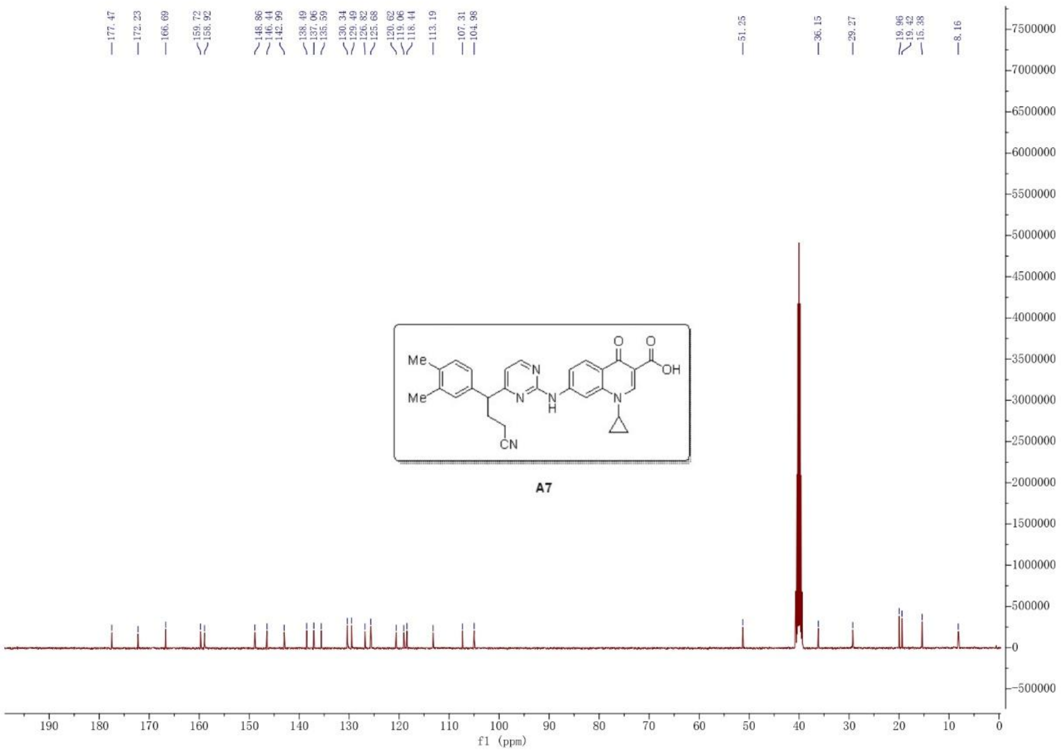

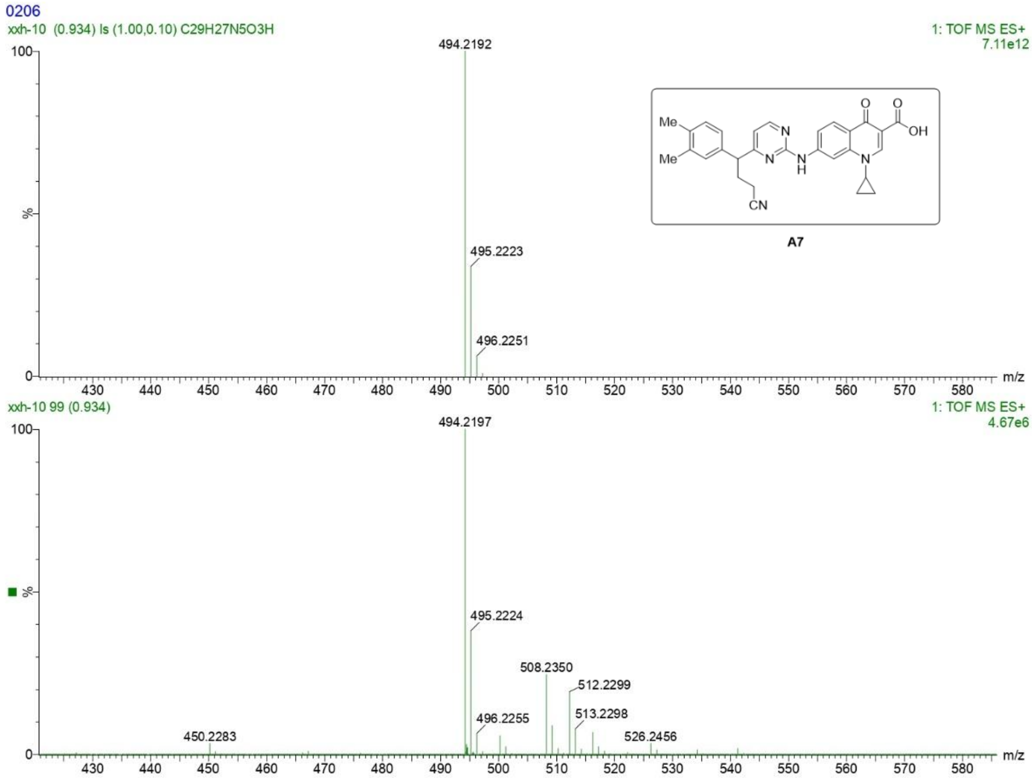
0206
xxh-10 (0.934) Is (1.00,0.10) C29H27N5O3H
1: TOF MS ES+
7.11e12
494.2192
495.2223
496.2251
xxh-10 99 (0.934)
1: TOF MS ES+
4.67e6
494.2197
495.2224
508.2350
512.2299
513.2298
496.2255
526.2456
450.2283
m/z
Me
Me
CN
N
N
H
O
O
OH
N

A7

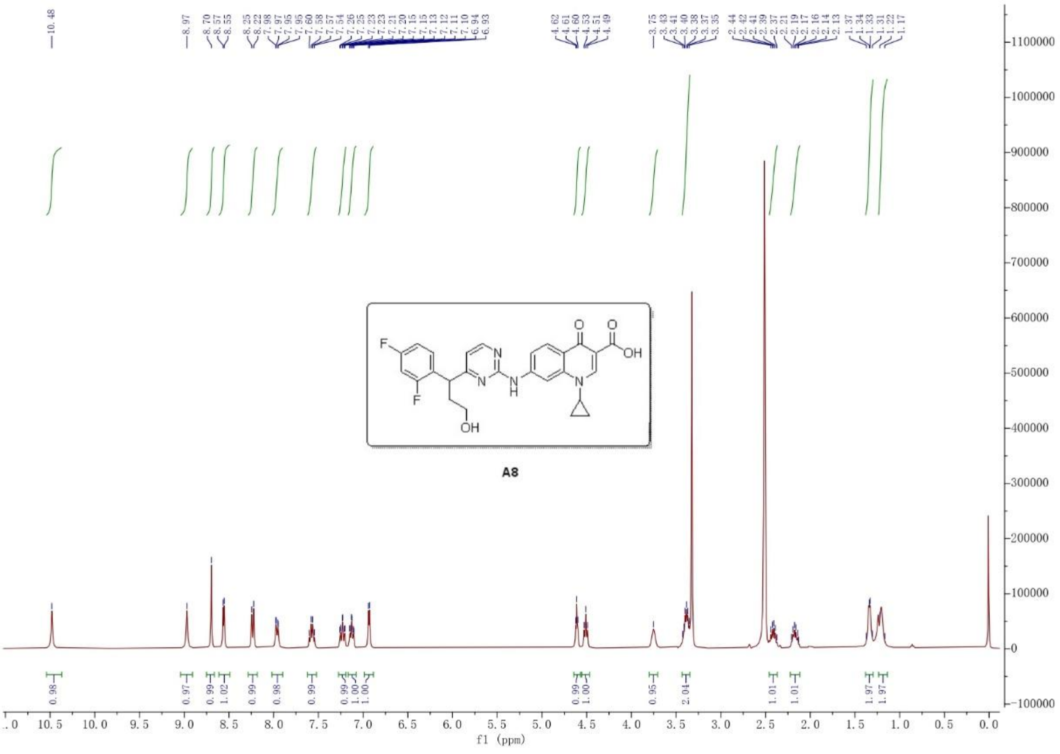
F
F
OH
N
N
H
O
O
OH
N
f1 (ppm)

A8

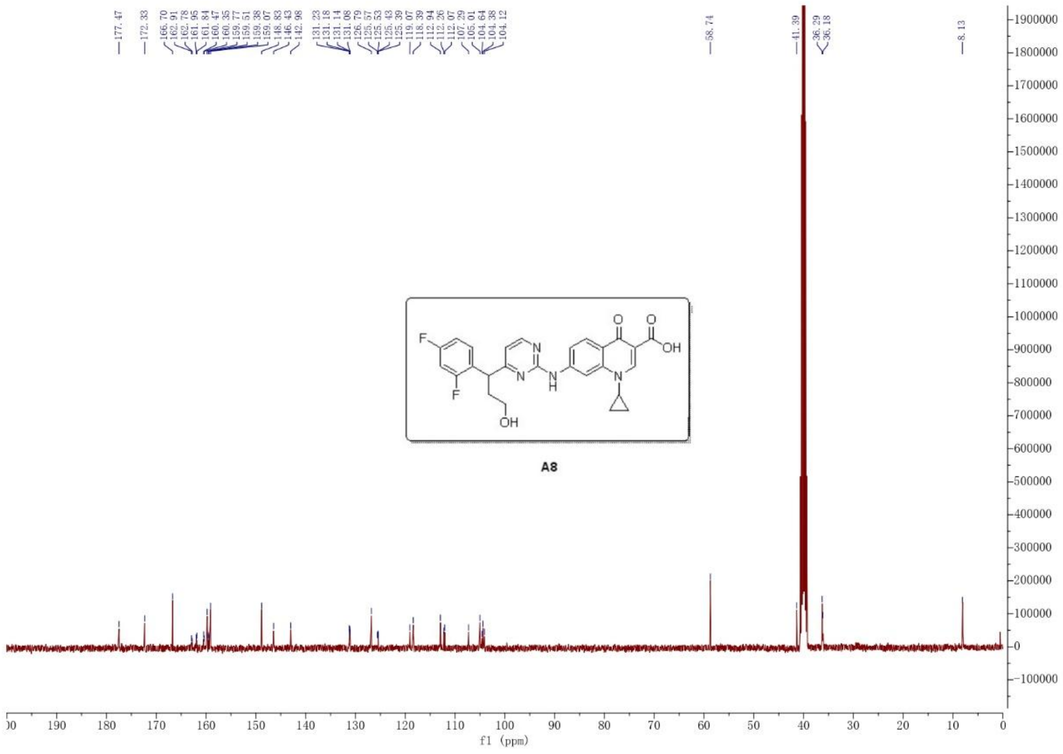
177.47
172.33
166.70
162.91
162.78
161.95
161.84
160.47
160.35
159.77
159.51
159.38
159.07
148.83
146.43
142.98
131.23
131.18
131.14
131.08
126.79
125.57
125.53
125.43
125.39
119.07
118.39
112.94
112.26
112.07
107.29
105.01
104.64
104.38
104.12
58.74
41.39
36.29
36.18
8.13
A8
f1 (ppm)

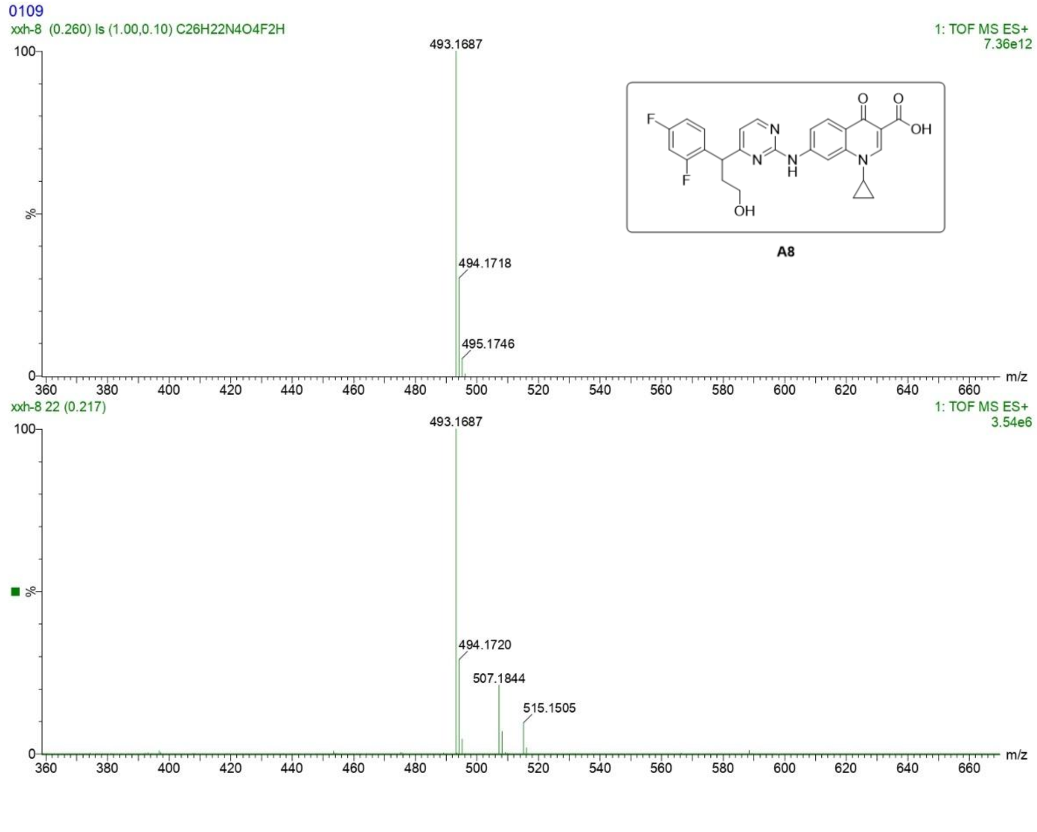
0109
xxh-8 (0.260) Is (1.00,0.10) C26H22N4O4F2H
1: TOF MS ES+
7.36e12
493.1687
494.1718
495.1746
A8
m/z
xxh-8 22 (0.217)
1: TOF MS ES+
3.54e6
493.1687
494.1720
507.1844
515.1505
m/z

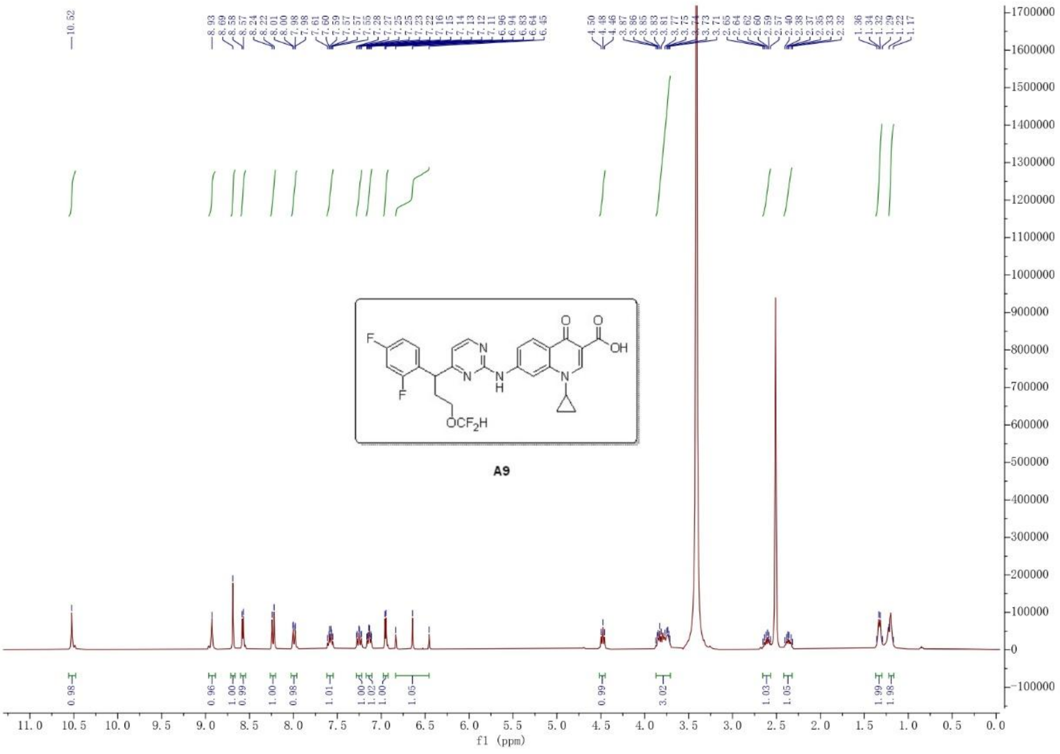
10.52
8.93 8.69 8.58 8.57 8.24 8.22 8.01 8.00 7.98 7.98 7.61 7.60 7.59 7.57 7.57 7.55 7.28 7.27 7.25 7.25 7.23 7.22 7.16 7.15 7.14 7.13 7.12 7.11 6.96 6.94 6.83 6.64 6.45
4.50 4.48 4.46 3.87 3.86 3.85 3.83 3.81 3.77 3.75 3.74 3.73 3.71 2.65 2.64 2.62 2.60 2.59 2.57 2.40 2.38 2.37 2.35 2.33 2.32
1.36 1.34 1.32 1.29 1.22 1.17
0.98 0.96 1.00 0.99 1.00 0.98 1.01 1.00 1.02 1.00 1.05 0.99 3.02 1.03 1.05 1.99 1.98
F
F
N
N
N
H
O
O
OH
N
OCF2H
A9
f1 (ppm)

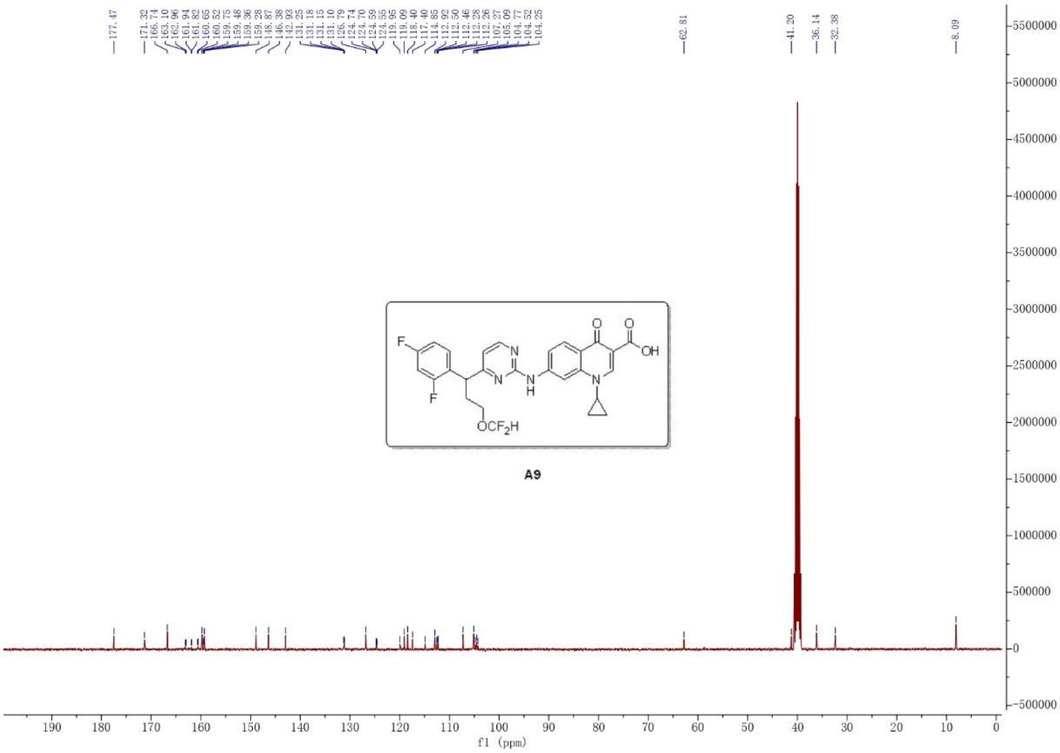
177.47
171.32 166.74 163.10 162.96 161.94 161.82 160.65 160.52 159.75 159.48 159.36 159.28 148.87 146.38 142.93 131.25 131.18 131.15 131.10 126.79 124.74 124.70 124.59 124.55 119.95 119.09 118.40 117.40 114.85 112.92 112.50 112.46 112.38 112.36 107.27 105.09 104.77 104.52 104.25
62.81
41.20 36.14 32.38
8.09
F
F
N
N
N
H
O
O
OH
N
OCF2H
A9
f1 (ppm)

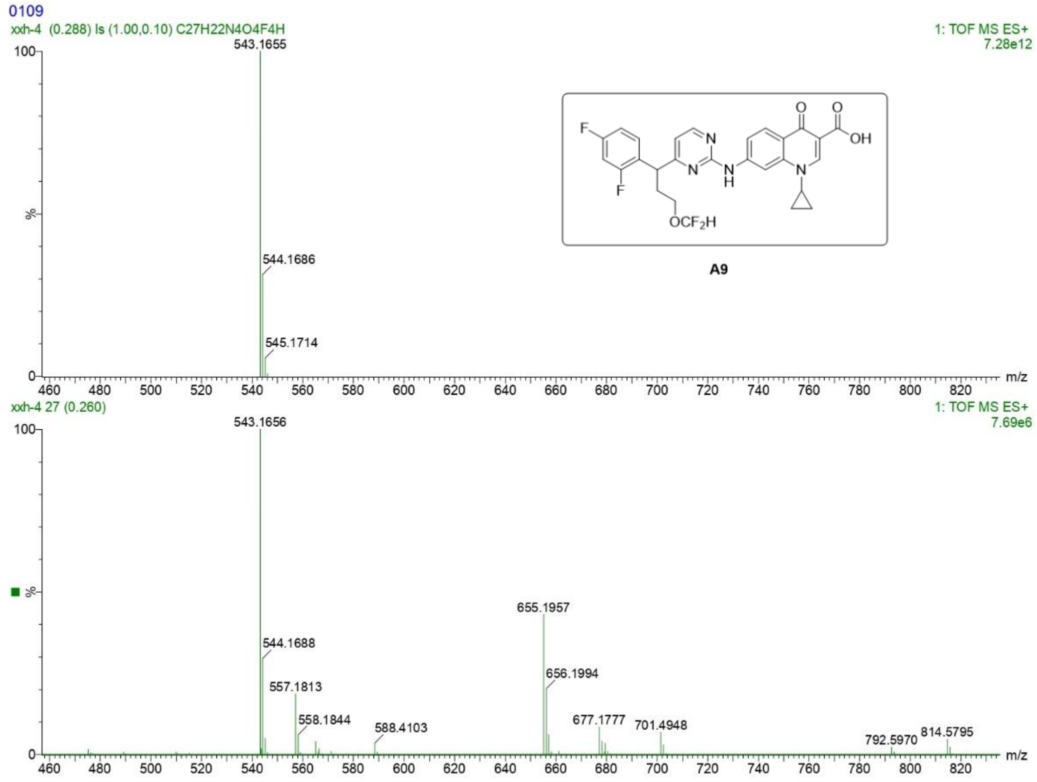
0109
xxh-4 (0.288) Is (1.00,0.10) C27H22N4O4F4H
1: TOF MS ES+
7.28e12
543.1655
544.1686
545.1714
A9
xxh-4 27 (0.260)
1: TOF MS ES+
7.69e6
543.1656
544.1688
557.1813
558.1844
588.4103
655.1957
656.1994
677.1777
701.4948
792.5970
814.5795
m/z

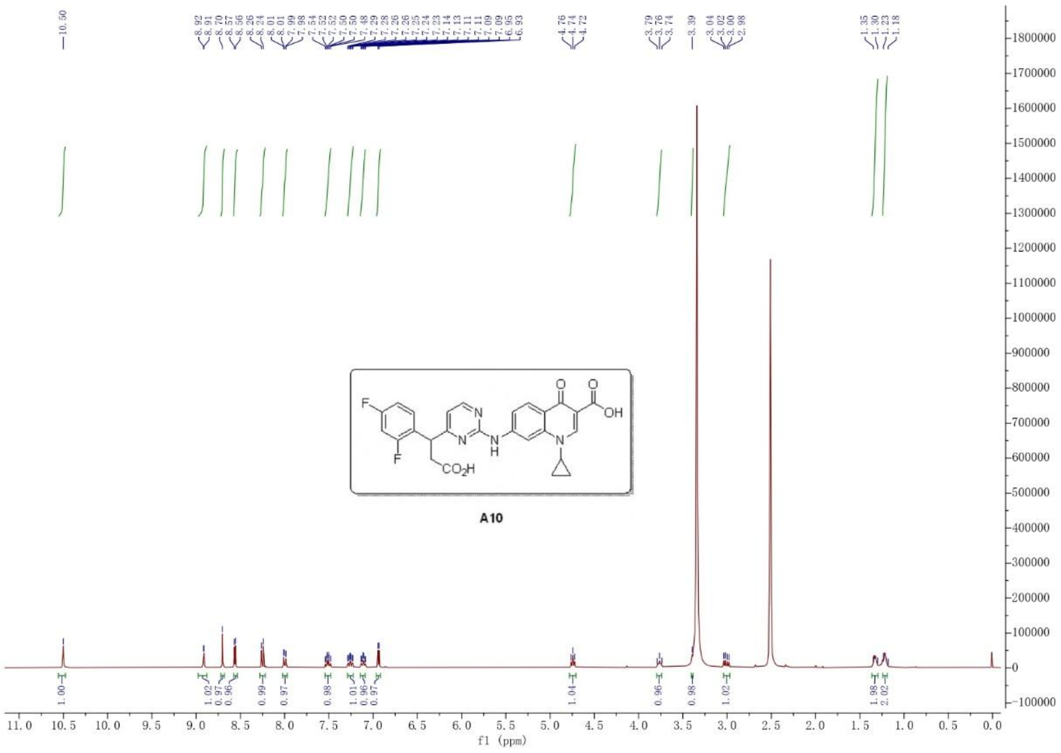
A10
f1 (ppm)

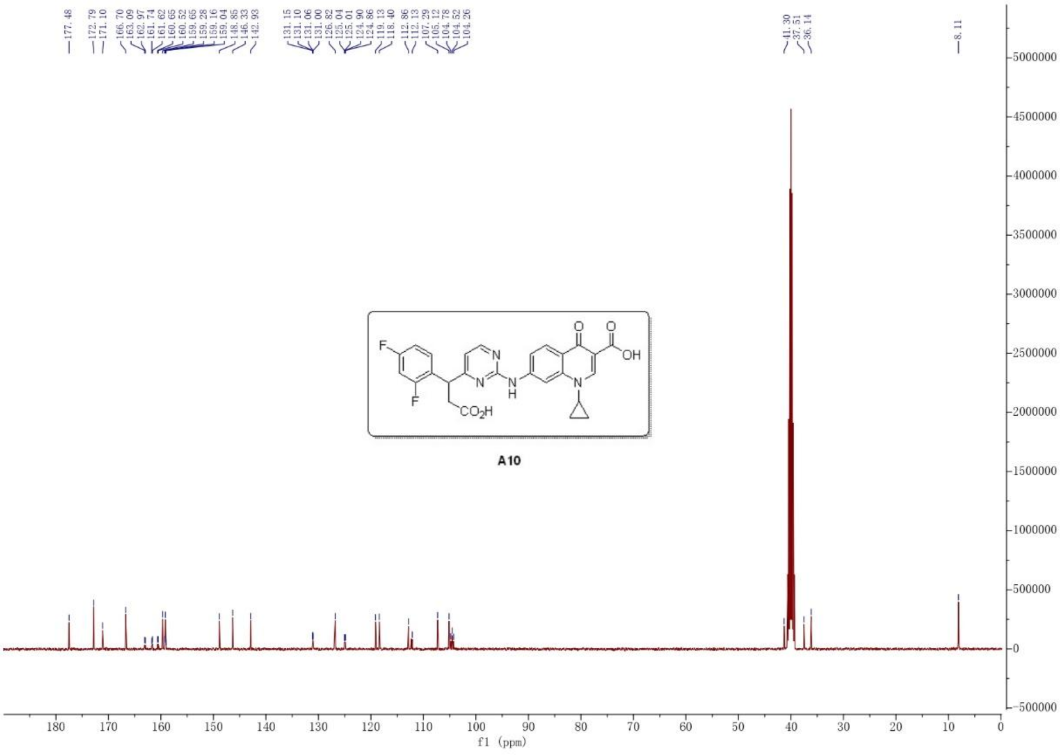
177.48
172.79
171.10
166.70
163.09
162.97
161.74
161.62
160.65
160.52
159.65
159.28
159.16
159.04
148.85
146.33
142.93
131.15
131.10
131.06
131.00
126.82
125.04
125.01
124.90
124.86
119.13
118.40
112.86
112.13
107.29
105.12
104.78
104.52
104.26
41.30
37.51
36.14
8.11
A10
f1 (ppm)

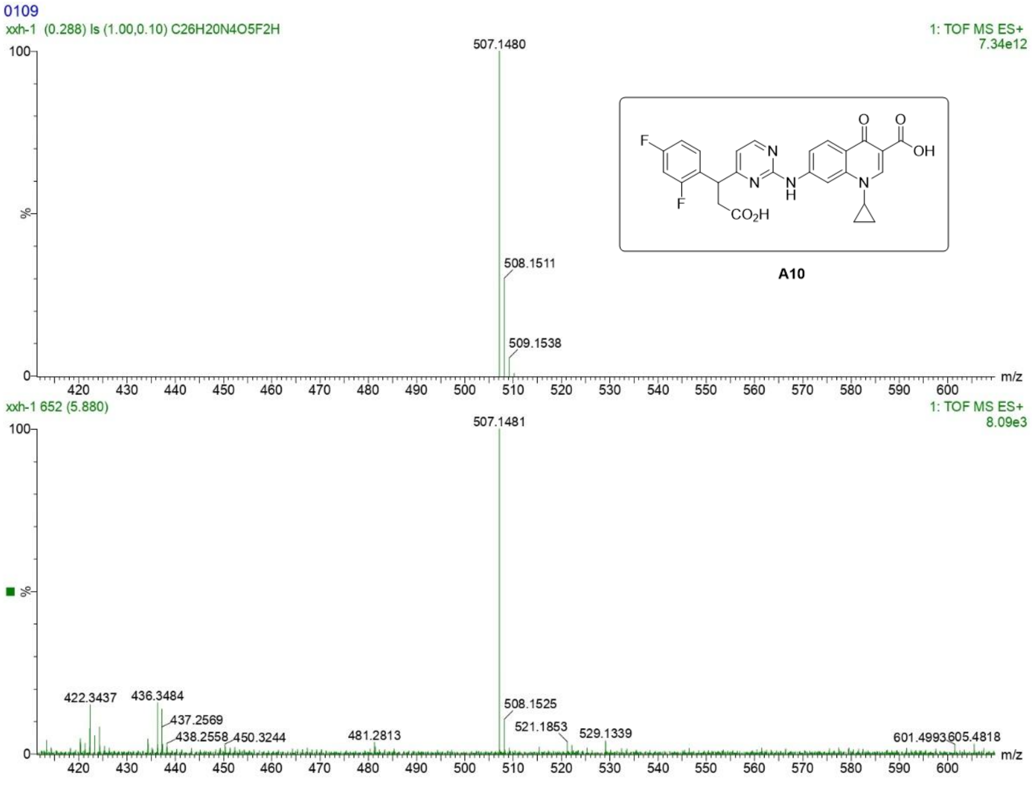
0109
xxh-1 (0.288) Is (1.00,0.10) C26H20N4O5F2H
1: TOF MS ES+
7.34e12
507.1480
508.1511
509.1538
A10
xxh-1 652 (5.880)
1: TOF MS ES+
8.09e3
507.1481
422.3437
436.3484
437.2569
438.2558
450.3244
481.2813
508.1525
521.1853
529.1339
601.4993
605.4818
m/z

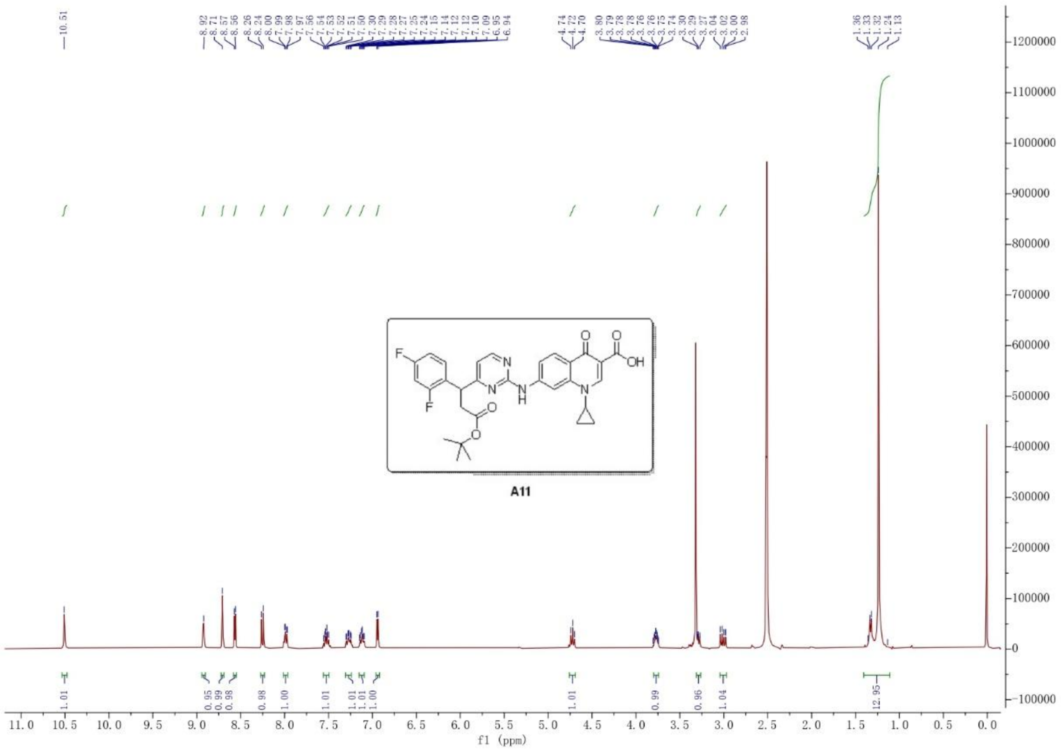
A11

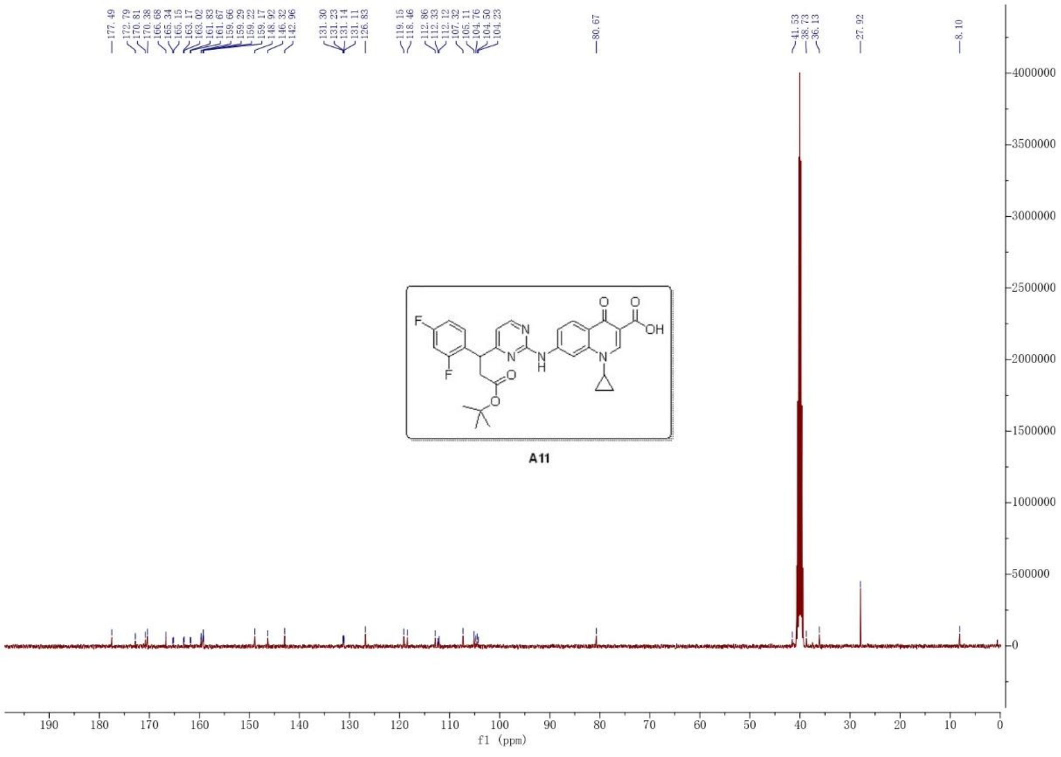
A11

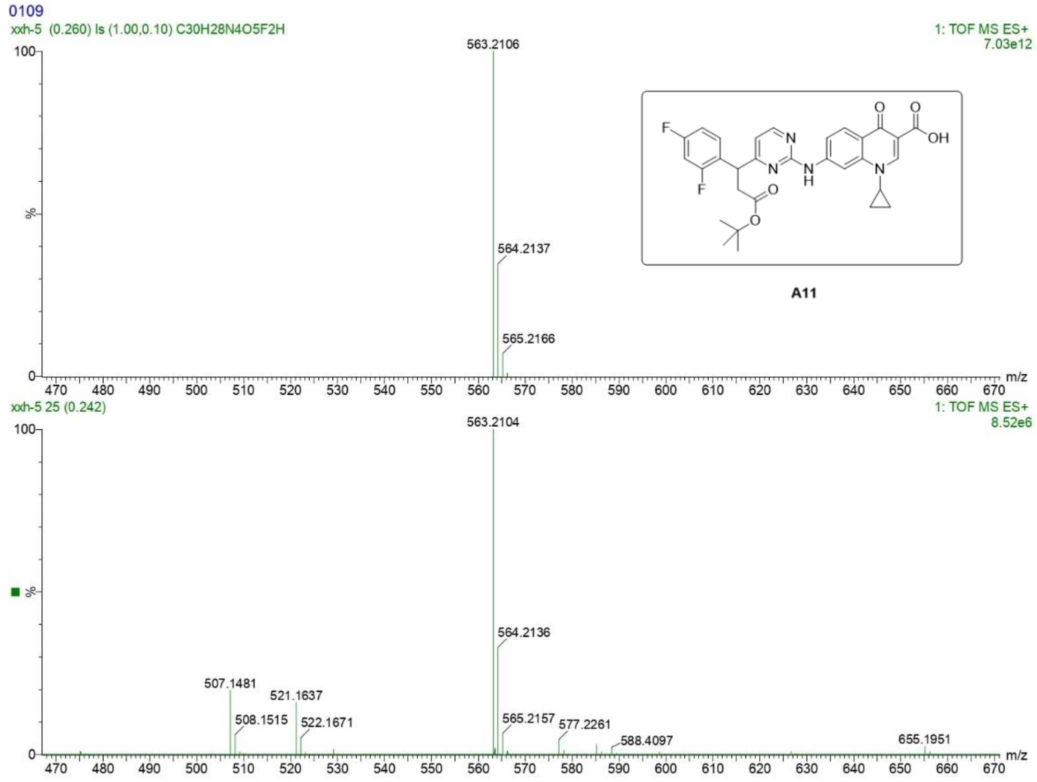
0109
xxh-5 (0.260) Is (1.00,0.10) C30H28N4O5F2H
1: TOF MS ES+
7.03e12
563.2106
564.2137
565.2166
xxh-5 25 (0.242)
1: TOF MS ES+
8.52e6
563.2104
564.2136
507.1481
521.1637
508.1515
522.1671
565.2157
577.2261
588.4097
655.1951
m/z
%

A11

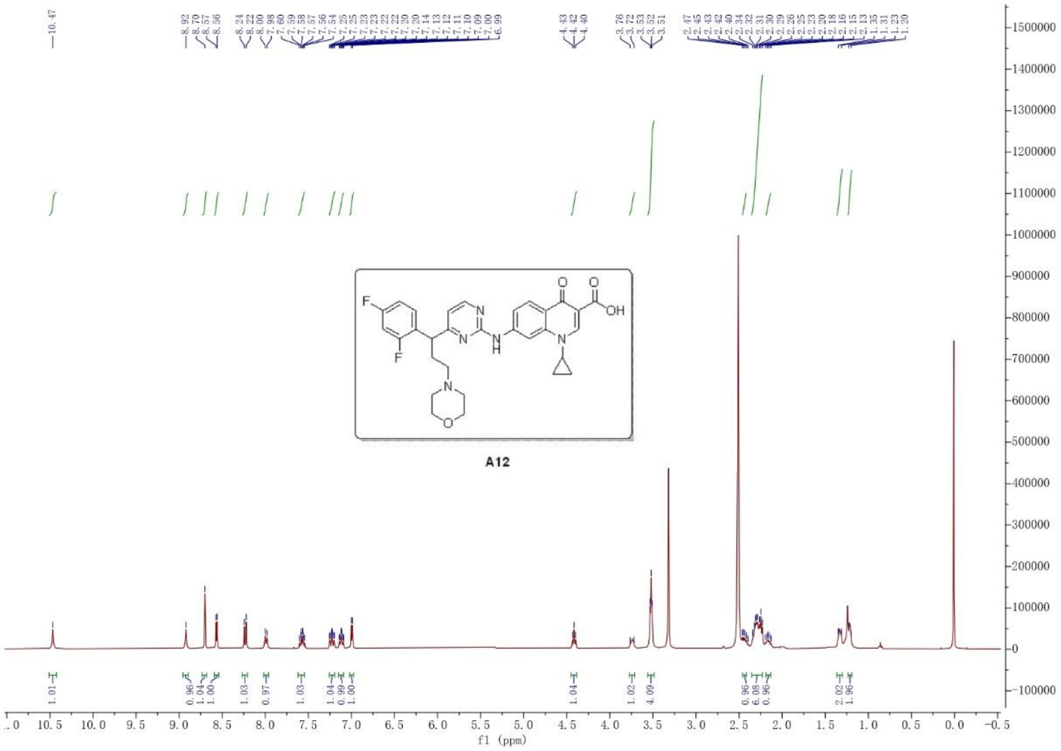
f1 (ppm)

A12

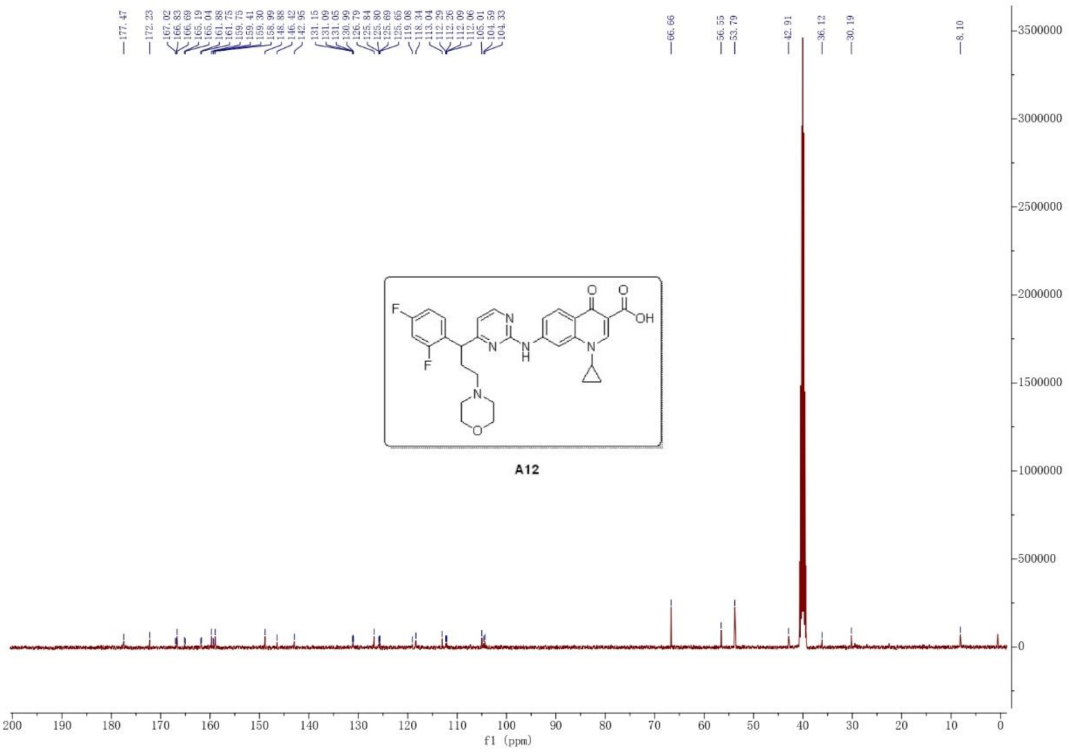
177.47
172.23
167.02
166.83
166.69
165.19
165.04
161.88
161.75
159.75
159.41
159.30
158.99
148.88
146.42
142.95
131.15
131.09
131.05
130.99
126.79
125.84
125.80
125.69
125.65
119.08
118.34
113.04
112.29
112.26
112.09
112.06
105.01
104.59
104.33
66.66
56.55
53.79
42.91
36.12
30.19
8.10
A12
f1 (ppm)

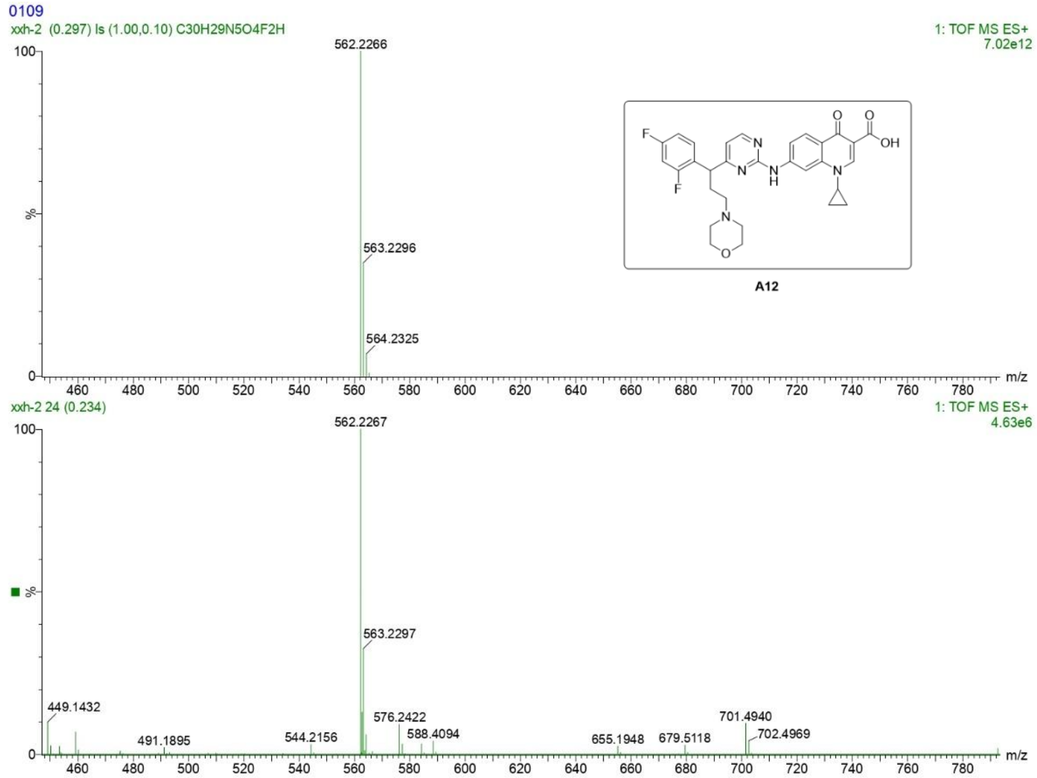
0109
xxh-2 (0.297) Is (1.00,0.10) C30H29N5O4F2H
1: TOF MS ES+
7.02e12
562.2266
563.2296
564.2325
A12
m/z
xxh-2 24 (0.234)
1: TOF MS ES+
4.63e6
562.2267
563.2297
449.1432
491.1895
544.2156
576.2422
588.4094
655.1948
679.5118
701.4940
702.4969
m/z

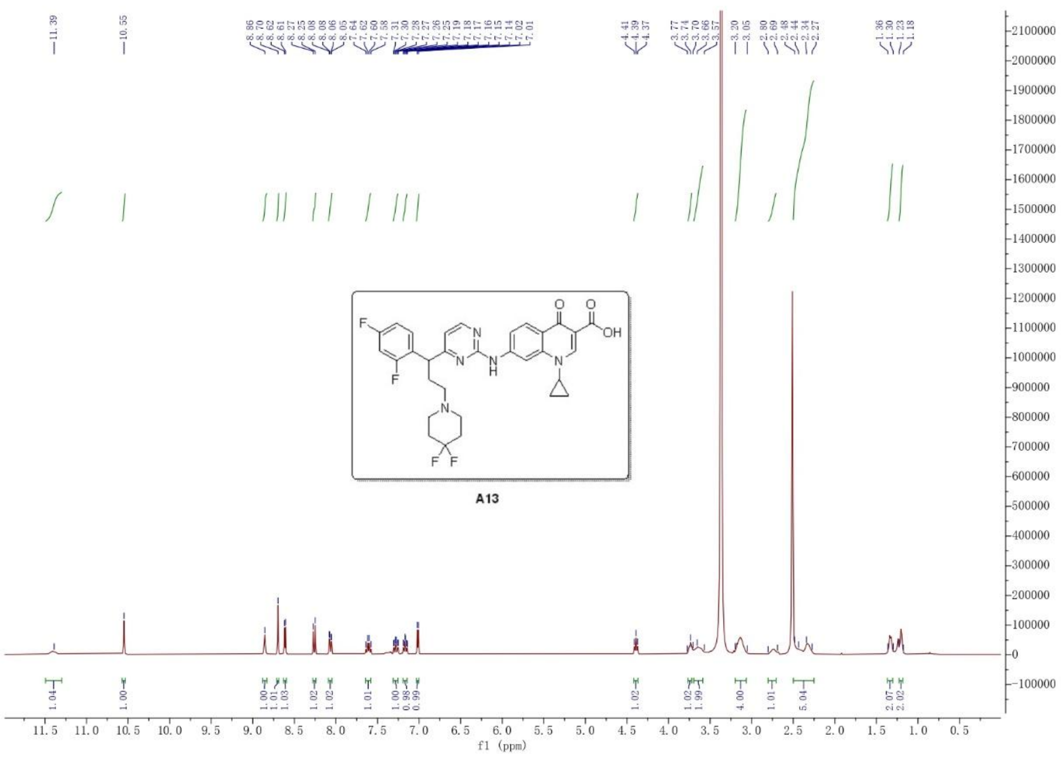

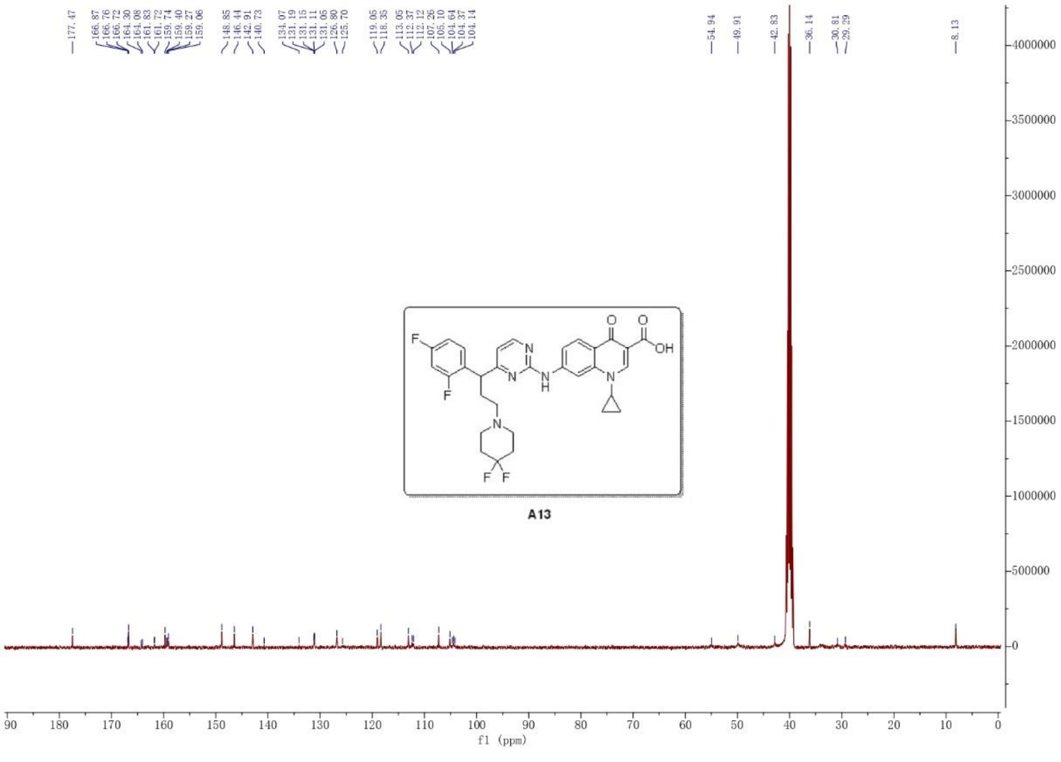

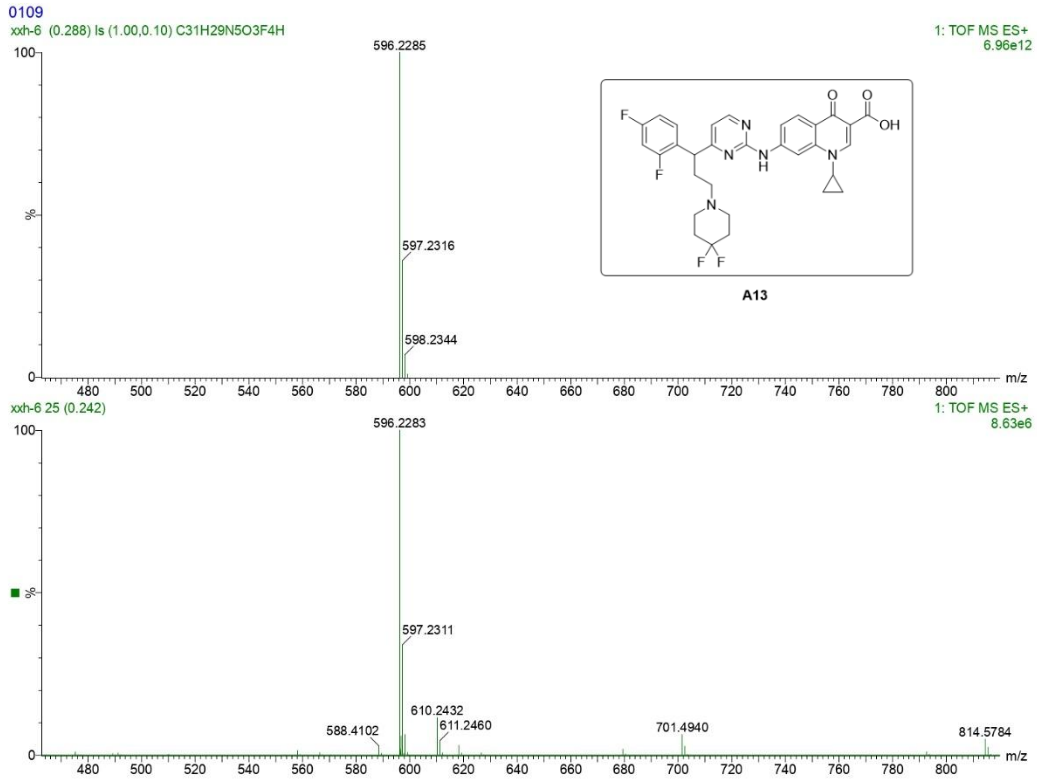
0109
xxh-6 (0.288) Is (1.00,0.10) C31H29N5O3F4H
1: TOF MS ES+
6.96e12
596.2285
597.2316
598.2344
A13
xxh-6 25 (0.242)
1: TOF MS ES+
8.63e6
596.2283
597.2311
610.2432
611.2460
588.4102
701.4940
814.5784
m/z

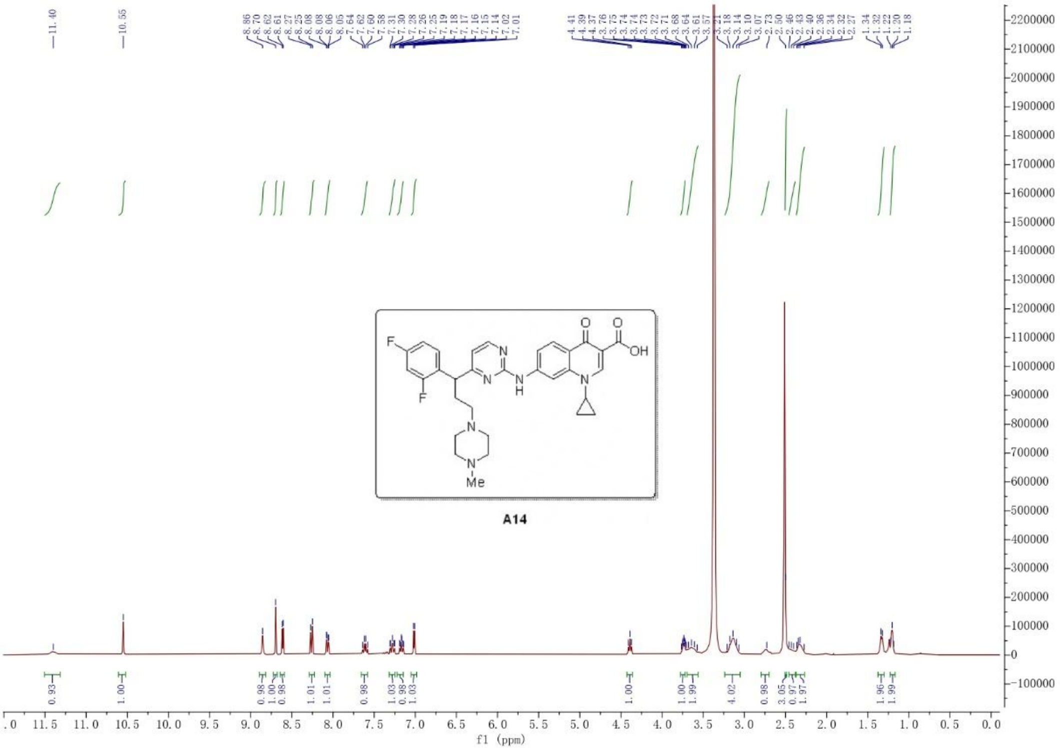
A14
f1 (ppm)

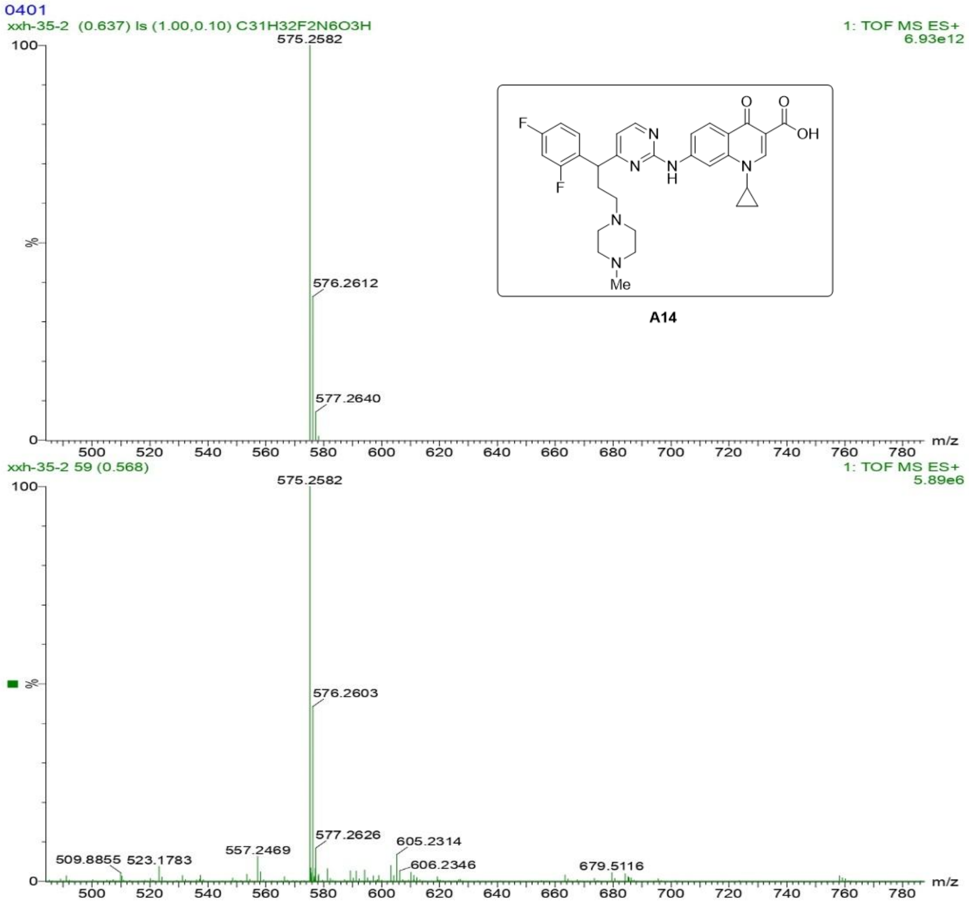
0401
xxh-35-2 (0.637) Is (1.00,0.10) C31H32F2N6O3H
1: TOF MS ES+
6.93e12
575.2582
576.2612
577.2640
xxh-35-2 59 (0.568)
1: TOF MS ES+
5.89e6
575.2582
576.2603
509.8855
523.1783
557.2469
577.2626
605.2314
606.2346
679.5116
m/z
%
A14

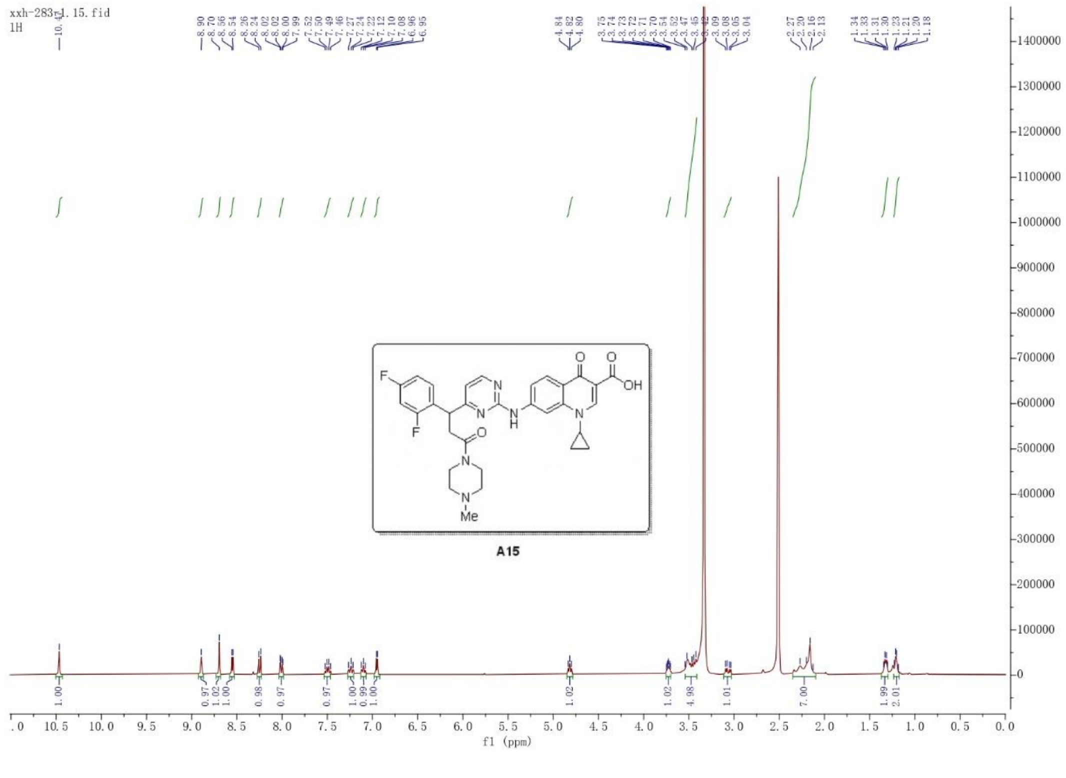
xxh-283-1.15.fid
1H
f1 (ppm)
A15

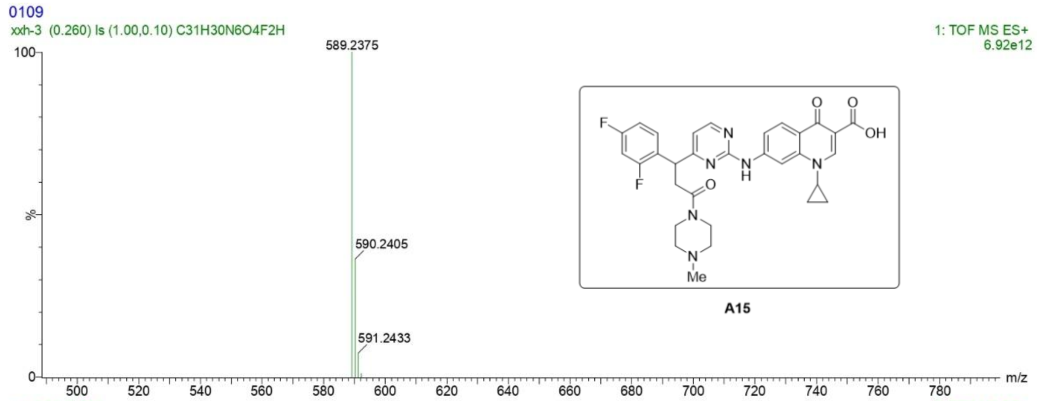
0109
xxh-3 (0.260) Is (1.00,0.10) C31H30N6O4F2H
1: TOF MS ES+
6.92e12
589.2375
590.2405
591.2433
%
m/z
F
F
N
N
N
H
O
OH
O
N
O
N
N
Me
A15

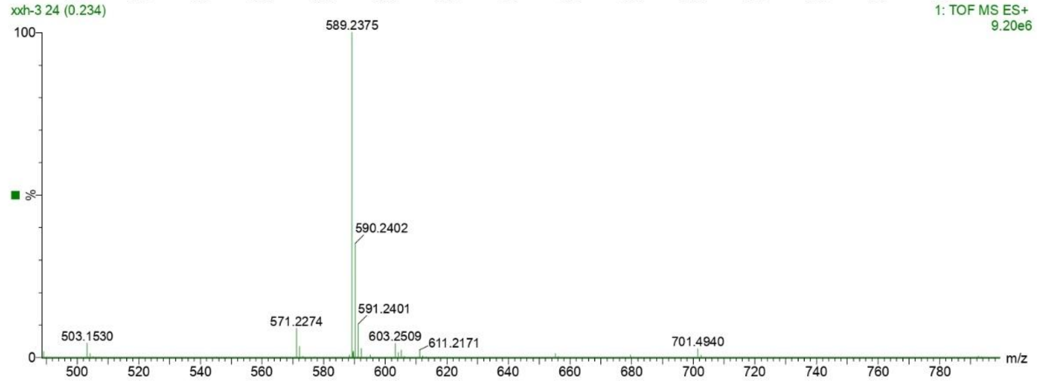
xxh-3 24 (0.234)
1: TOF MS ES+
9.20e6
589.2375
590.2402
591.2401
571.2274
503.1530
603.2509
611.2171
701.4940
%
m/z

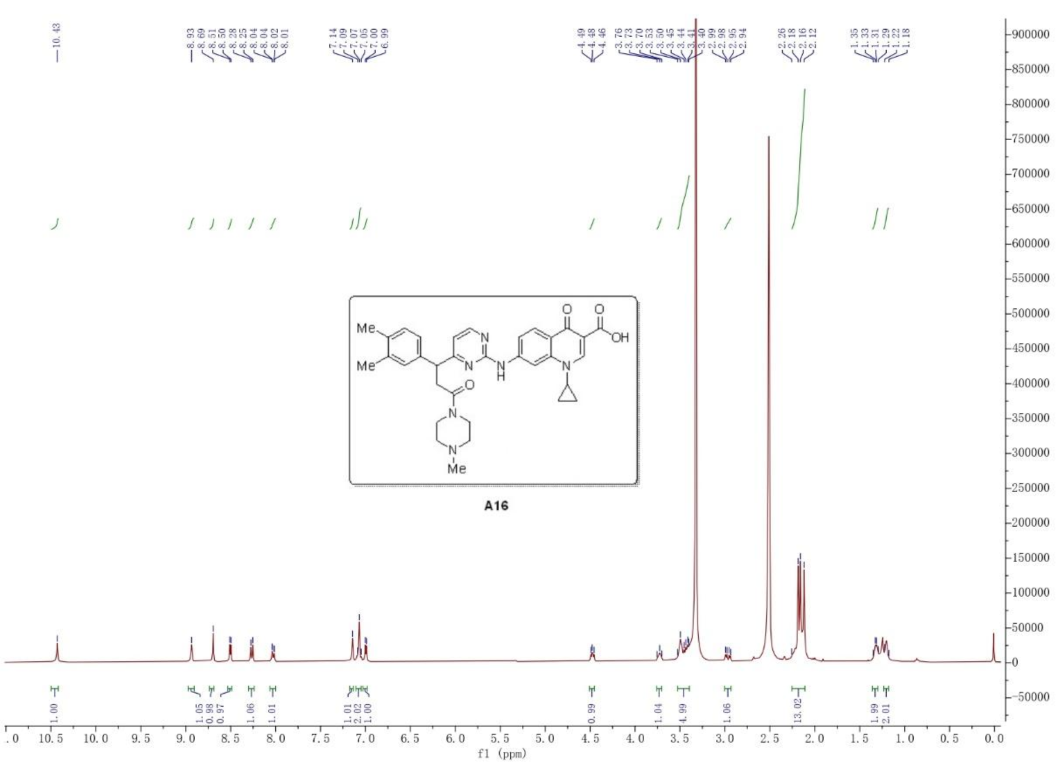
10.43
8.93
8.69
8.51
8.50
8.28
8.25
8.04
8.02
8.01
7.14
7.09
7.07
7.05
7.00
6.99
4.49
4.48
4.46
3.76
3.73
3.70
3.53
3.50
3.45
3.44
3.41
3.40
2.99
2.98
2.96
2.94
2.26
2.18
2.16
2.12
1.35
1.33
1.31
1.29
1.22
1.18
Me
Me
N
N
N
H
O
OH
O
N
O
N
N
Me
A16
1.00
1.05
0.98
0.97
1.06
1.01
1.01
2.02
1.00
0.99
1.04
4.99
1.06
13.02
1.99
2.01
f1 (ppm)

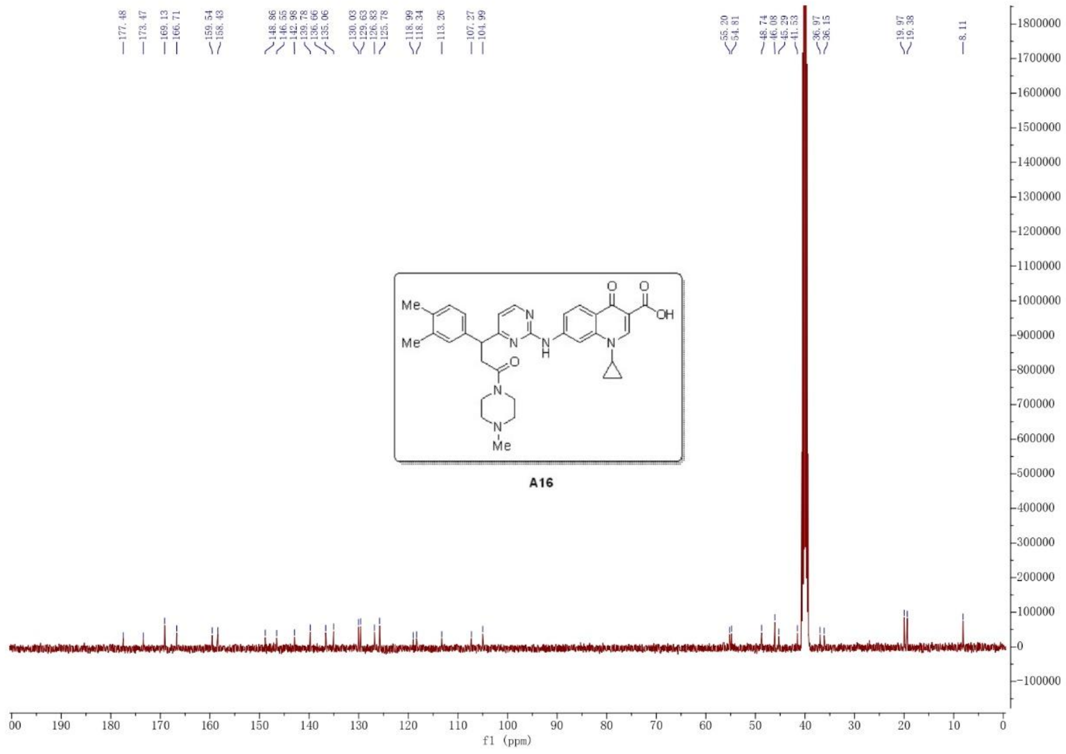
177.48
173.47
169.13
166.71
159.54
158.43
148.86
146.55
142.98
139.78
136.66
135.06
130.03
129.63
126.83
125.78
118.99
118.34
113.26
107.27
104.99
55.20
54.81
48.74
46.08
45.29
41.53
36.97
36.15
19.97
19.38
8.11
A16
f1 (ppm)

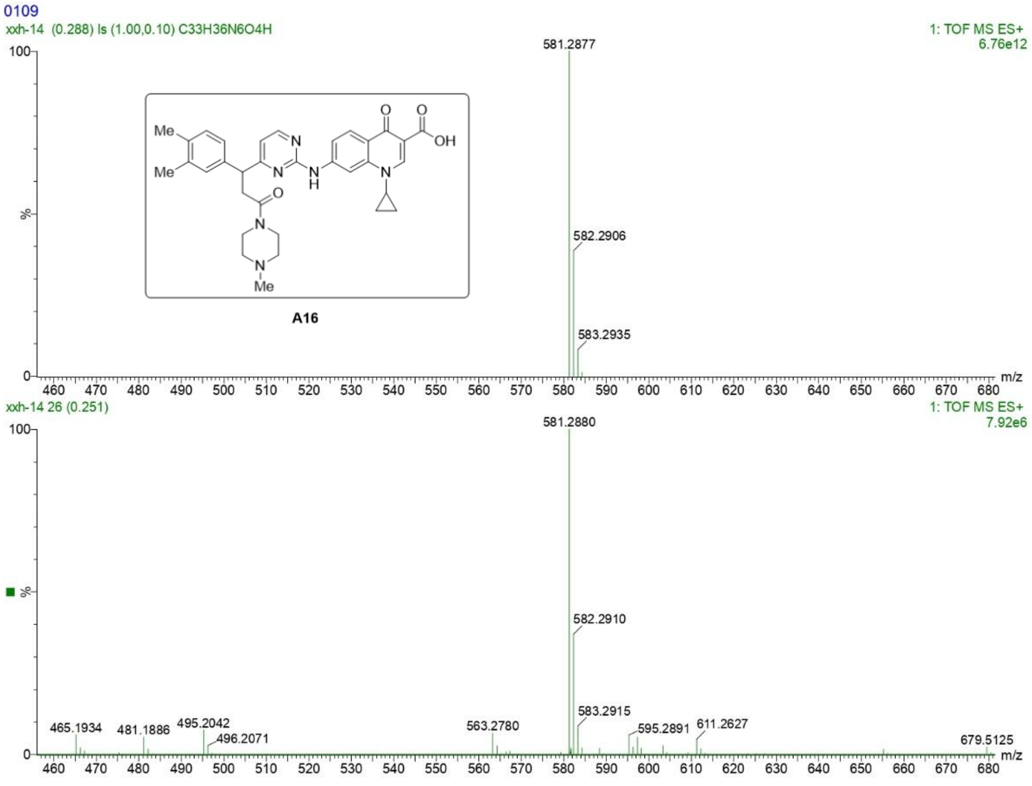
0109
xxh-14 (0.288) Is (1.00,0.10) C33H36N6O4H
1: TOF MS ES+
6.76e12
581.2877
582.2906
583.2935
A16
m/z
xxh-14 26 (0.251)
1: TOF MS ES+
7.92e6
581.2880
582.2910
583.2915
465.1934
481.1886
495.2042
496.2071
563.2780
595.2891
611.2627
679.5125
m/z

HPLC of Compound A3

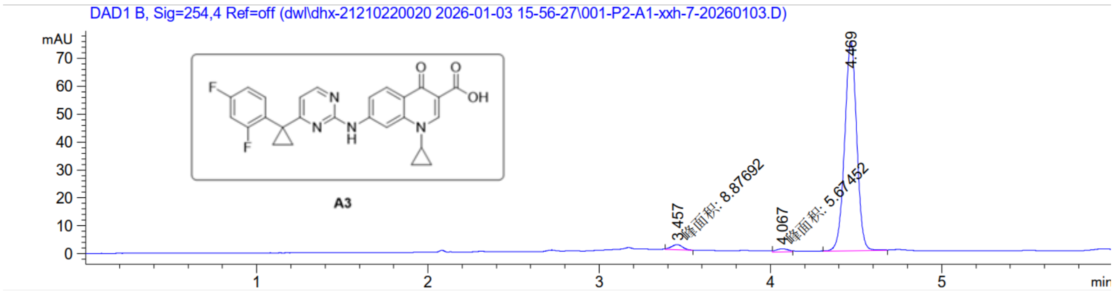

Area percentage report

Signal 2: DAD1 B, Sig = 254,4 Ref = off

Peak #	Retention time [min]	Type	Peak with [min]	Peak area [mAu*s]	Peak height [mAu]	Peak area %
1	3.457	MM	0.0786	8.87692	1.88113	2.3319
2	4.067	MM	0.0809	5.67452	1.16851	1.4907
3	4.469	BB	0.0754	366.11957	75.07966	96.1774
Total				380.67101	78.12930	

HPLC of Compound A5

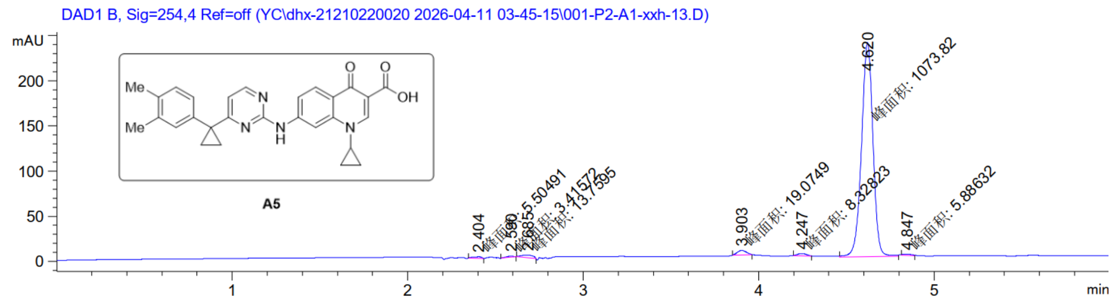

Area percentage report

Signal 2: DAD1 B, Sig = 254,4 Ref = off

Peak #	Retention time [min]	Type	Peak with [min]	Peak area [mAu*s]	Peak height [mAu]	Peak area %
1	2.404	MM	0.0519	5.50491	1.76767	0.4872
2	2.590	MM	0.0523	3.41572	1.08855	0.3023
3	2.685	MM	0.0780	13.75496	2.93874	1.2179
4	3.903	MM	0.0619	19.07488	5.13592	1.6884
5	4.247	MM	0.0582	8.32823	2.38523	0.7371
6	4.620	MM	0.0756	1073.82288	236.62685	95.0460
7	4.847	MM	0.0607	5.88632	1.61588	0.5210
Total				1129.79241	251.55884	
